# An efficient synthesis of new imidazo[1,2-*a*]pyridine-6-carbohydrazide and pyrido[1,2-*a*]pyrimidine-7-carbohydrazide derivatives *via* a five-component cascade reaction†

**DOI:** 10.1039/c9ra00350a

**Published:** 2019-03-05

**Authors:** Hajar Hosseini, Mohammad Bayat

**Affiliations:** Department of Chemistry, Faculty of Science, Imam Khomeini International University Qazvin Iran bayat_mo@yahoo.com m.bayat@sci.ikiu.ac.ir +98 28 33780040

## Abstract

A highly efficient and straightforward synthesis of N-fused heterocyclic compounds including *N*′-(1-(4-nitrophenyl)ethylidene)imidazo[1,2-*a*]pyridine-6-carbohydrazide and *N*′-(1-(4-nitrophenyl)ethylidene)pyrido[1,2-*a*]pyrimidine-7-carbohydrazide derivatives is successfully achieved *via* a five-component cascade reaction utilizing cyanoacetohydrazide, 4-nitroacetophenone, 1,1-bis(methylthio)-2-nitroethylene and various diamines in a mixture of water and ethanol. The new efficient domino protocol involving a sequence of *N*,*N*-acetal formation, Knoevenagel condensation, Michael reaction, imine–enamine tautomerization and N-cyclization as key steps. The merit of this catalyst free approach is highlighted by its easily available starting materials, operational simplicity, clean reaction profile, the use of environmentally benign solvents and tolerance of a wide variety of functional groups.

## Abstract 2

An easy synthesis of novel and highly substituted imidazo[1,2-*a*]pyridines and pyrido[1,2-*a*]pyrimidines using heterocyclic ketene aminals.

## Graphical abstract

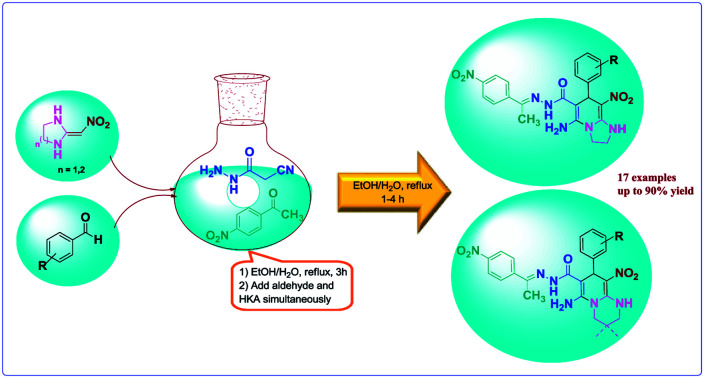

## Introduction

**Fig. 1:**
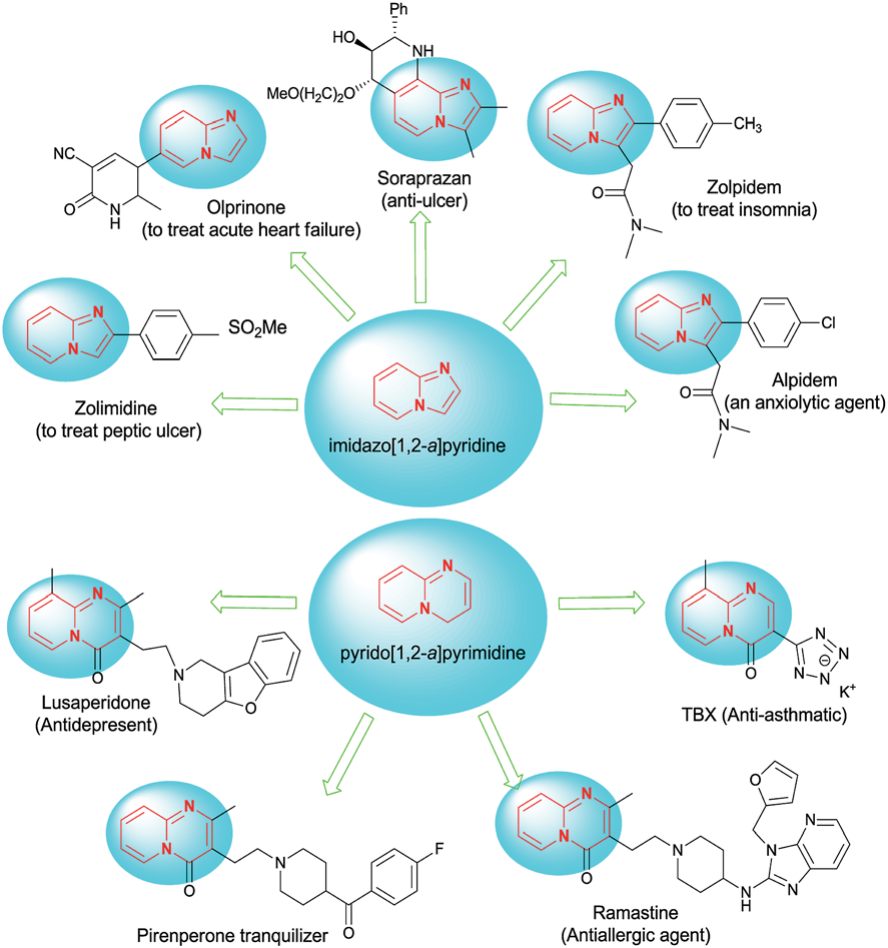
Drugs containing the imidazo[1,2-*a*]pyridine and pyrido[1,2-*a*]pyrimidine cores.

Imidazopyridines have displayed a broad spectrum of pharmacological and biological activities.^1^ Among the diverse derivatives of imidazopyridine, the imidazo[1,2-*a*]pyridine skeleton is probably the most important structure due to its vital role as a key construction in drugs and biologically active compounds with properties such as anti-inflammatory,^2,3^ antiviral,^4–6^ antifungal,^7–9^ anticancer,^10^ anxiolytic,^11^ anti-ulcer,^12^ and antiprotozoal.^13^ They can be found in marketed drugs such as the clinical anti-ulcer compound zolpidem and alpidem,^14^ olprinone,^15^ zolimidine,^16^ necopidem and saripidem^17^ (Fig. 1).

Pyridopyrimidine derivatives are also known as antidepressant,^18^ gastrointestinal protective,^19^ neurotropic and stress-protecting,^20^ and anticancer agents.^21^ pyrido[1,2-*a*]pyrimidine constitutes the core structure of some marketed drugs, including the antiasthmatic agent pemirolast,^22^ the tranquilizer pirenperone,^23^ and the antiallergic agent ramastine^24^ (Fig. 1).

The design of reactions with minimized number of steps is one of the purpose of modern synthesis. One approach to achieve this goal includes the development of multicomponent methods. Multicomponent reactions (MCRs) offer a wide range of probabilities for the fast generation of functionalized molecules in a single step with high atom economy, minimum time and cost and straight forward experimental procedures.^25^ These benefits are highlights for multicomponent cascade reactions, which involve *in situ* production of an intermediate with a reactive site for subsequent transformations.^26^

By now, various synthetic approaches have been reported to synthesize imidazo[1,2-*a*]pyridines. The common reactions were the cyclocondensations of 2-aminopyridines with α-halocarbonyl compounds,^27^ 1,3-dicarbonyl compounds,^28^ nitroolefins or alkynes.^29^ Condensation of 2-aminopyridines, aldehydes and isonitriles or alkynes in a one-pot process, was also a convenient method for the synthesis of imidazo[1,2-*a*]pyridines.^30^ Furthermore, over the past decade, a number of methods have been described to synthesize pyrido[1,2-*a*]pyrimidines by focusing on traditional two-component condensation of 2-aminopyridines with a variety of bifunctional electrophiles.^31–33^

In recent years some other new synthetic approaches have been developed for the synthesis of tetrahydroimidazo[1,2-*a*]pyridines and tetrahydro-1*H*-pyrido[1,2-*a*]pyrimidine using heterocyclic ketene aminals (HKAs).^34–38^ Heterocyclic ketene aminals (HKAs) are efficient synthons for the synthesis of heterocyclic compounds. Reactions of cyclic ketene aminals with a variety of bis-electrophilic compounds have so far been applied to construct five- and six-membered fused heterocyclic structures.^39^

In the process of our efforts to synthesize the new heterocyclic compounds using cyanoacetohydrazide, we report herein an efficient one-pot five-component synthesis of novel imidazo[1,2-*a*]pyridine-6-carbohydrazides and 1*H*-pyrido[1,2-*a*]pyrimidine-7-carbohydrazides *via in situ* preparation of nitroketene aminals. To the best of our knowledge, there is no report on the synthesis of these structures.

## Results and discussion

We succeeded in synthesizing two categories of functionalized N-fused heterocyclic structures containing tetrahydroimidazo[1,2-*a*]pyridines-6-carbohydrazides and tetrahydro-1*H*-pyrido[1,2-*a*]pyrimidine-7-carbohydrazides by using of cyanoacetohydrazide 1, 4-nitroacetophenone 2, aromatic aldehydes 3, 1,1-bis(methylthio)-2-nitroethene 4 and various diamines 5.

### Optimization of the conditions

Initially, cyanoacetohydrazide 1, 4-nitroacetophenone 2, 4-chlorobenzaldehyde 3, 1,1-bis(methylthio)-2-nitroethene 4 and ethylenediamine 5 were used as model substrates to optimize the reaction conditions.

The experimental results showed when ethanol was used as solvent without any catalyst at reflux conditions, the yield of desired product was 60% (Table 1, entry 1). With piperidine as catalyst, the reaction efficiency did not change significantly (entry 2). With *p*-TSA and acetic acid, the product did not form (entry 3, 4). The use of water or acetonitrile instead of ethanol did not result in the desired product (entry 5, 8), but when the mixture of water and ethanol was used (overall 1 : 2, v/v), the efficiency increased slightly (entry 6). By changing the ratio of solvents (1 : 3, v/v) at reflux conditions the five-component product 6c was obtained in a yield of 87% within 5 hours (entry 7).

**Table 1:** Optimization conditions for the formation of 6c^a^

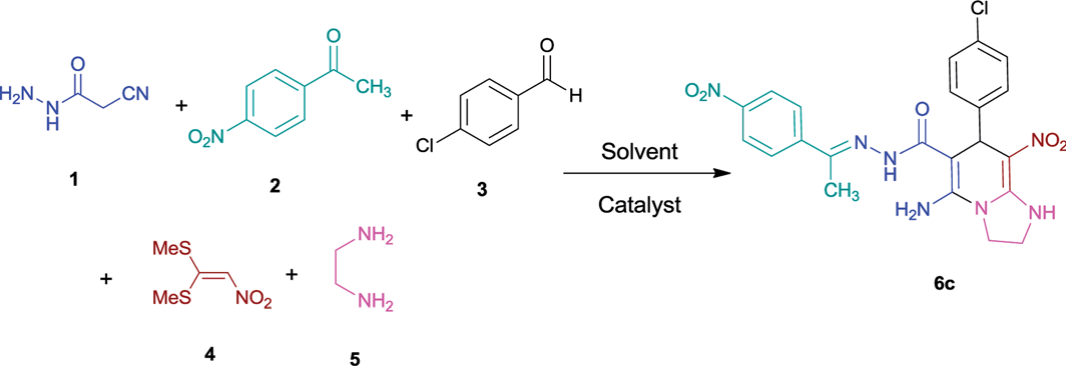					
Entry	Solvent	Catalyst (mol%)	Time (h)	Temp (°C)	Yield (%)
1	EtOH	—	24	78	60
2	EtOH	Piperidine	24	78	57
3	EtOH	*p*-TSA	24	78	No reaction
4	EtOH	AcOH	24	78	No reaction
5	H_2_O	—	24	100	No reaction
6	H_2_O/EtOH (1 : 2, v/v)	—	10	78	65
**7**	**H**_**2**_ **O/EtOH (1** **:** **3, v/v)**	**—**	**5**	**78**	**87**
8	CH_3_CN	—	24	82	No reaction

a Reagents and conditions: cyanoacetohydrazide (1 mmol), 4-nitroacetophenone (1 mmol), 4-chlorobenzaldehyde (1 mmol), 1,1-bis(methylthio)-2-nitroethene (1 mmol), ethylenediamine (1 mmol), solvent (20 mL), catalyst (0.2 mmol). The bold row represents the best results.

**Scheme 1:**
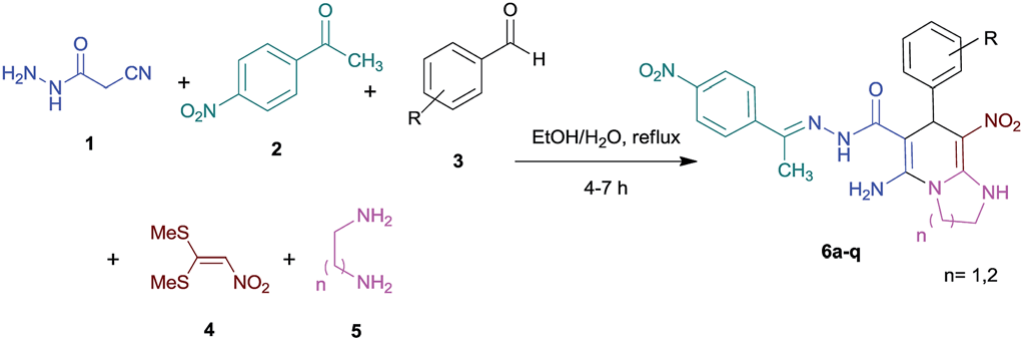
Synthetic scheme for the generation of products 6a–q.

With information obtained from optimization conditions table, we used cyanoacetohydrazide 1, 4-nitroacetophenone 2, various aromatic aldehydes 3, 1,1-bis(methylthio)-2-nitroethene 4 and diamines (ethylenediamine, 1,3-diaminopropane, 2,2-dimethyl-1,3-diaminopropane) 5 as starting materials to synthesize the target compounds 6a–q (Scheme 1).

The reactions were completed after 4–7 h overall to afford corresponding heterocyclic systems in good to high yields (73–90%). The results are summarized in Table 2.

**Table 2:** Compounds 6a–q^a^

Entry	Aromatic aldehyde	Diamine	Product	Time (h)	Yield (%)	Mp (°C)
1	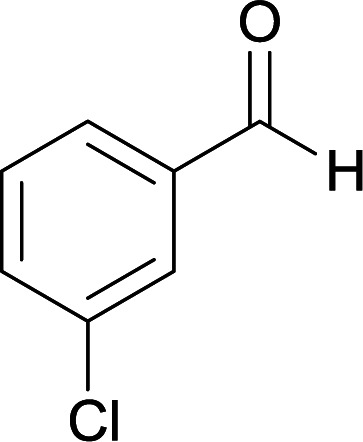	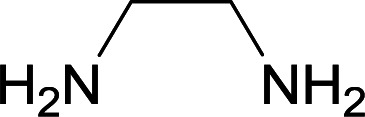	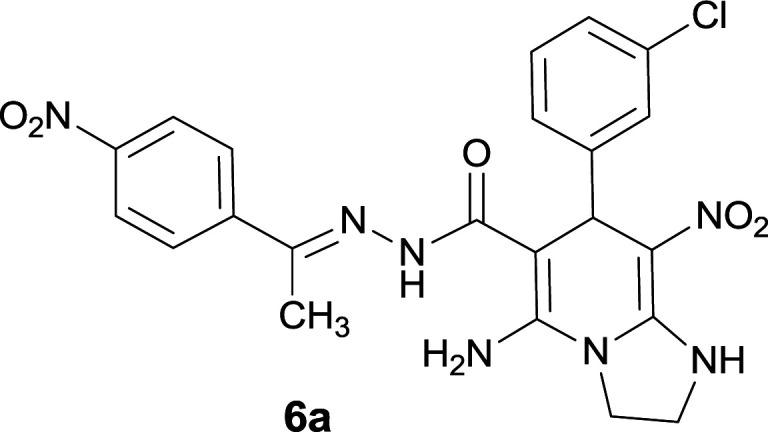	6	90	231–233
2	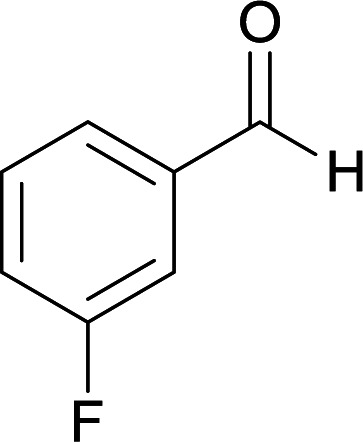	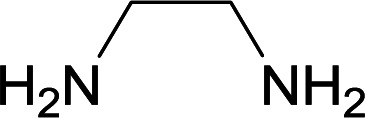	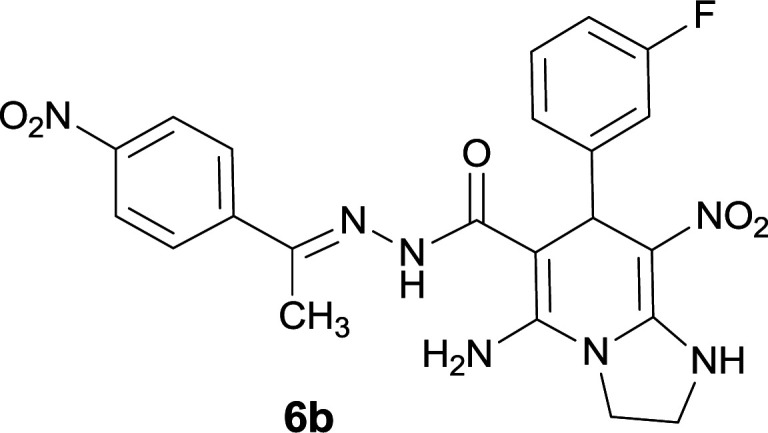	5	86	235–237
3	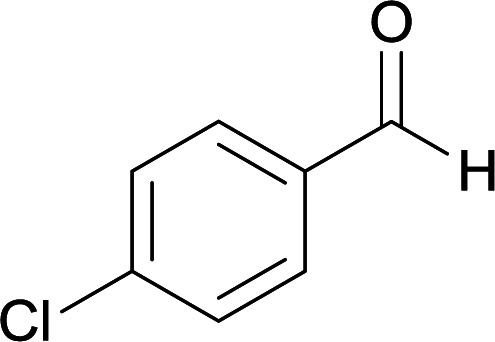	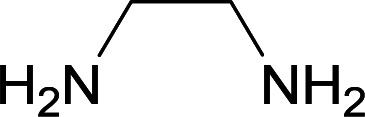	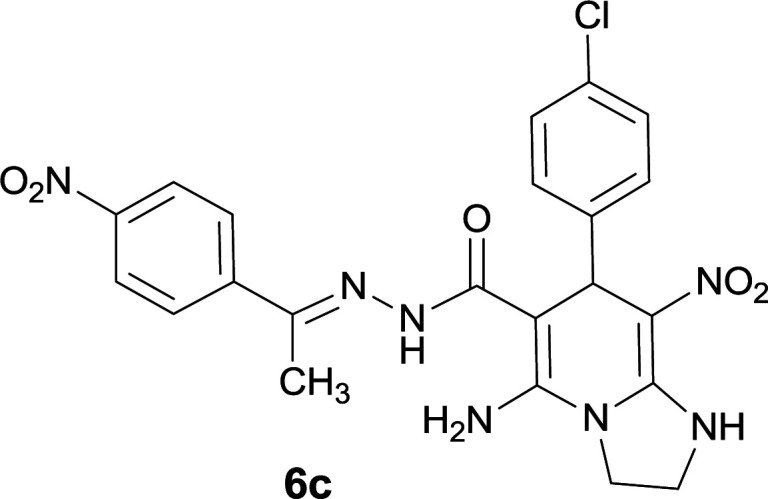	5	87	239–241
4	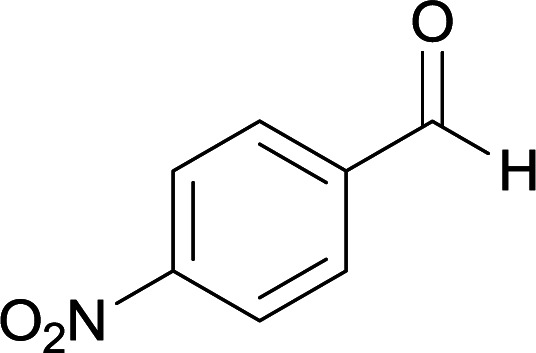	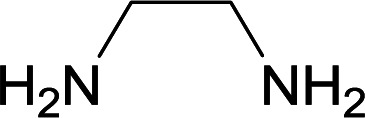	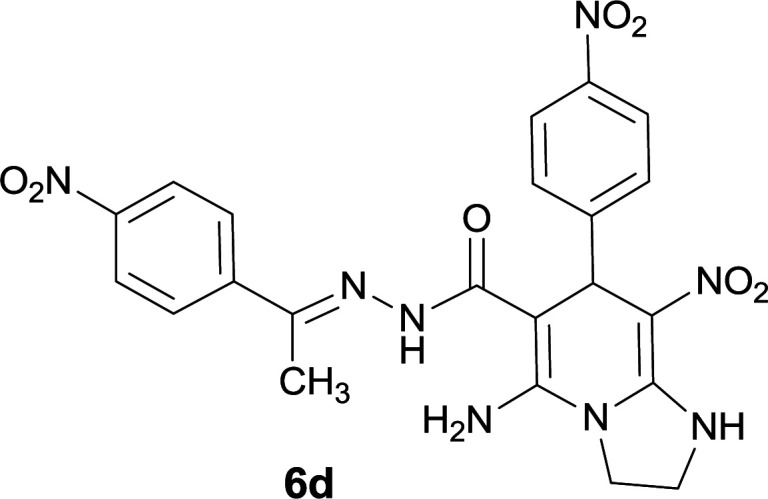	4	89	243–245
5	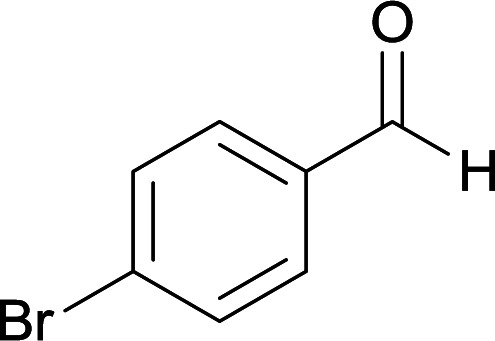	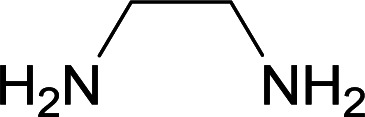	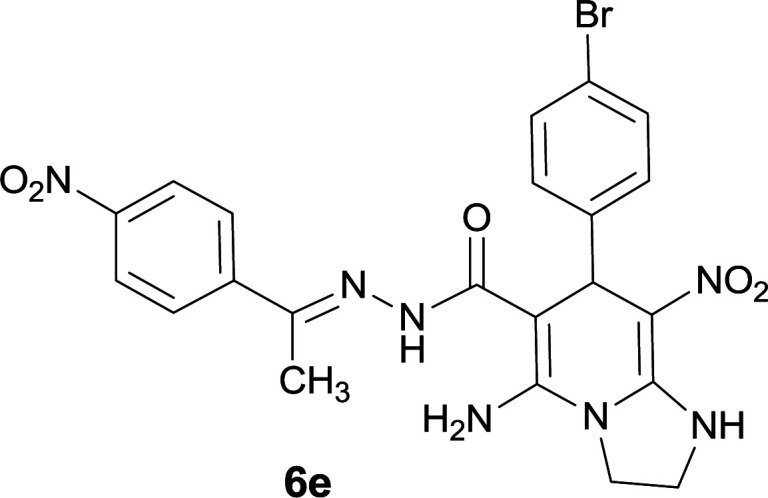	5	90	239–240
6	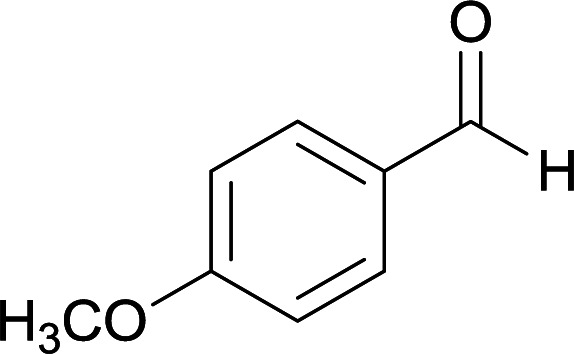	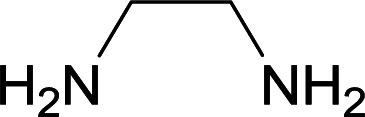	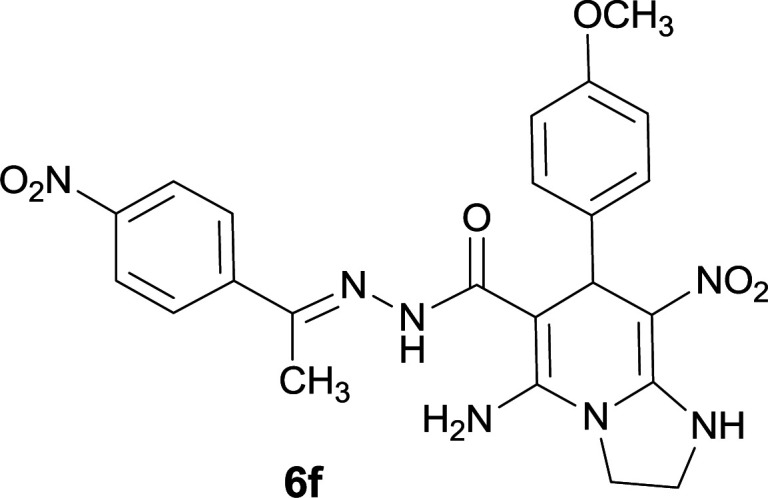	7	78	267–269
7	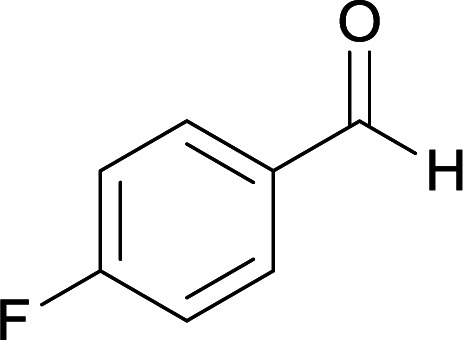	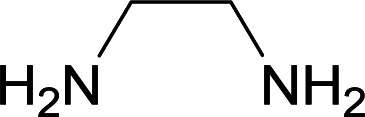	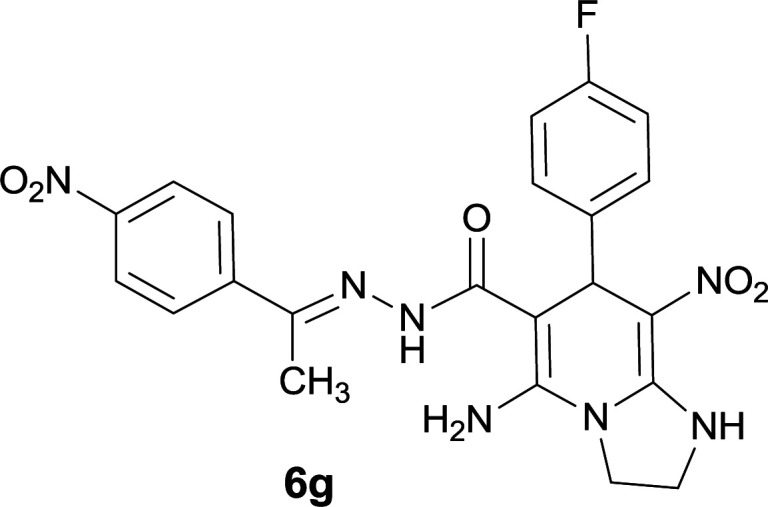	5	80	271–273
8	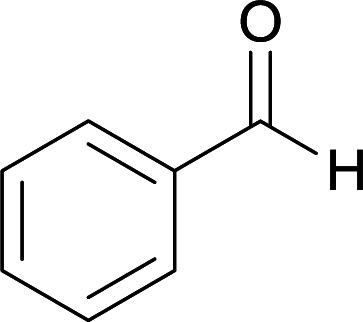	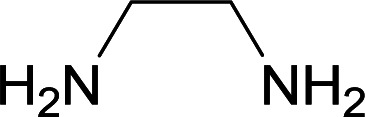	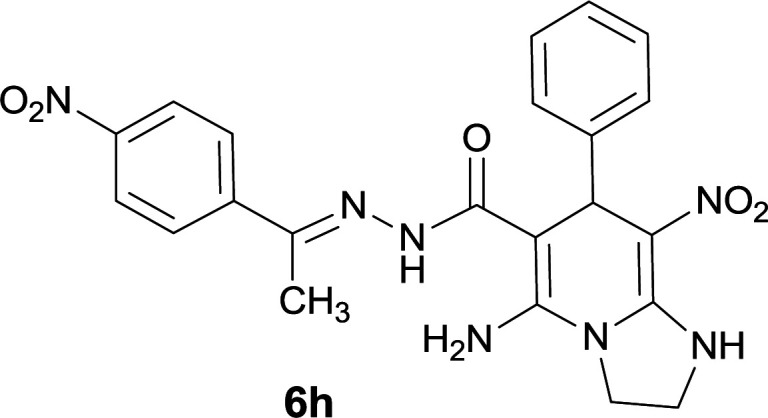	6	75	284–286
9	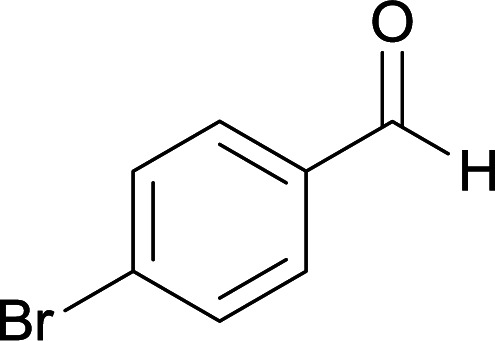	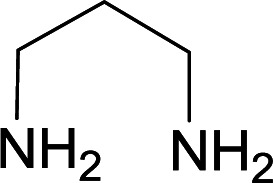	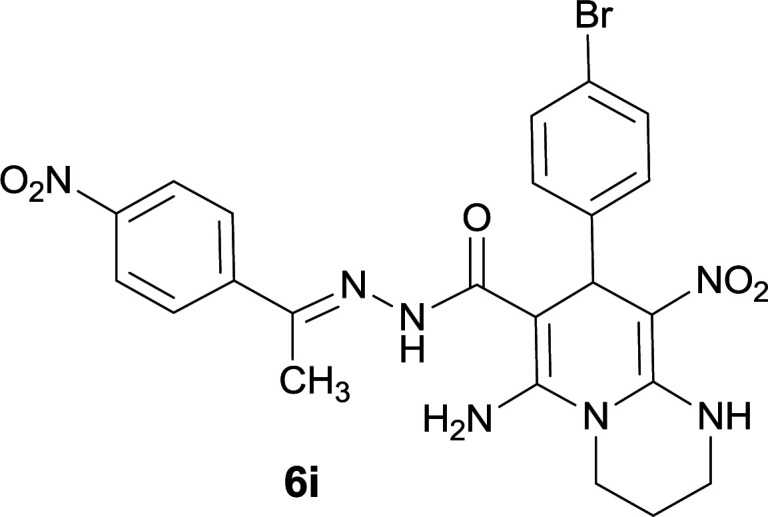	5	86	264–266
10	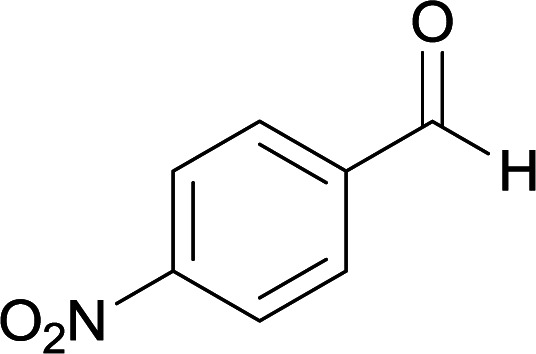	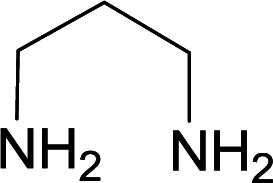	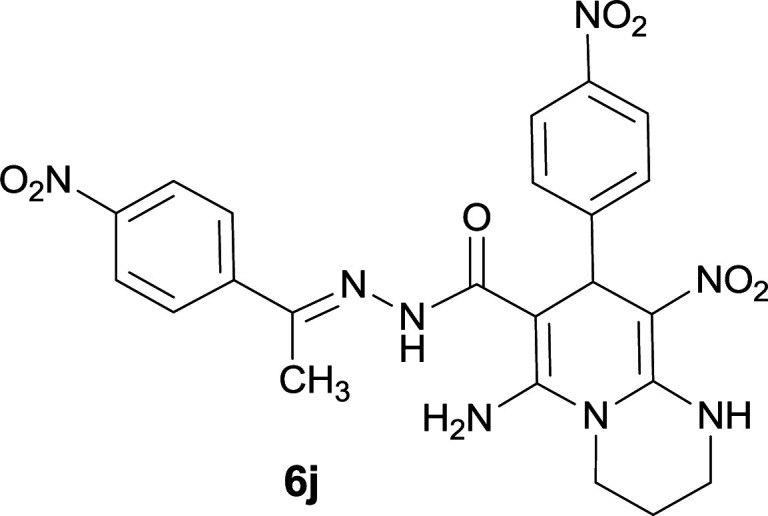	4	88	268–270
11	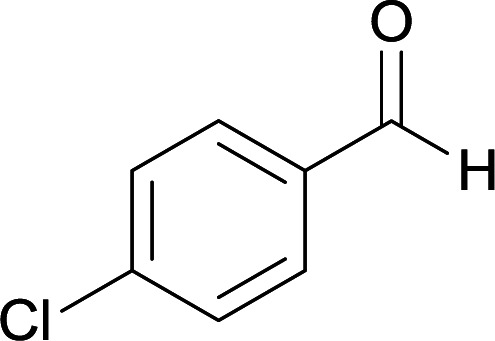	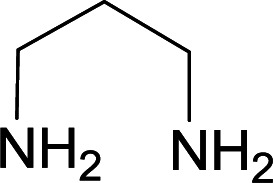	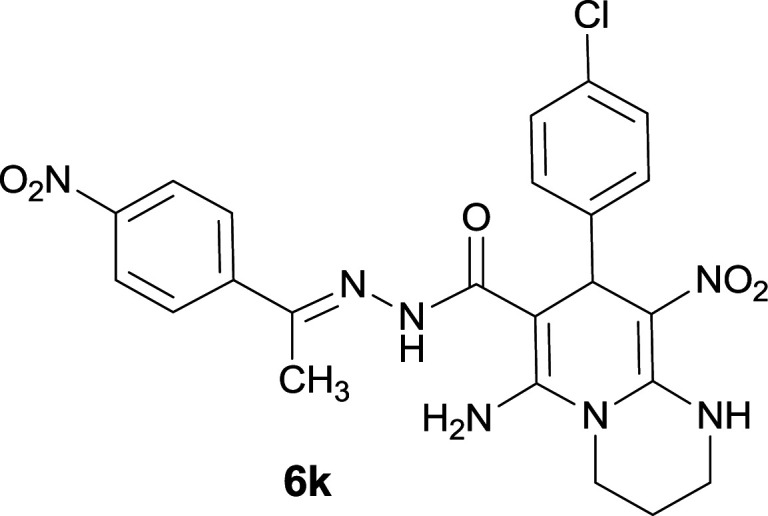	5	85	265–267
12	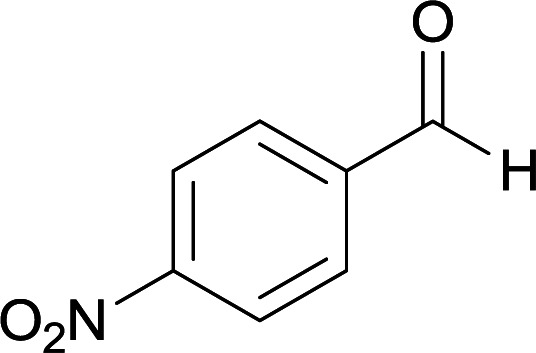	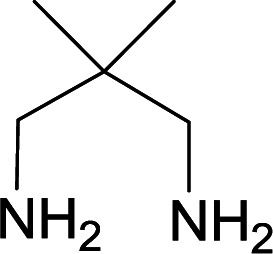	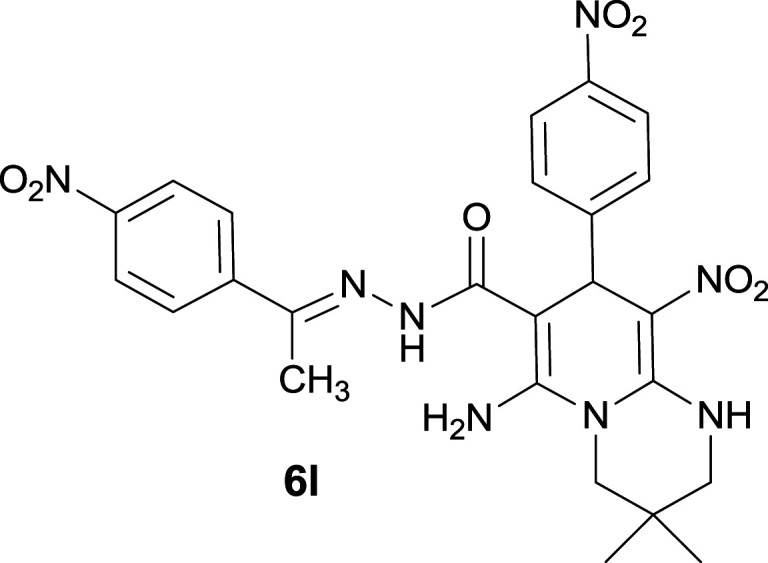	4	90	298–300
13	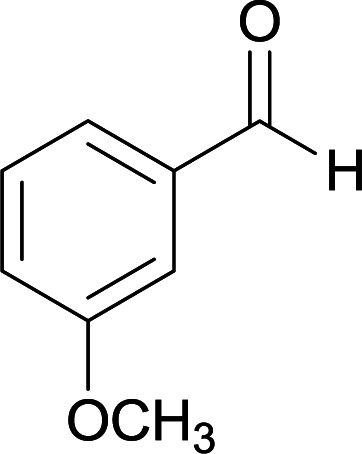	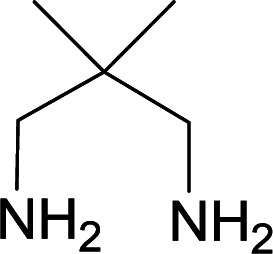	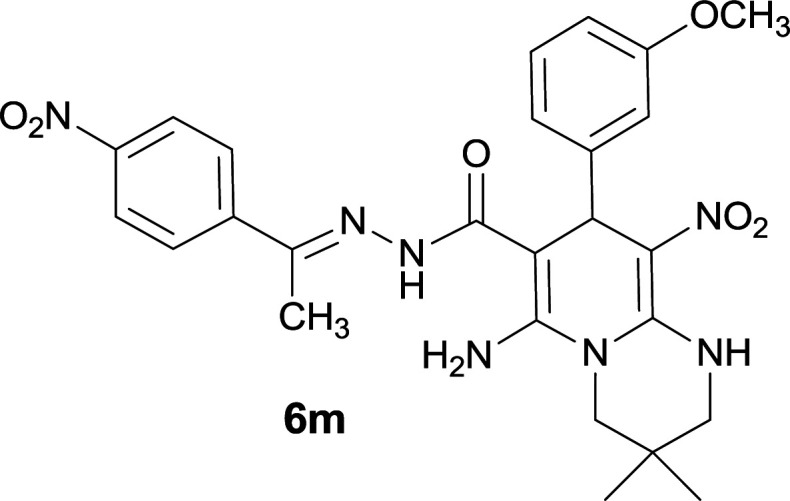	7	73	269–271
14	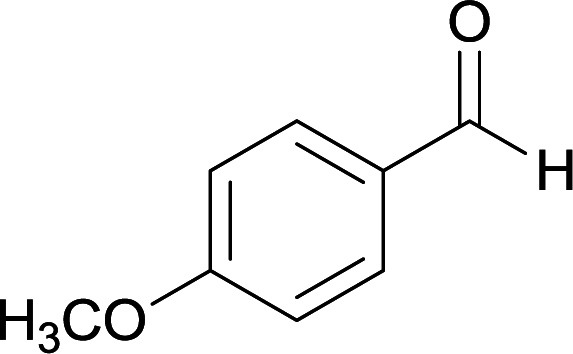	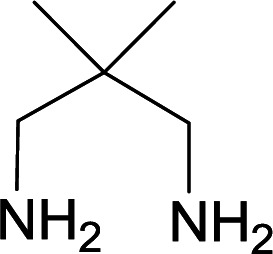	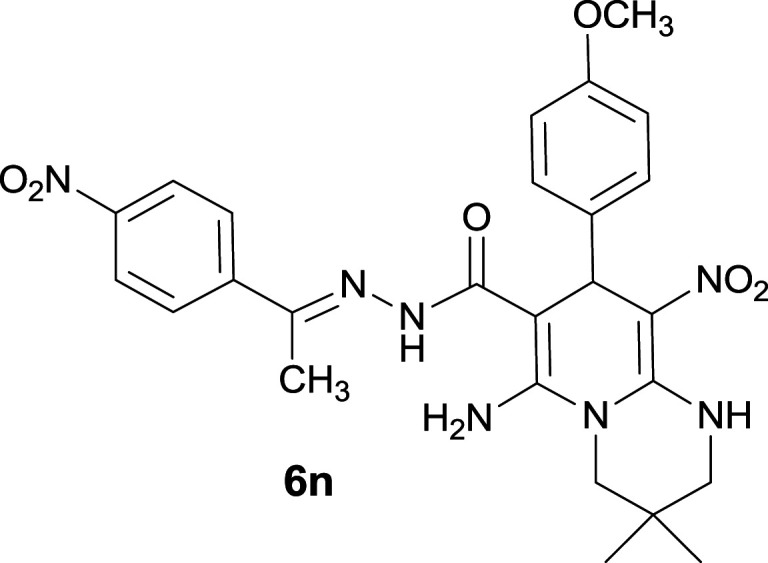	7	75	315–317
15	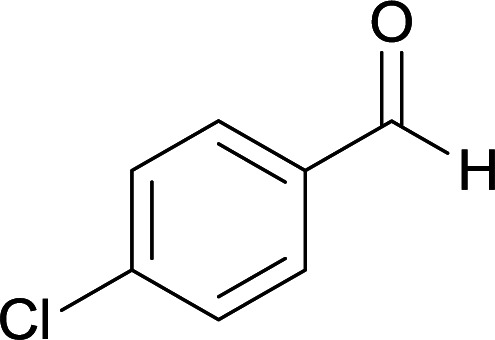	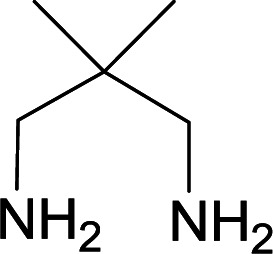	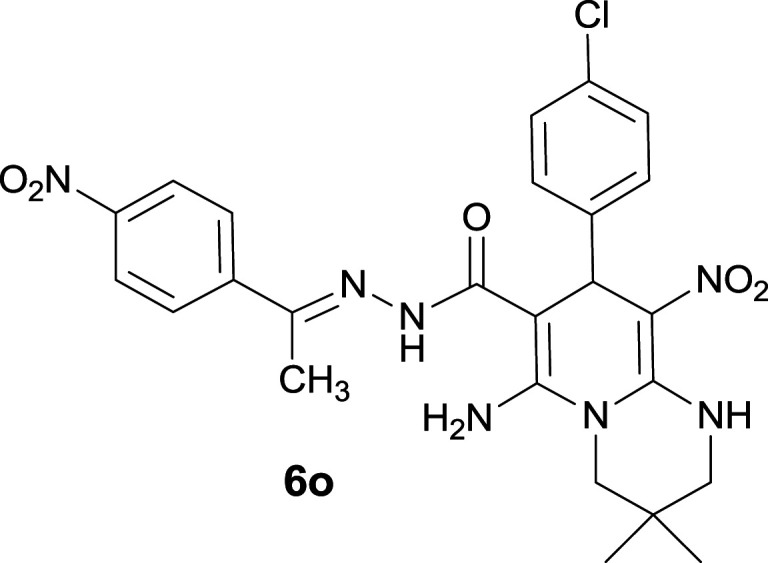	5	85	317–319
16	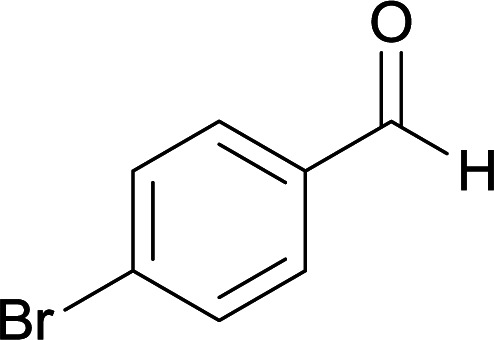	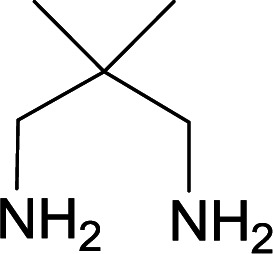	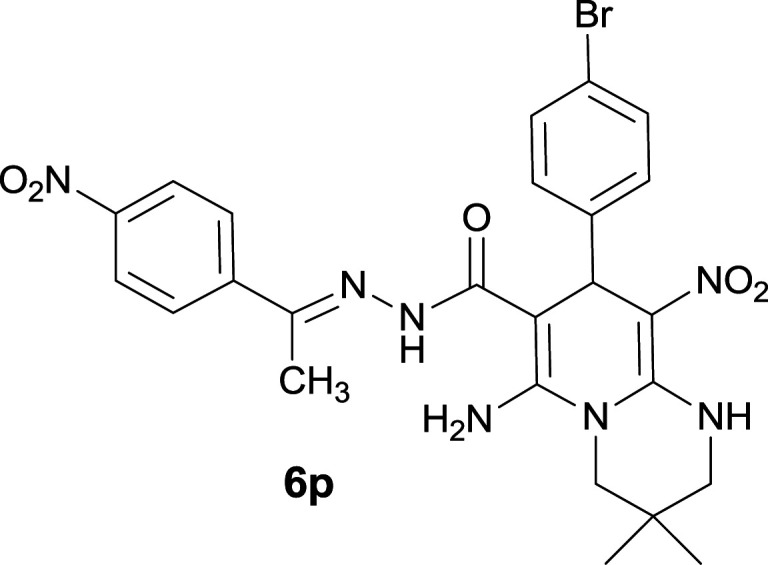	5	84	322–324
17	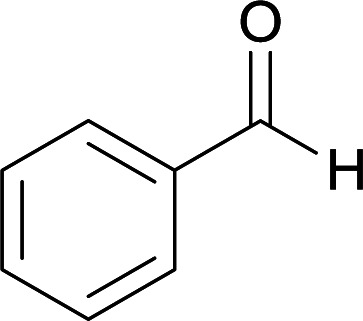	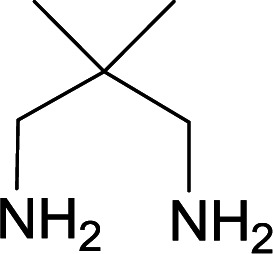	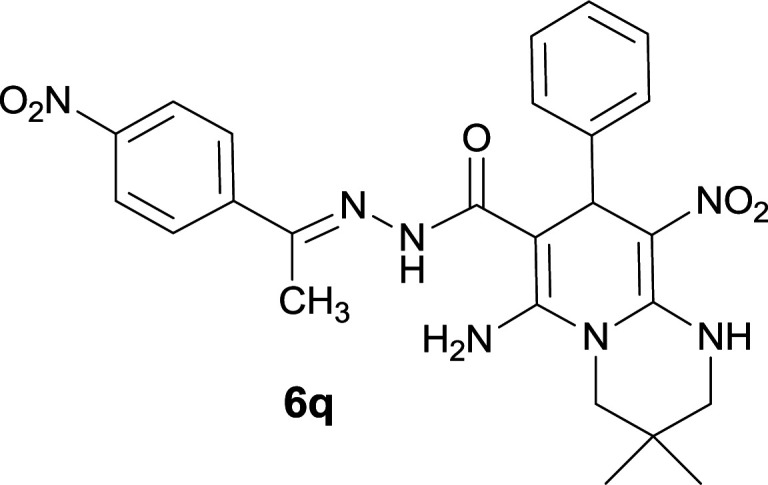	6	75	329–331

a The reaction was performed using cyanoacetohydrazide (1 mmol), 4-nitroacetophenone (1 mmol), aromatic aldehyde (1 mmol), 1,1-bis(methylthio)-2-nitroethene (1 mmol), diamine (1 mmol), EtOH (15 mL), H_2_O (5 mL), reflux.

### Effect of substituents

From the observation of reaction times in Table 2, it was found that with aldehydes containing an electron-withdrawing group on *para* position of ring (nitro and halogens), the reaction rate is the highest and with methoxy group, the rate is the lowest.

This reaction was performed with other derivatives of diamines (1,4-diaminobutane and 1,2-diaminocyclohexane) under the same conditions, which did not result in the product. Also the reaction with *ortho* derivatives of benzaldehyde (2-chloro and 2-nitro) did not produce the desired product. Other derivatives of acetophenone (4-chloro, 4-bromo and 4-methoxyacetophenone) were also used which resulted no product formation.

In addition, the reaction with aliphatic ketones instead of 4-nitroacetophenone and aliphatic aldehydes instead of aromatic aldehydes did not lead to the desired products.

It should be noted that these reactions involve two-, three- and four-component impurities. In fact, the most important side product was a four-component structure without participation of 4-nitroacetophenone that was previously synthesized using two equivalents of aldehyde.^34^ To achieve the pure product, it was necessary to complete the reaction of cyanoacetohydrazide and 4-nitroacetophenone in a mixture of water and ethanol at reflux conditions in sufficient time (3 hours), then ketene aminal solution and aromatic aldehyde were added at the same time.

### Structure determination

The structures of compounds 6a–q were deduced from their IR, ^1^H NMR, ^13^C NMR spectroscopic and Mass spectrometric data (see the ESI†).

**Fig. 2:**
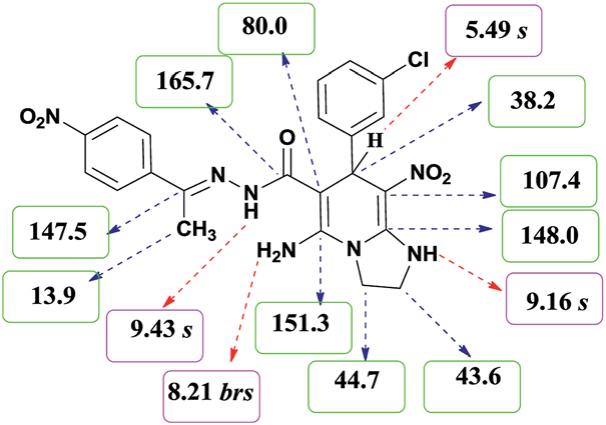
^1^H and ^13^C NMR chemical shifts of 6a.

The formation of suggested products 6a–q is clearly verified by the ^1^H and ^13^C NMR spectra of the crude products. As a representative case the key signals of ^1^H and ^13^C NMR chemical shifts of 5-amino-7-(3-chlorophenyl)-8-nitro-*N*′-(1-(4-nitrophenyl)ethylidene)-1,2,3,7-tetrahydroimidazo[1,2-*a*]pyridine-6-carbohydrazide 6a are presented in Fig. 2.

The ^1^H NMR spectrum of 6a showed two NH groups at *δ* 9.16 and 9.43 ppm. The protons of 4-nitrobenzene ring were seen at *δ* 7.96 and 8.20 ppm as two doublet signals and the protons of 3-chlorobenzene ring were observed at *δ* 6.92–7.31 ppm. The NH_2_ group appeared at *δ* 8.21 ppm. The proton of CH at pyridine ring was seen at *δ* 5.49 ppm. Two protons of two methylene groups appeared at *δ* 3.81 and 4.05 ppm. The signal at *δ* 2.14 ppm was related to methyl group.

The ^1^H-decoupled ^13^C NMR spectrum of 6a indicated 20 distinct resonances in accordance to desired structure. The characteristic signals of four aliphatic carbons (CH_3_, CH and two CH_2_ groups) were seen at *δ* 13.9, 38.2, 43.6 and 44.7 ppm respectively. Two signals at *δ* 80.0 and 107.4 ppm were related to C=*C*–CO and C–NO_2_ respectively. The carbonyl group appeared at *δ* 165.78 ppm (Fig. 2).

The mass spectrum of 6a displayed the molecular-ion peak at *m*/*z* 497 in agreement with the proposed structure. The IR spectrum of this compound showed absorption bands at 3486, 3400, 3327 cm^−1^ due to NH and NH_2_ groups, stretching vibration of aliphatic C–H bands at 2909, and strong absorption of carbonyl group at 1658 and C–N band at 1254 cm^−1^. Two absorption bands due to nitro group appeared at 1519 and 1375 cm^−1^.

### Mechanism

**Scheme 2:**
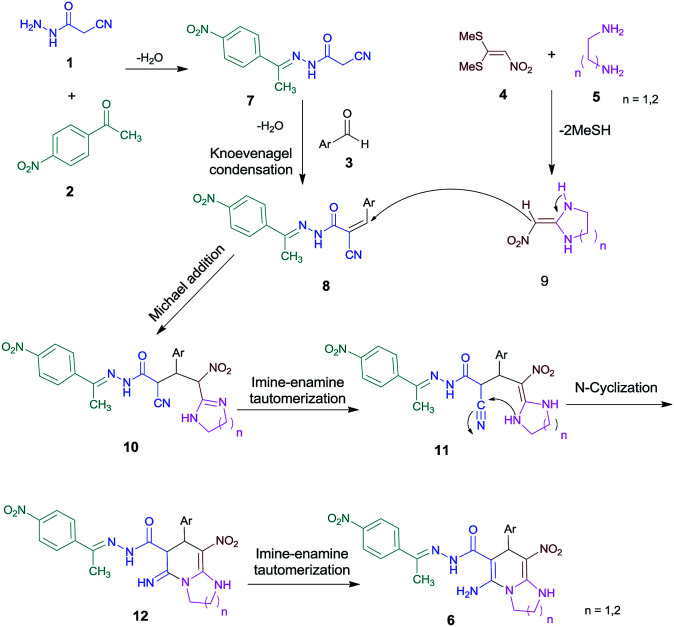
Proposed mechanism for the formation of products 6.

A general reasonable mechanism for the formation of imidazo[1,2-*a*]pyridines-6-carbohydrazides and pyrido[1,2-*a*]pyrimidine-7-carbohydrazides is shown in Scheme 2. On the basis of well-established chemistry of 1,1-bis(methylthio)-2-nitroethene, initially, addition of diamine 5 to 1,1-bis(methylthio)-2-nitroethene 4 leads to the formation of ketene aminal 9. On the other hand condensation of cyanoacetohydrazide 1 with 4-nitroacetophenone 2 leads to hydrazone 7. Further, with adding aldehyde 3, the Knoevenagel condensation affords intermediate 8. Then, Michael addition of ketene aminal 9 to adduct 8 leads to the intermediate 10, which undergoes successive imine–enamine tautomerization followed by an intramolecular cyclization *via* nucleophilic addition of –NH to nitrile group. Finally, imine–enamine tautomerization gives the titled products 6 (Scheme 2).

## Experimental

### Materials

All commercially available reagents and other solvents were purchased from Aldrich and Merck chemical Co. and used without further purification. The NMR spectra were recorded with a Bruker DRX-300 AVANCE instrument (300 MHz for ^1^H and 75.4 MHz for ^13^C) with DMSO-*d*_6_ as solvent. Chemical shifts are given in ppm (*δ*), and coupling constant (*J*) are reported in Hertz (Hz). Melting points were measured with an electrothermal 9100 apparatus. Mass spectra were recorded with an Agilent 5975C VL MSD with Triple-Axis detector operating at an ionization potential of 70 eV. IR spectra were measured with Bruker Tensor 27 spectrometer. Elemental analyses for C, H and N were performed using a PerkinElmer 2004 series [II] CHN elemental analyzer.

### General procedure of the synthesis of tetrahydroimidazo[1,2-*a*]pyridine-6-carbohydrazide and tetrahydro-1*H*-pyrido[1,2-*a*]pyrimidine-7-carbohydrazide derivatives

A mixture of diamine (1 mmol), 1,1-bis(methylthio)-2-nitroethylene (0.165 g, 1 mmol) and 10 mL EtOH in a 50 mL flask was refluxed for 6 hours. In another 50 mL flask the stoichiometric mixture of cyanoacetohydrazide (1 mmol, 0.099 g) and 4-nitroacetophenone (1 mmol, 0.165 g) in H_2_O/EtOH (5 : 5 mL) was stirred at reflux conditions for 3 hours. After this time, TLC shows the consumption of the starting components. Then, aromatic aldehyde (1 mmol) and the first solution (HKA), was added to this mixture simultaneously. The progress of the reaction was monitored by TLC using ethyl acetate/*n*-hexane (1 : 1). After completion of the reaction, the precipitated product was collected by filtration and washed with warm ethanol to give the pure products 6a–q in 73–90% yield.

#### 5-Amino-7-(3-chlorophenyl)-8-nitro-*N*′-(1-(4-nitrophenyl)ethylidene)-1,2,3,7-tetrahydroimidazo[1,2-*a*]pyridine-6-carbohydrazide (6a)

Dark yellow solid; yield: 0.447 g (90%); mp: 231–233 °C; IR (KBr) (*ν*_max_/cm^−1^): 3486, 3400, 3327, 2909, 1658, 1519, 1458, 1375, 1254, 785; ^1^H NMR (300 MHz, DMSO-*d*_6_): *δ* 2.14 (3H, s, CH_3_), 3.78–3.84 (2H, m, CH_2_), 3.99–4.11 (2H, m, CH_2_), 5.49 (1H, s, CH), 6.92–7.31 (4H, m, ArH), 7.96 (2H, d, *J* = 8.7 Hz, ArH), 8.20 (2H, d, *J* = 8.7 Hz, ArH), 8.21 (2H, br s, NH_2_), 9.16 (1H, s, NH), 9.43 (1H, s, NH); ^13^C{^1^H} NMR (75.4 MHz, DMSO-*d*_6_): *δ* 13.9 (CH_3_), 38.2 (CH), 43.6 (CH_2_–NH), 44.7 (CH_2_–N), 80.0 (C=**C**–CO), 107.4 (C=**C**–NO_2_), 123.9, 126.8, 127.1, 127.3, 128.3, 130.3, 132.8, 145.0, 147.3 (Ar), 147.5 (Me–**C**=N), 148.0 (C=**C**–NH), 151.3 (C_Ar_–NO_2_), 151.4 (C=**C**–NH_2_), 165.7 (C=O); MS (EI, 70 eV): *m*/*z* (%) = 497 (0.008) [M]^+^, 454 (2), 326 (59), 311 (100), 292 (29), 265 (37), 246 (51), 204 (26), 179 (63), 117 (83), 76 (47), 57 (7); anal. calcd for C_22_H_20_ClN_7_O_5_: C, 53.07; H, 4.05; N, 19.69. Found: C, 53.37; H, 3.84; N, 19.65.

#### 5-Amino-7-(3-fluorophenyl)-8-nitro-*N*′-(1-(4-nitrophenyl)ethylidene)-1,2,3,7-tetrahydroimidazo[1,2-*a*]pyridine-6-carbohydrazide (6b)

Orange solid; yield: 0.413 g (86%); mp: 235–237 °C; IR (KBr) (*ν*_max_/cm^−1^): 3477, 3332, 3401, 1660, 1519, 1462, 1377, 1331, 1254; ^1^H NMR (300 MHz, DMSO-*d*_6_): *δ* 2.15 (3H, s, CH_3_), 3.37–3.84 (2H, m, CH_2_), 4.02–4.12 (2H, m, CH_2_), 5.47 (1H, s, CH), 7.17–7.39 (4H, m, ArH), 7.96 (2H, d, *J* = 8.7 Hz, ArH), 8.20 (2H, d, *J* = 8.7 Hz, ArH), 8.24 (2H, br s, NH_2_), 9.17 (1H, s, NH), 9.45 (1H, s, NH); ^13^C{^1^H} NMR (75.4 MHz, DMSO-*d*_6_): *δ* 13.9 (CH_3_), 38.1 (CH), 43.6 (CH_2_–NH), 44.7 (CH_2_–N), 80.1 (C=**C**–CO), 107.5 (C=**C**–NO_2_), 113.7, 115.2, 123.9, 124.4, 127.3, 130.2, 145.0, 147.5 (Ar), 148.5 (Me–**C**=N), 148.5 (C=**C**–NH), 151.4 (C_Ar_–NO_2_), 151.5 (C=**C**–NH_2_), 160.7 (C_Ar_–F), 165.8 (C=O); MS (EI, 70 eV): *m*/*z* (%) = 481 (0.008) [M]^+^, 438 (6), 392 (2), 326 (57), 311 (100), 276 (62), 230 (87), 179 (54), 117 (71), 76 (39), 50 (5); anal. calcd for C_22_H_20_FN_7_O_5_: C, 54.88; H, 4.19; N, 20.37. Found: C, 54.56; H, 4.04; N, 20.53.

#### 5-Amino-7-(4-chlorophenyl)-8-nitro-*N*′-(1-(4-nitrophenyl)ethylidene)-1,2,3,7-tetrahydroimidazo[1,2-*a*]pyridine-6-carbohydrazide (6c)

Yellow solid; yield: 0.432 g (87%); mp: 239–241 °C; IR (KBr) (*ν*_max_/cm^−1^): 3456, 3401, 3339, 2919, 1675, 1522, 1456, 1373, 1255, 761; ^1^H NMR (300 MHz, DMSO-*d*_6_): *δ* 2.15 (3H, s, CH_3_), 3.77–3.86 (2H, m, CH_2_), 4.02–4.10 (2H, m, CH_2_), 5.45 (1H, s, CH), 7.28 (2H, d, *J* = 8.4 Hz, ArH), 7.36 (2H, d, *J* = 8.4 Hz, ArH), 7.96 (2H, d, *J* = 8.7 Hz, ArH), 8.20 (2H, d, *J* = 8.7 Hz, ArH), 8.21 (2H, s, NH_2_), 9.16 (1H, s, NH), 9.43 (1H, s, NH); ^13^C{^1^H} NMR (75.4 MHz, DMSO-*d*_6_): *δ* 13.9 (CH_3_), 37.8 (CH), 43.6 (CH_2_–NH), 44.7 (CH_2_–N), 80.2 (C=**C**–CO), 107.7 (C=**C**–NO_2_), 123.9, 127.3, 128.5, 129.8, 130.2, 131.3, 145.0 (Ar), 147.5 (Me–**C**=N), 151.3 (C=**C**–NH), 151.4 (C_Ar_–NO_2_), 151.8 (C=**C**–NH_2_), 165.7 (C=O); anal. calcd for C_22_H_20_ClN_7_O_5_: C, 53.07; H, 4.05; N, 19.69. Found: C, 53.28; H, 4.16; N, 19.51.

#### 5-Amino-7-(4-nitrophenyl)-8-nitro-*N*′-(1-(4-nitrophenyl)ethylidene)-1,2,3,7-tetrahydroimidazo[1,2-*a*]pyridine-6-carbohydrazide (6d)

Light Yellow solid; yield: 0.452 g (89%); mp: 243–245 °C; ^1^H NMR (300 MHz, DMSO-*d*_6_): *δ* 2.18 (3H, s, CH_3_), 3.79–3.85 (2H, m, CH_2_), 4.04–4.07 (2H, m, CH_2_), 5.67 (1H, s, CH), 7.59 (2H, d, *J* = 9 Hz, ArH), 7.95 (2H, d, *J* = 9 Hz, ArH), 8.10 (2H, d, *J* = 8.7 Hz, ArH), 8.20 (2H, d, *J* = 8.7 Hz, ArH), 8.27 (2H, s, NH_2_), 9.34 (1H, s, NH), 9.50 (1H, s, NH); ^13^C{^1^H} NMR (75.4 MHz, DMSO-*d*_6_): *δ* 14.1 (CH_3_), 38.3 (CH), 43.7 (CH_2_–NH), 44.7 (CH_2_–N), 79.7 (C=**C**–CO), 107.1 (C=**C**–NO_2_), 123.6, 123.9, 124.4, 127.3, 129.6, 145.0, 146.4 (Ar), 147.5 (Me–**C**=N), 147.6 (C=**C**–NH), 151.4 (C_Ar_–NO_2_), 151.5 (C_Ar_–NO_2_), 153.4 (C=**C**–NH_2_), 166.0 (C=O); MS (EI, 70 eV): *m*/*z* (%) = 508 (0.01) [M]^+^, 326 (40), 311 (72), 257 (32), 204 (14), 179 (100), 117 (59), 76 (7), 57 (12); anal. calcd for C_22_H_20_N_8_O_7_: C, 51.97; H, 3.96; N, 22.04. Found: C, 52.13; H, 3.89; N, 22.14.

#### 5-Amino-7-(4-bromophenyl)-8-nitro-*N*′-(1-(4-nitrophenyl)ethylidene)-1,2,3,7-tetrahydroimidazo[1,2-*a*]pyridine-6-carbohydrazide (6e)

Yellow solid; yield: 0.486 g (90%); mp: 239–240 °C; IR (KBr) (*ν*_max_/cm^−1^): 3454, 3338, 2919, 1658, 1522, 1462, 1377, 1254, 580; ^1^H NMR (300 MHz, DMSO-*d*_6_): *δ* 2.14 (3H, s, CH_3_), 3.79–3.85 (2H, m, CH_2_), 4.00–4.07 (2H, m, CH_2_), 5.42 (1H, s, CH), 7.30 (2H, m, ArH), 7.39 (2H, m, ArH), 7.94 (2H, d, *J* = 7.5 Hz, ArH), 8.19 (2H, d, *J* = 7.5 Hz, ArH), 8.20 (2H, s, NH_2_), 9.16 (1H, s, NH), 9.43 (1H, s, NH); ^13^C{^1^H} NMR (75.4 MHz, DMSO-*d*_6_): *δ* 13.9 (CH_3_), 37.9 (CH), 43.6 (CH_2_–NH), 44.7 (CH_2_–N), 80.1 (C=**C**–CO), 107.6 (C=**C**–NO_2_), 119.8, 123.8, 127.3, 130.2, 130.6, 131.1, 144.9 (Ar) 147.1 (Me–**C**=N), 147.4 (C=**C**–NH), 151.4 (C_Ar_–NO_2_), 151.4 (C=**C**–NH_2_), 165.7 (C=O); anal. calcd for C_22_H_20_BrN_7_O_5_: C, 48.72; H, 3.72; N, 18.08. Found: C, 48.64; H, 3.78; N, 18.19.

#### 5-Amino-7-(4-methoxyphenyl)-8-nitro-*N*′-(1-(4-nitrophenyl)ethylidene)-1,2,3,7-tetrahydroimidazo[1,2-*a*]pyridine-6-carbohydrazide (6f)

Dark yellow solid; yield: 0.342 g (78%); mp: 267–269 °C; IR (KBr) (*ν*_max_/cm^−1^): 3401, 3343, 2919, 2850, 1658, 1514, 1451, 1342, 1257, 1167; ^1^H NMR (300 MHz, DMSO-*d*_6_): *δ* 2.11 (3H, s, CH_3_), 3.69 (3H, s, OCH_3_), 3.74–3.83 (2H, m, CH_2_), 4.02–4.05 (2H, m, CH_2_), 5.28 (1H, s, CH), 6.79 (2H, d, *J* = 8.4 Hz, ArH), 7.27 (2H, d, *J* = 8.4 Hz, ArH), 7.94 (2H, d, *J* = 8.7 Hz, ArH), 8.19 (2H, d, *J* = 8.7 Hz, ArH), 8.20 (2H, br s, NH_2_), 8.97 (1H, s, NH), 9.38 (1H, s, NH); ^13^C{^1^H} NMR (75.4 MHz, DMSO-*d*_6_): *δ* 13.7 (CH_3_), 37.7 (CH), 43.5 (CH_2_–NH), 44.7 (CH_2_–N), 55.4 (OCH_3_), 80.6 (C=**C**–CO), 108.2 (C=**C**–NO_2_), 113.8, 123.9, 127.3, 129.4, 137.4, 145.0 (Ar), 146.4 (Me–**C**=N), 147.4 (C=**C**–NH), 151.2 (C_Ar_–NO_2_), 151.4 (C=**C**–NH_2_), 158.3 (**C**_Ar_–OCH_3_), 165.5 (C=O); anal. calcd for C_23_H_23_N_7_O_6_: C, 55.98; H, 4.70; N, 19.87. Found: C, 55.93; H, 4.77; N, 19.75.

#### 5-Amino-7-(4-fluorophenyl)-8-nitro-*N*′-(1-(4-nitrophenyl)ethylidene)-1,2,3,7-tetrahydroimidazo[1,2-*a*]pyridine-6-carbohydrazide (6g)

Light orange solid; yield: 0.384 g (80%); mp: 271–273 °C; ^1^H NMR (300 MHz, DMSO-*d*_6_): *δ* 2.12 (3H, s, CH_3_), 3.80–3.83 (2H, m, CH_2_), 4.03–4.05 (2H, m, CH_2_), 5.42 (1H, s, CH), 6.96–7.23 (4H, m, ArH), 7.95 (2H, d, *J* = 9 Hz, ArH), 8.19 (2H, d, *J* = 9 Hz, ArH), 8.24 (2H, br s, NH_2_), 9.10 (1H, s, NH), 9.42 (1H, s, NH); ^13^C{^1^H} NMR (75.4 MHz, DMSO-*d*_6_): *δ* 13.8 (CH_3_), 37.6 (CH), 43.5 (CH_2_–NH), 44.7 (CH_2_–N), 80.4 (C=**C**–CO), 107.9 (C=**C**–NO_2_), 114.8, 116.0, 123.9, 127.3, 127.9, 128.8, 130.1 (Ar), 141.7 (Me–**C**=N), 142.4 (C=**C**–NH), 147.4 (C_Ar_–NO_2_), 151.4 (C=**C**–NH_2_), 165.6 (C=O); anal. calcd for C_22_H_20_FN_7_O_5_: C, 54.88; H, 4.19; N, 20.37. Found: C, 54.55; H, 3.94; N, 20.15.

#### 5-Amino-8-nitro-*N*′-(1-(4-nitrophenyl)ethylidene)-7-phenyl-1,2,3,7-tetrahydroimidazo[1,2-*a*]pyridine-6-carbohydrazide (6h)

Yellow solid; yield: 0.347 g (75%); mp: 284–286 °C; ^1^H NMR (300 MHz, DMSO-*d*_6_): *δ* 2.12 (3H, s, CH_3_), 3.81–3.84 (2H, m, CH_2_), 4.05–4.08 (2H, m, CH_2_), 5.36 (1H, s, CH), 7.12–7.36 (5H, m, ArH), 7.94 (2H, d, *J* = 7.8 Hz, ArH), 8.19 (2H, d, *J* = 7.8 Hz, ArH), 8.19 (2H, br s, NH_2_), 9.05 (1H, s, NH), 9.40 (1H, s, NH); ^13^C{^1^H} NMR (75.4 MHz, DMSO-*d*_6_): *δ* 13.8 (CH_3_), 38.4 (CH), 43.6 (CH_2_–NH), 44.7 (CH_2_–N), 80.6 (C=**C**–CO), 107.4 (C=**C**–NO_2_), 123.9, 126.9, 127.3, 128.4, 145.5 (Ar), 146.6 (Me–**C**=N), 147.5 (C=**C**–NH), 151.3 (C_Ar_–NO_2_), 151.5 (C=**C**–NH_2_), 165.7 (C=O); anal. calcd for C_22_H_21_N_7_O_5_: C, 57.02; H, 4.57; N, 21.16. Found: C, 57.37; H, 4.25; N, 21.45.

#### 6-Amino-8-(4-bromophenyl)-9-nitro-*N*′-(1-(4-nitrophenyl)ethylidene)-2,3,4,8-tetrahydro-1*H*-pyrido[1,2-*a*]pyrimidine-7-carbohydrazide (6i)

Light yellow solid; yield: 0.477 g (86%); mp: 264–266 °C; IR (KBr) (*ν*_max_/cm^−1^): 3481, 3403, 3968, 2925, 1645, 1516, 1456, 1340, 1257, 590; ^1^H NMR (300 MHz, DMSO-*d*_6_): *δ* 1.80–1.92 (2H, m, CH_2_), 2.48 (3H, s, CH_3_), 3.58–3.75 (2H, m, CH_2_NH), 3.76–3.92 (2H, m, CH_2_N), 5.53 (1H, s, CH), 7.26 (2H, d, *J* = 8.4 Hz, ArH), 7.41 (2H, d, *J* = 8.4 Hz, ArH), 7.97 (2H, d, *J* = 8.7 Hz, ArH), 8.20 (2H, d, *J* = 8.7 Hz, ArH), 8.25 (2H, s, NH_2_), 9.36 (1H, s, NH), 11.49 (1H, s, NH); ^13^C{^1^H} NMR (75.4 MHz, DMSO-*d*_6_): *δ* 14.0 (CH_3_), 20.2 (CH_2_), 36.6 (CH), 38.7 (CH_2_–NH), 43.1 (CH_2_–N), 81.4 (C=**C**–CO), 109.8 (C=**C**–NO_2_), 119.9, 123.9, 127.4, 130.4, 131.4, 144.1, 144.9 (Ar), 147.6 (Me–**C**=N), 147.9 (C=**C**–NH), 150.3 (C_Ar_–NO_2_), 152.6 (C=**C**–NH_2_), 166.1 (C=O); MS (EI, 70 eV): *m*/*z* (%) = 343 (58), 311 (53), 265 (20), 243 (8), 204 (13), 183 (40), 156 (27), 133 (10), 117 (100), 97 (25), 76 (8), 57 (55); anal. calcd for C_23_H_22_BrN_7_O_5_: C, 49.65; H, 3.99; N, 17.62. Found: C, 49.87; H, 3.50; N, 17.26.

#### 6-Amino-9-nitro-8-(4-nitrophenyl)-*N*′-(1-(4-nitrophenyl)ethylidene)-2,3,4,8-tetrahydro-1*H*-pyrido[1,2-*a*]pyrimidine-7-carbohydrazide (6j)

Yellowish orange solid; yield: 0.459 g (88%); mp: 268–271 °C; IR (KBr) (*ν*_max_/cm^−1^): 3470, 3389, 2929, 1648, 1521, 1458, 1344, 1259; ^1^H NMR (300 MHz, DMSO-*d*_6_): *δ* 1.85–1.94 (2H, m, CH_2_), 2.48 (3H, s, CH_3_), 3.58–3.75 (2H, m, CH_2_NH), 3.76–3.95 (2H, m, CH_2_N), 5.75 (1H, s, CH), 7.55 (2H, d, *J* = 8.4 Hz, ArH), 7.97 (2H, d, *J* = 8.4 Hz, ArH), 8.09 (2H, d, *J* = 8.7 Hz, ArH), 8.19 (2H, d, *J* = 8.7 Hz, ArH), 8.31 (2H, s, NH_2_), 9.55 (1H, s, NH), 11.47 (1H, s, NH); ^13^C{^1^H} NMR (75.4 MHz, DMSO-*d*_6_): *δ* 14.2 (CH_3_), 20.1 (CH_2_), 37.15 (CH), 38.8 (CH_2_–NH), 43.2 (CH_2_–N), 81.0 (C=**C**–CO), 109.3 (C=**C**–NO_2_), 123.8, 127.4, 128.4, 129.1, 144.9, 146.4 (Ar), 147.6 (Me–**C**=N), 148.4 (C=**C**–NH), 150.4 (C_Ar_–NO_2_), 152.5 (C_Ar_–NO_2_), 152.8 (C=**C**–NH_2_), 166.3 (C=O); MS (EI, 70 eV): *m*/*z* (%) = 353 (6), 311 (35), 271 (67), 225 (19), 179 (100), 164 (67), 132 (49), 118 (77), 91 (35), 76 (13), 56 (31); anal. calcd for C_23_H_22_N_8_O_7_: C, 52.87; H, 4.24; N, 21.45. Found: C, 52.57; H, 4.62; N, 21.18.

#### 6-Amino-8-(4-chlorophenyl)-9-nitro-*N*′-(1-(4-nitrophenyl)ethylidene)-2,3,4,8-tetrahydro-1*H*-pyrido[1,2-*a*]pyrimidine-7-carbohydrazide (6k)

Light brown solid; yield: 0.434 g (85%); mp: 265–267 °C; ^1^H NMR (300 MHz, DMSO-*d*_6_): *δ* 1.80–1.90 (2H, m, CH_2_), 2.17 (3H, s, CH_3_), 3.38–3.92 (4H, m, 2CH_2_N), 5.54 (1H, s, CH), 7.27 (2H, d, *J* = 8.7 Hz, ArH), 7.32 (2H, d, *J* = 8.7 Hz, ArH), 7.97 (2H, d, *J* = 8.7 Hz, ArH), 8.20 (2H, d, *J* = 8.7 Hz, ArH), 8.25 (2H, s, NH_2_), 9.37 (1H, s, NH), 11.49 (1H, s, NH); ^13^C{^1^H} NMR (75.4 MHz, DMSO-*d*_6_): *δ* 14.0 (CH_3_), 20.2 (CH_2_), 36.5 (CH), 38.7 (CH_2_–NH), 43.1 (CH_2_–N), 81.5 (C=**C**–CO), 109.8 (C=**C**–NO_2_), 123.9, 127.4, 128.5, 129.8, 131.4, 143.7, 144.9 (Ar), 147.6 (Me–**C**=N), 147.9 (C=**C**–NH), 150.3 (C_Ar_–NO_2_), 152.6 (C=**C**–NH_2_), 166.1 (C=O); anal. calcd for C_23_H_22_ClN_7_O_5_: C, 53.96; H, 4.33; N, 19.15. Found: C, 53.61; H, 4.14; N, 19.37.

#### 6-Amino-3,3-dimethyl-9-nitro-8-(4-nitrophenyl)-*N*′-(1-(4-nitrophenyl)ethylidene)-2,3,4,8-tetrahydro-1*H*-pyrido[1,2-*a*]pyrimidine-7-carbohydrazide (6l)

Creamy yellow solid; yield: 0.495 g (90%); mp: 297–300 °C; IR (KBr) (*ν*_max_/cm^−1^): 3320, 3199, 2959, 1642, 1518, 1350, 1261; ^1^H NMR (300 MHz, DMSO-*d*_6_): *δ* 0.95 (3H, s, CH_3_), 1.09 (3H, s, CH_3_), 2.19 (3H, s, CH_3_), 3.23–3.32 (2H, m, CH_2_NH), 3.52–3.56 (2H, m, CH_2_N), 5.80 (1H, s, CH), 7.52 (2H, d, *J* = 7.5 Hz, ArH), 7.96 (2H, d, *J* = 7.5 Hz, ArH), 8.11 (2H, d, *J* = 7.8 Hz, ArH), 8.19 (2H, d, *J* = 7.8 Hz, ArH), 8.33 (2H, s, NH_2_), 9.54 (1H, s, NH), 11.38 (1H, s, NH); ^13^C{^1^H} NMR (75.4 MHz, DMSO-*d*_6_): *δ* 14.2 (CH_3_), 22.7 (CH_3_), 24.5 (CH_3_), 27.7 (CMe_2_), 37.0 (CH), 49.4 (CH_2_–NH), 53.4 (CH_2_–N), 81.0 (C=**C**–CO), 109.2 (C=**C**–NO_2_), 123.9, 127.5, 128.9, 144.9, 146.5 (Ar), 147.6 (Me–**C**=N), 148.5 (C=**C**–NH), 149.6 (C_Ar_–NO_2_), 152.6 (C_Ar_–NO_2_), 152.9 (C=**C**–NH_2_), 166.3 (C=O); MS (EI, 70 eV): *m*/*z* (%) = 409 (6), 345 (13), 327 (38), 299 (84), 253 (20), 179 (100), 149 (52), 118 (71), 96 (14), 77 (78), 55 (42); anal. calcd for C_25_H_26_N_8_O_7_: C, 54.54; H, 4.76; N, 20.35. Found: C, 54.68; H, 4.59; N, 20.24.

#### 6-Amino-8-(3-methoxyphenyl)-3,3-dimethyl-9-nitro-*N*′-(1-(4-nitrophenyl)ethylidene)-2,3,4,8-tetrahydro-1*H*-pyrido[1,2-*a*]pyrimidine-7-carbohydrazide (6m)

Dark yellow solid; yield: 0.390 g (73%); mp: 269–271 °C; ^1^H NMR (300 MHz, DMSO-*d*_6_): *δ* 0.96 (3H, s, CH_3_), 1.09 (3H, s, CH_3_), 2.14 (3H, s, CH_3_), 3.23 (2H, s, CH_2_NH), 3.56 (2H, s, CH_2_N), 3.65 (3H, s, OCH_3_), 5.49 (1H, s, CH), 6.71–6.89 (3H, m, ArH), 7.15 (1H, t, *J* = 7.5 Hz, ArH), 7.97 (2H, d, *J* = 9 Hz, ArH), 8.20 (2H, d, *J* = 9 Hz, ArH), 8.25 (2H, s, NH_2_), 9.23 (1H, s, NH), 11.44 (1H, s, NH); ^13^C{^1^H} NMR (75.4 MHz, DMSO-*d*_6_): *δ* 13.8 (CH_3_), 22.6 (CH_3_), 24.6 (CH_3_), 27.6 (CMe_2_), 36.2 (CH), 49.4 (CH_2_–NH), 53.3 (CH_2_–N), 55.4 (OCH_3_), 82.0 (C=**C**–CO), 110.2 (C=**C**–NO_2_), 114.0, 115.3, 123.9, 127.4, 128.0, 133.2, 136.5, 144.9 (Ar), 147.3 (Me–**C**=N), 147.5 (C=**C**–NH), 149.4 (C_Ar_–NO_2_), 152.6 (C=**C**–NH_2_), 158.4 (C_Ar_–OCH_3_), 165.8 (C=O); anal. calcd for C_25_H_29_N_7_O_6_: C, 58.31; H, 5.46; N, 18.31. Found: C, 58.50; H, 5.34; N, 18.27.

#### 6-Amino-8-(4-methoxyphenyl)-3,3-dimethyl-9-nitro-*N*′-(1-(4-nitrophenyl)ethylidene)-2,3,4,8-tetrahydro-1*H*-pyrido[1,2-*a*]pyrimidine-7-carbohydrazide (6n)

Yellow solid; yield: 0.401 g (75%); mp: 315–317 °C; ^1^H NMR (300 MHz, DMSO-*d*_6_): *δ* 0.96 (3H, s, CH_3_), 1.09 (3H, s, CH_3_), 2.13 (3H, s, CH_3_), 3.26–3.33 (2H, m, CH_2_NH), 3.55–3.65 (2H, s, CH_2_N), 3.75 (3H, s, OCH_3_), 5.41 (1H, s, CH), 6.79 (2H, d, *J* = 8.7 Hz, ArH), 7.23 (2H, d, *J* = 8.7 Hz, ArH), 7.96 (2H, d, *J* = 9 Hz, ArH), 8.20 (2H, d, *J* = 9 Hz, ArH), 8.24 (2H, s, NH_2_), 9.62 (1H, s, NH), 11.45 (1H, s, NH); ^13^C{^1^H} NMR (75.4 MHz, DMSO-*d*_6_): *δ* 13.8 (CH_3_), 22.6 (CH_3_), 24.1 (CH_3_), 27.7 (CMe_2_), 36.3 (CH), 49.2 (CH_2_–NH), 53.3 (CH_2_–N), 55.4 (OCH_3_), 82.0 (C=**C**–CO), 110.2 (C=**C**–NO_2_), 114.0, 115.3, 123.9, 127.4, 128.9, 133.2, 136.5, 144.9 (Ar), 147.3 (Me–**C**=N), 147.5 (C=**C**–NH), 149.4 (C_Ar_–NO_2_), 152.6 (C=**C**–NH_2_), 158.4 (C_Ar_–OCH_3_), 165.8 (C=O); anal. calcd for C_25_H_29_N_7_O_6_: C, 58.31; H, 5.46; N, 18.31. Found: C, 58.68; H, 5.15; N, 18.23.

#### 6-Amino-8-(4-chlorophenyl)-3,3-dimethyl-9-nitro-*N*′-(1-(4-nitrophenyl)ethylidene)-2,3,4,8-tetrahydro-1*H*-pyrido[1,2-*a*]pyrimidine-7-carbohydrazide (6o)

Brownish yellow solid; yield: 0.458 g (85%); mp: 317–319 °C; IR (KBr) (*ν*_max_/cm^−1^): 3324, 3204, 2925, 2861, 1643, 1524, 1480, 1353, 1263, 755; ^1^H NMR (300 MHz, DMSO-*d*_6_): *δ* 0.93 (3H, s, CH_3_), 1.08 (3H, s, CH_3_), 2.16 (3H, s, CH_3_), 3.23 (2H, s, CH_2_NH), 3.54 (2H, m, CH_2_N), 5.58 (1H, s, CH), 7.29 (4H, s, ArH), 7.96 (2H, d, *J* = 8.7 Hz, ArH), 8.20 (2H, d, *J* = 8.7 Hz, ArH), 8.27 (2H, s, NH_2_), 9.36 (1H, s, NH), 11.40 (1H, s, NH); ^13^C{^1^H} NMR (75.4 MHz, DMSO-*d*_6_): *δ* 14.0 (CH_3_), 22.6 (CH_3_), 24.5 (CH_3_), 27.7 (CMe_2_), 37.0 (CH), 50.4 (CH_2_–NH), 51.5 (CH_2_–N), 81.5 (C=**C**–CO), 109.7 (C=**C**–NO_2_), 123.9, 124.0, 127.5, 128.0, 128.5, 128.8, 129.1, 129.6 (Ar), 143.7 (Me–**C**=N), 144.6 (C=**C**–NH), 148.0 (C_Ar_–NO_2_), 152.9 (C=**C**–NH_2_), 166.0 (C=O); MS (EI, 70 eV): *m*/*z* (%) = 539 (0.007) [M]^+^, 368 (4), 311 (30), 288 (100), 223 (13), 179 (57), 149 (27), 117 (37), 102 (20), 77 (42), 55 (27); anal. calcd for C_25_H_26_ClN_7_O_5_: C, 55.61; H, 4.85; N, 18.16. Found: C, 55.90; H, 4.68; N, 18.04.

#### 6-Amino-8-(4-bromophenyl)-3,3-dimethyl-9-nitro-*N*′-(1-(4-nitrophenyl)ethylidene)-2,3,4,8-tetrahydro-1*H*-pyrido[1,2-*a*]pyrimidine-7-carbohydrazide (6p)

Yellow solid; yield: 0.491 g (84%); mp: 322–324 °C; ^1^H NMR (300 MHz, DMSO-*d*_6_): *δ* 1.03 (3H, s, CH_3_), 1.08 (3H, s, CH_3_), 2.16 (3H, s, CH_3_), 3.22–3.35 (2H, m, CH_2_NH), 3.42–3.55 (2H, m, CH_2_N), 5.56 (1H, s, CH), 7.24 (2H, d, *J* = 8.4 Hz, ArH), 7.43 (2H, d, *J* = 8.4 Hz, ArH), 7.96 (2H, d, *J* = 9 Hz, ArH), 8.20 (2H, d, *J* = 9 Hz, ArH), 8.29 (2H, s, NH_2_), 9.38 (1H, s, NH), 11.41 (1H, s, NH); ^13^C{^1^H} NMR (75.4 MHz, DMSO-*d*_6_): *δ* 14.0 (CH_3_), 22.6 (CH_3_), 24.5 (CH_3_), 27.6 (CMe_2_), 36.4 (CH), 49.4 (CH_2_–NH), 53.4 (CH_2_–N), 81.4 (C=**C**–CO), 109.7 (C=**C**–NO_2_), 120.0, 123.9, 127.5, 128.0, 130.0, 131.4, 132.0 (Ar), 144.1 (Me–**C**=N), 147.6 (C=**C**–NH), 149.5 (C_Ar_–NO_2_), 152.8 (C=**C**–NH_2_), 166.1 (C=O); anal. calcd for C_25_H_26_BrN_7_O_5_: C, 51.38; H, 4.48; N, 16.78. Found: C, 51.18; H, 4.66; N, 16.47.

#### 6-Amino-3,3-dimethyl-9-nitro-*N*′-(1-(4-nitrophenyl)ethylidene)-8-phenyl-2,3,4,8-tetrahydro-1*H*-pyrido[1,2-*a*]pyrimidine-7-carbohydrazide (6q)

Light yellow solid; yield: 0.378 g (75%); mp: 329–331 °C; ^1^H NMR (300 MHz, DMSO-*d*_6_): *δ* 0.95 (3H, s, CH_3_), 1.09 (3H, s, CH_3_), 2.14 (3H, s, CH_3_), 3.19–3.26 (2H, m, CH_2_NH), 3.56 (2H, s, CH_2_N), 5.50 (1H, s, CH), 7.11–7.32 (5H, m, ArH), 7.96 (2H, d, *J* = 8.7 Hz, ArH), 8.20 (2H, d, *J* = 8.7 Hz, ArH), 8.26 (2H, s, NH_2_), 9.26 (1H, s, NH), 11.44 (1H, s, NH); ^13^C{^1^H} NMR (75.4 MHz, DMSO-*d*_6_): *δ* 13.9 (CH_3_), 22.6 (CH_3_), 24.6 (CH_3_), 27.7 (CMe_2_), 37.0 (CH), 49.4 (CH_2_–NH), 53.3 (CH_2_–N), 81.9 (C=**C**–CO), 110.0 (C=**C**–NO_2_), 120.0, 123.9, 127.5, 128.0, 130.0, 131.4, 132.0 (Ar), 144.1 (Me–**C**=N), 147.5 (C=**C**–NH), 150.5 (C_Ar_–NO_2_), 152.8 (C=**C**–NH_2_), 166.0 (C=O); MS (EI, 70 eV): *m*/*z* (%) = 505 (0.03) [M]^+^, 447 (0.09), 334 (14), 300 (25), 283 (19), 254 (100), 179 (20), 156 (89), 128 (41), 103 (26), 77 (42), 55 (11); anal. calcd for C_25_H_27_N_7_O_5_: C, 59.40; H, 5.38; N, 19.40. Found: C, 59.56; H, 5.25; N, 19.34.

## Conclusion

A green and convenient approach to easy construction of novel and highly substituted imidazo[1,2-*a*]pyridines and pyrido[1,2-*a*]pyrimidines has been developed by annulation of heterocyclic ketene aminals (HKAs) and a three-component product of cyanoacetohydrazide, 4-nitroacetophenone and aromatic aldehydes. The reactions are performed in a mixture of EtOH and H_2_O at reflux conditions in the absence of any catalyst. The present reaction shows considerable properties such as high regioselectivity, cascade one-pot methodology, short reaction times, easy purification of products, high yields, avoiding the use of catalyst and high atom economy.

## Conflicts of interest

The authors declare no competing financial interest.

## Supplementary Material

RA-009-C9RA00350A-s001
