# Supplementary material for: An efficient synthesis of new imidazo[1,2-a]pyridine-6-carbohydrazide and pyrido[1,2-a]pyrimidine-7-carbohydrazide derivatives via a five-component cascade reaction
Source: RSC Adv. 2019 Mar 5;9(13):7218–27. doi: 10.1039/c9ra00350a (PMC9061117; doi:10.1039/c9ra00350a)
Supplement: RA-009-C9RA00350A-s001 [file RA-009-C9RA00350A-s001.pdf]

Electronic Supplementary Material (ESI) for RSC Advances.
This journal is © The Royal Society of Chemistry 2019

Supporting Information

An efficient synthesis of new imidazo[1,2-*a*]pyridine-6-carbohydrazide and pyrido[1,2-*a*]pyrimidine-7-carbohydrazide derivatives *via* a five-component cascade reaction

Hajar Hosseini and Mohammad Bayat

Department of Chemistry, Faculty of Science, Imam Khomeini International University, Qazvin, Iran. E-mail: bayat_mo@yahoo.com, m.bayat@sci.ikiu.ac.ir

The Table of Contents

Title	Page
Title, author's name, address and table of contents	1-2
Experimental Section; General remarks	2
**Figure 1.** Structure of all products **6a-q**	3
^{1}H and ^{13}C NMR and IR and Mass spectrums of **6a**	5-8
^{1}H and ^{13}C NMR and IR and Mass spectrums of **6b**	9-12
^{1}H and ^{13}C NMR and IR spectrums of **6c**	13-15
^{1}H and ^{13}C NMR and Mass spectrums of **6d**	16-18
^{1}H and ^{13}C NMR and IR spectrums of **6e**	19-21
^{1}H and ^{13}C NMR and IR spectrums of **6f**	22-24
^{1}H and ^{13}C NMR spectrums of **6g**	25-26
^{1}H and ^{13}C NMR spectrums of **6h**	27-28
^{1}H and ^{13}C NMR and IR and Mass spectrums of **6i**	29-32
^{1}H and ^{13}C NMR and IR and Mass spectrums of **6j**	33-36
^{1}H and ^{13}C NMR spectrums of **6k**	37-38
^{1}H and ^{13}C NMR and IR and Mass spectrums of **6l**	39-42
^{1}H and ^{13}C NMR spectrums of **6m**	43-44
^{1}H and ^{13}C NMR spectrums of **6n**	45-46

^{1}H and ^{13}C NMR and IR and Mass spectrums of **6o**	47-50
^{1}H and ^{13}C NMR spectrums of **6p**	51-52
^{1}H and ^{13}C NMR and Mass spectrums of **6q**	53-55

Experimental Section

General remarks:

Melting points were measured on an Electrothermal 9100 apparatus. Mass spectra were recorded with an Agilent 5975C VL MSD with Triple-Axis Detector operating at an ionization potential of 70 eV. ^{1}H and ^{13}C NMR spectra were measured (DMSO) with a Bruker DRX-300 AVANCE spectrometer at 300 and 75 MHz, respectively. IR spectra were recorded on a Bruker Tensor 27, ʋ̄ in cm^{-1}. All NMR spectra at room temperature were determined in DMSO-d_6. Chemical shifts are reported in parts per million (*δ*) downfield from an internal tetramethylsilane reference. Coupling constants (*J* values) are reported in hertz (Hz), and spin multiplicities are indicated by the following symbols: s (singlet), d (doublet), t (triplet), q (quartet), m (multiplet). All chemicals were purchased from Merck or Aldrich and were used without further purification.

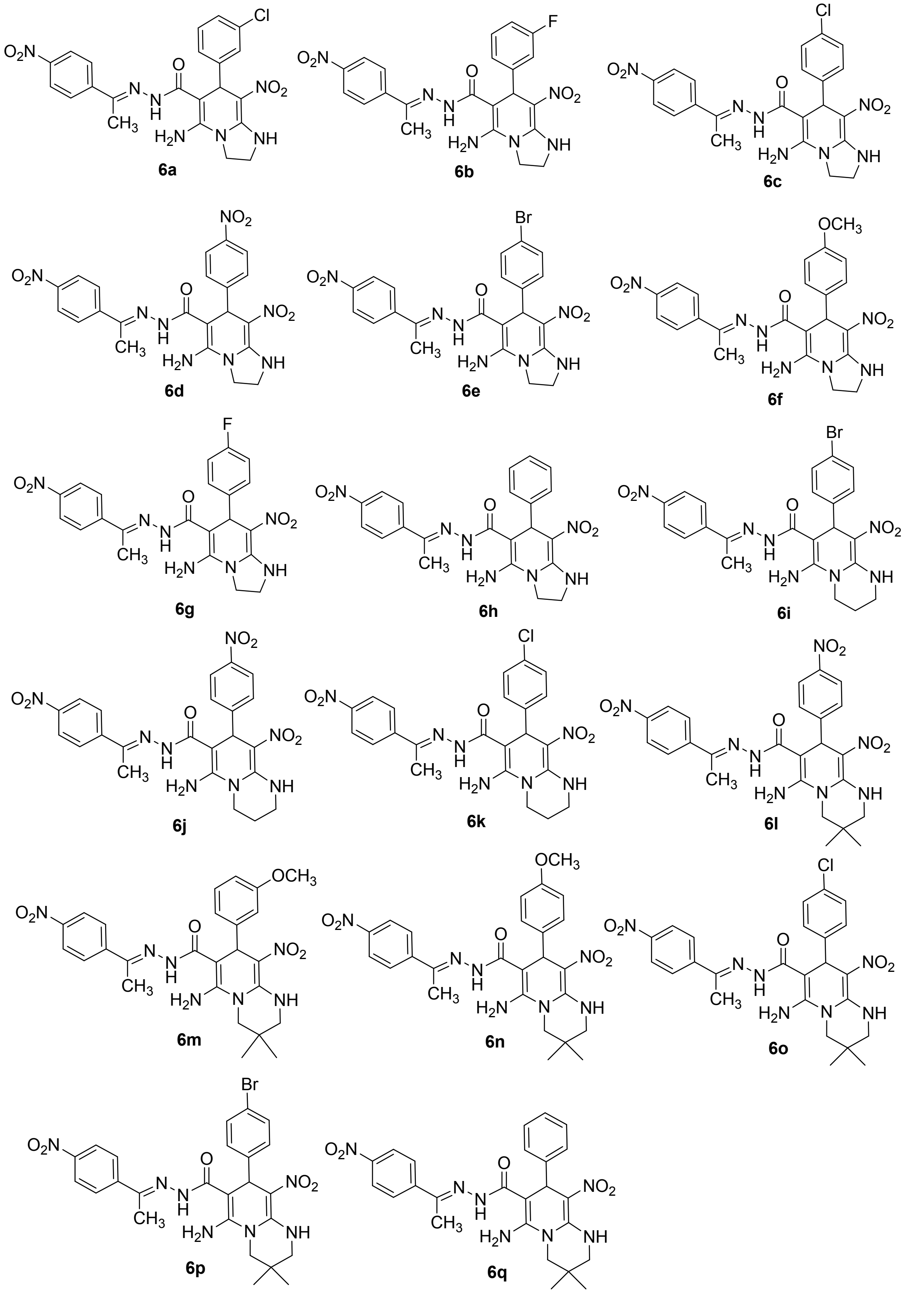

Figure 1. Structure of all products **6a-q.**

The structures of all products **6a-q** were deduced from their IR, mass, ^{1}H NMR, and ^{13}C NMR spectra (see the following images).

The ^{1}H and ^{13}C NMR spectra are taken in DMSO-d_6, but some of the products are slightly soluble in the solvent therefore have no clear carbon spectra such as **6g, 6h, 6m, 6o, 6p, 6q.**

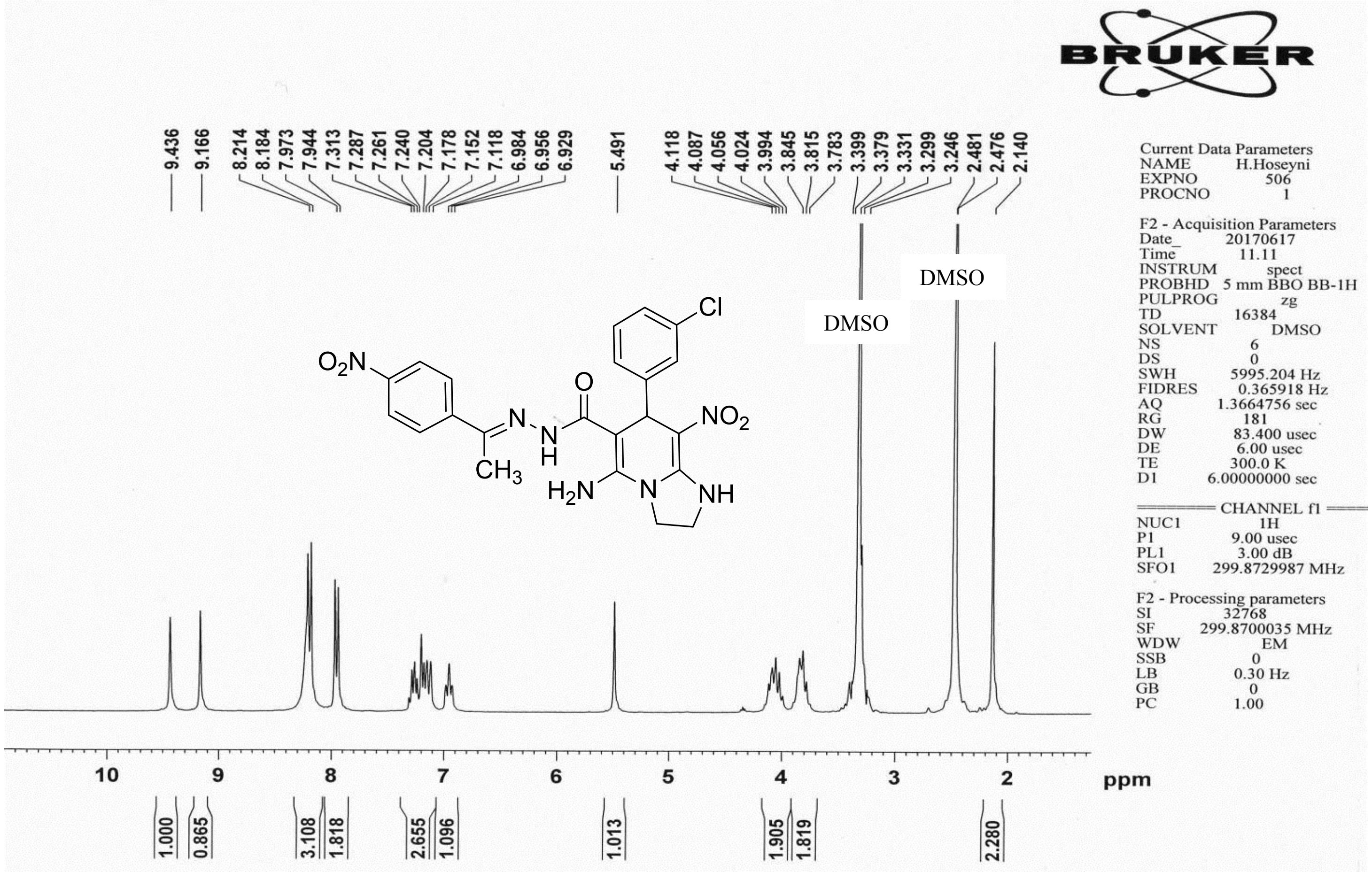
BRUKER
9.436
9.166
8.214
8.184
7.973
7.944
7.313
7.287
7.261
7.240
7.204
7.178
7.152
7.118
6.984
6.956
6.929
5.491
4.118
4.087
4.056
4.024
3.994
3.845
3.815
3.783
3.399
3.379
3.331
3.299
3.246
2.481
2.476
2.140
DMSO
DMSO
Cl
O_2N
O
N
N
H
NO_2
CH_3
H_2N
N
NH
Current Data Parameters
NAME H.Hoseyni
EXPNO 506
PROCNO 1
F2 - Acquisition Parameters
Date_ 20170617
Time 11.11
INSTRUM spect
PROBHD 5 mm BBO BB-1H
PULPROG zg
TD 16384
SOLVENT DMSO
NS 6
DS 0
SWH 5995.204 Hz
FIDRES 0.365918 Hz
AQ 1.3664756 sec
RG 181
DW 83.400 usec
DE 6.00 usec
TE 300.0 K
D1 6.00000000 sec
CHANNEL f1
NUC1 1H
P1 9.00 usec
PL1 3.00 dB
SFO1 299.8729987 MHz
F2 - Processing parameters
SI 32768
SF 299.8700035 MHz
WDW EM
SSB 0
LB 0.30 Hz
GB 0
PC 1.00
10
9
8
7
6
5
4
3
2
ppm
1.000
0.865
3.108
1.818
2.655
1.096
1.013
1.905
1.819
2.280

^{1}H NMR of 6a

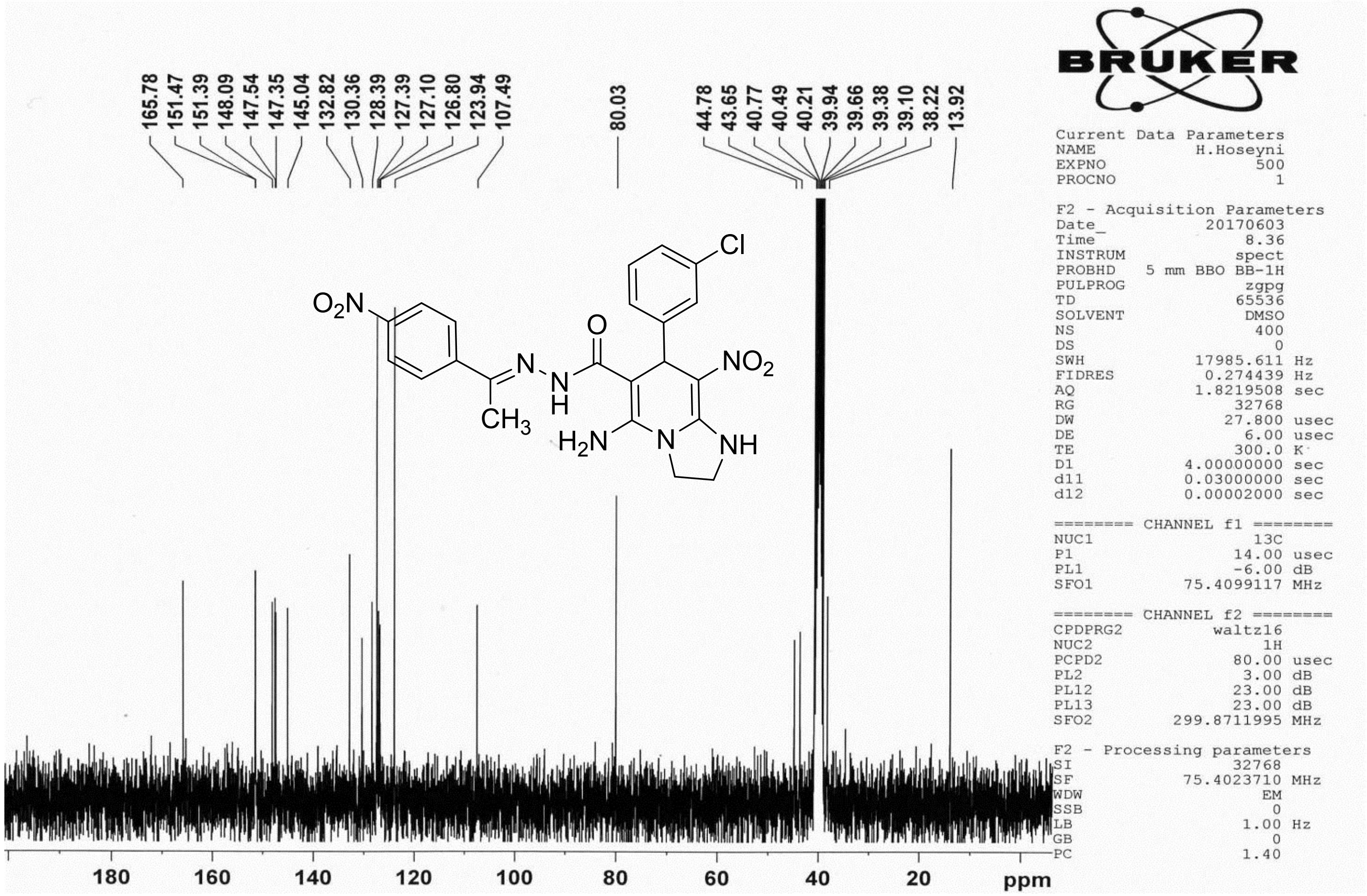

^{13}C NMR of 6a

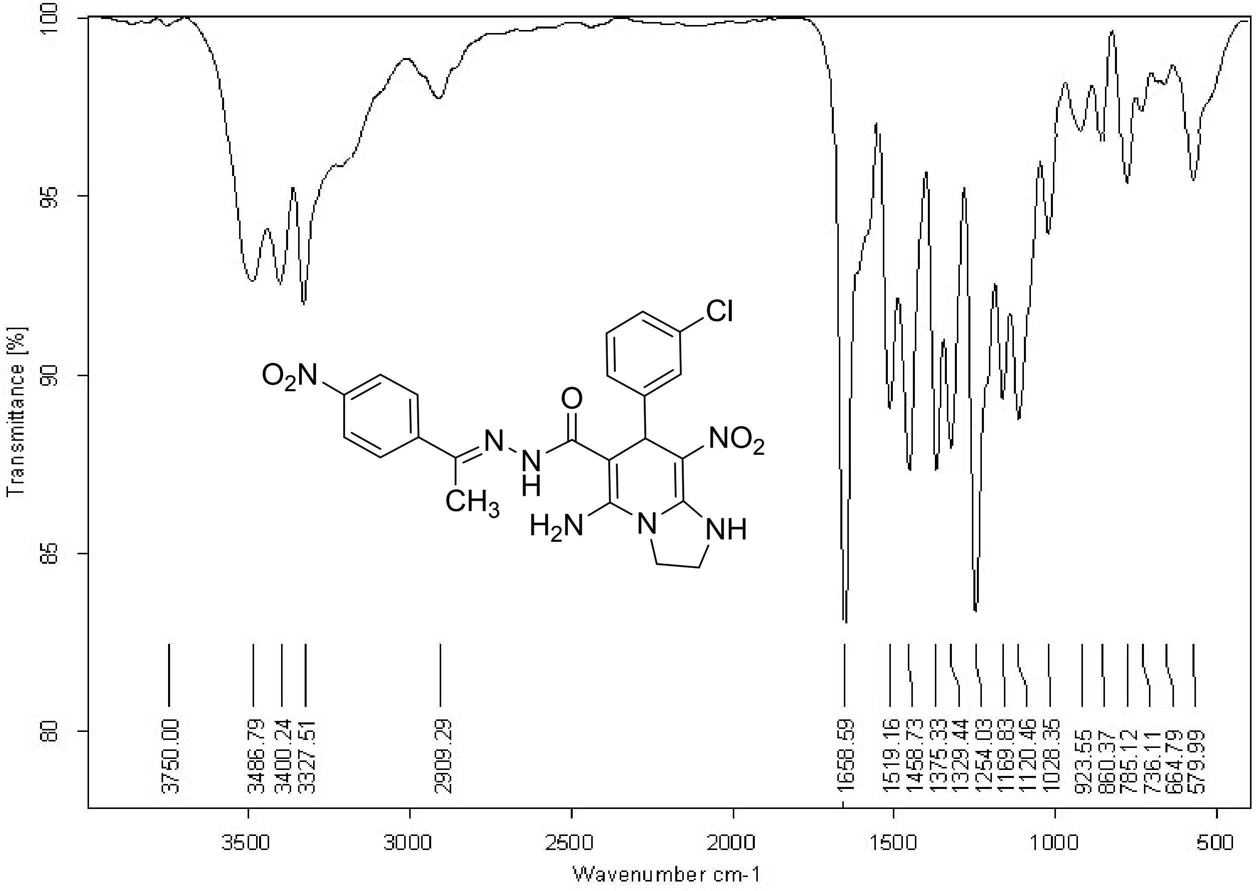

Transmittance [%]
100
95
90
85
80
Wavenumber cm-1
3500
3000
2500
2000
1500
1000
500
3750.00
3486.79
3400.24
3327.51
2909.29
1658.59
1519.16
1458.73
1375.33
1329.44
1254.03
1169.83
1120.46
1028.35
923.55
860.37
785.12
736.11
664.79
579.99
Cl
O₂N
O
NO₂
N
N
H
CH₃
H₂N
N
NH

IR of 6a

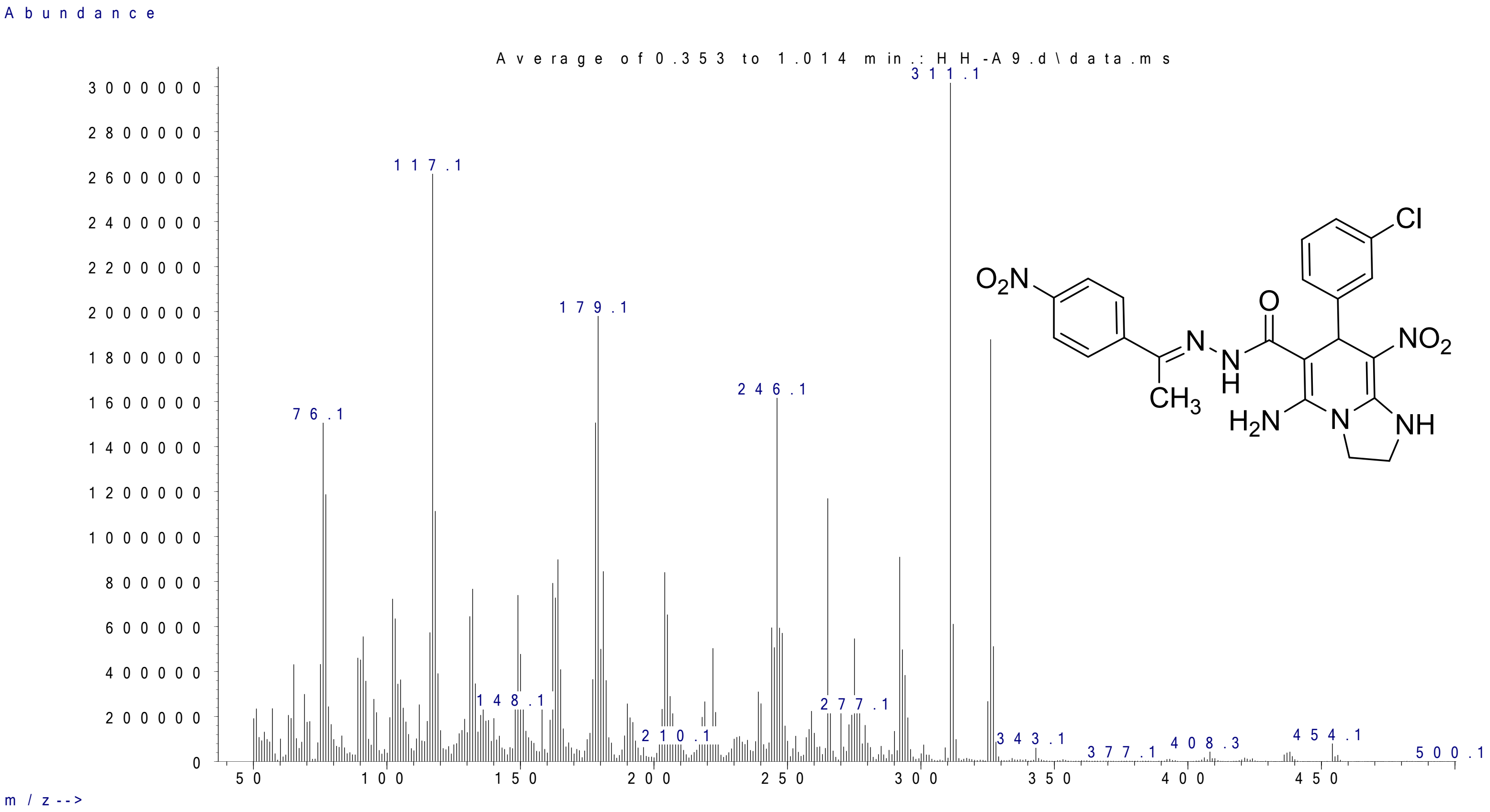
Abundance
Average of 0.353 to 1.014 min.: HH-A9.d\data.ms
m/z-->
76.1
117.1
148.1
179.1
210.1
246.1
277.1
311.1
343.1
377.1
408.3
454.1
500.1
O2N
N
N
H
O
CH3
Cl
NO2
H2N
N
NH

MS of 6a

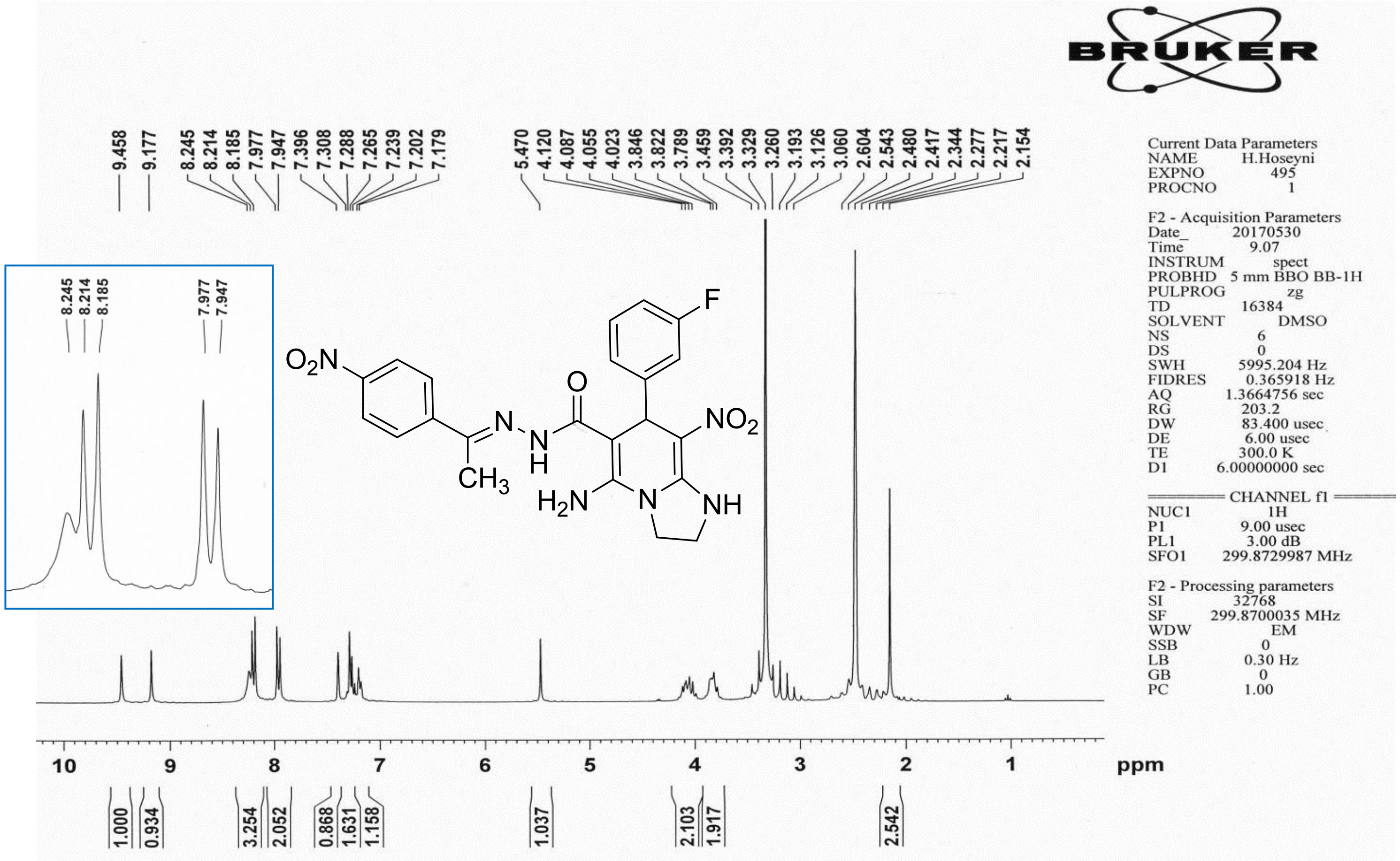

^{1}H NMR of 6b

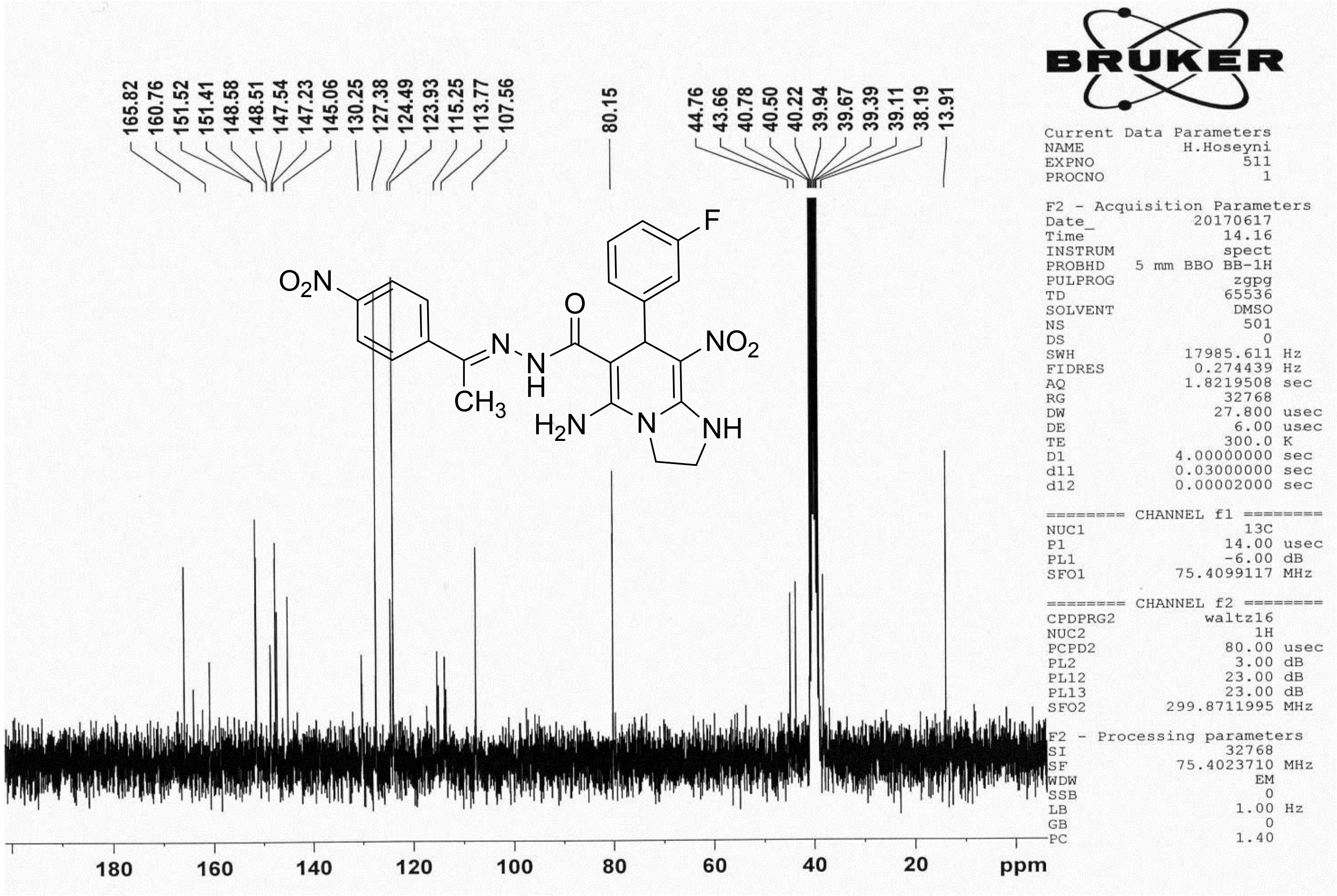
165.82
160.76
151.52
151.41
148.58
148.51
147.54
147.23
145.06
130.25
127.38
124.49
123.93
115.25
113.77
107.56
80.15
44.76
43.66
40.78
40.50
40.22
39.94
39.67
39.39
39.11
38.19
13.91
F
O_2N
O
N
N
H
NO_2
CH_3
H_2N
N
NH
BRUKER
Current Data Parameters
NAME H.Hoseyni
EXPNO 511
PROCNO 1
F2 - Acquisition Parameters
Date_ 20170617
Time 14.16
INSTRUM spect
PROBHD 5 mm BBO BB-1H
PULPROG zgpg
TD 65536
SOLVENT DMSO
NS 501
DS 0
SWH 17985.611 Hz
FIDRES 0.274439 Hz
AQ 1.8219508 sec
RG 32768
DW 27.800 usec
DE 6.00 usec
TE 300.0 K
D1 4.00000000 sec
d11 0.03000000 sec
d12 0.00002000 sec
======== CHANNEL f1 ========
NUC1 13C
P1 14.00 usec
PL1 -6.00 dB
SFO1 75.4099117 MHz
======== CHANNEL f2 ========
CPDPRG2 waltz16
NUC2 1H
PCPD2 80.00 usec
PL2 3.00 dB
PL12 23.00 dB
PL13 23.00 dB
SFO2 299.8711995 MHz
F2 - Processing parameters
SI 32768
SF 75.4023710 MHz
WDW EM
SSB 0
LB 1.00 Hz
GB 0
PC 1.40
180
160
140
120
100
80
60
40
20
ppm

^{13}C NMR of 6b

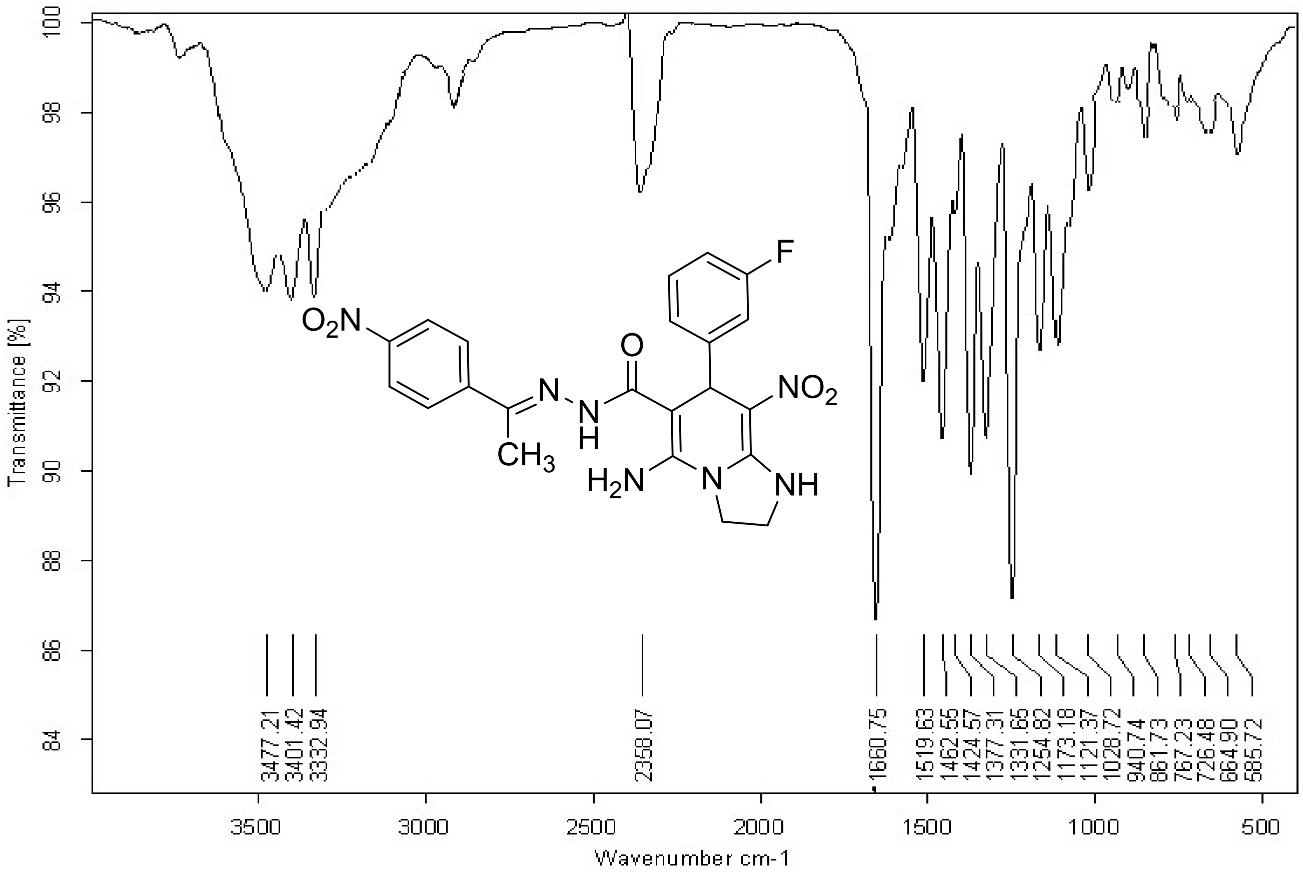
Transmittance [%]
Wavenumber cm-1
100
98
96
94
92
90
88
86
84
3500
3000
2500
2000
1500
1000
500
3477.21
3401.42
3332.94
2358.07
1660.75
1519.63
1462.55
1424.57
1377.31
1331.65
1254.82
1173.18
1121.37
1028.72
940.74
861.73
767.23
726.48
664.90
585.72
F
O
O_2N
N
N
H
NO_2
CH_3
H_2N
N
NH

IR of 6b

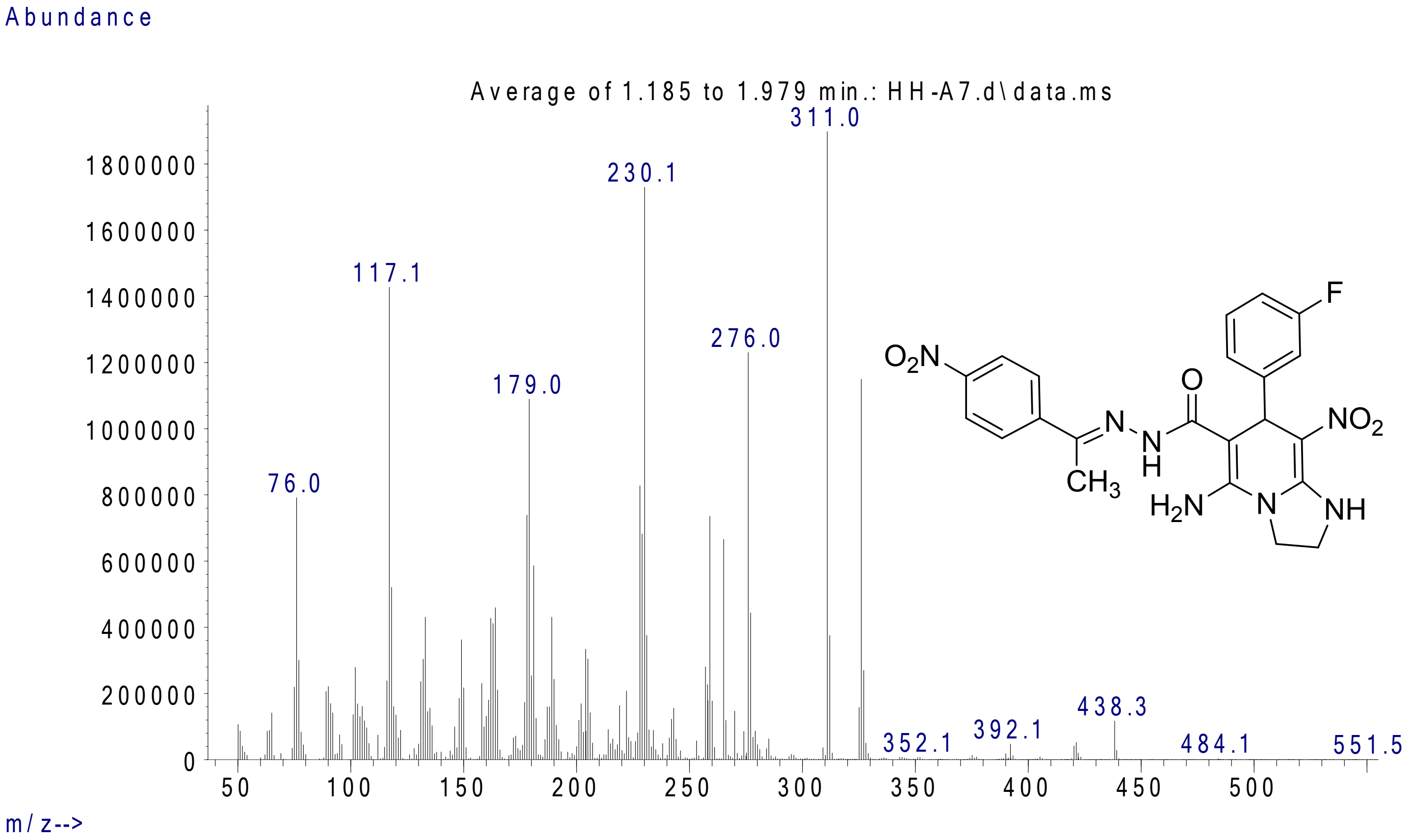
Abundance
Average of 1.185 to 1.979 min.: HH-A7.d\data.ms
311.0
230.1
117.1
179.0
276.0
76.0
352.1
392.1
438.3
484.1
551.5
1800000
1600000
1400000
1200000
1000000
800000
600000
400000
200000
0
50
100
150
200
250
300
350
400
450
500
m/z-->
O2N
N
N
H
CH3
O
F
NO2
H2N
N
NH

MS of 6b

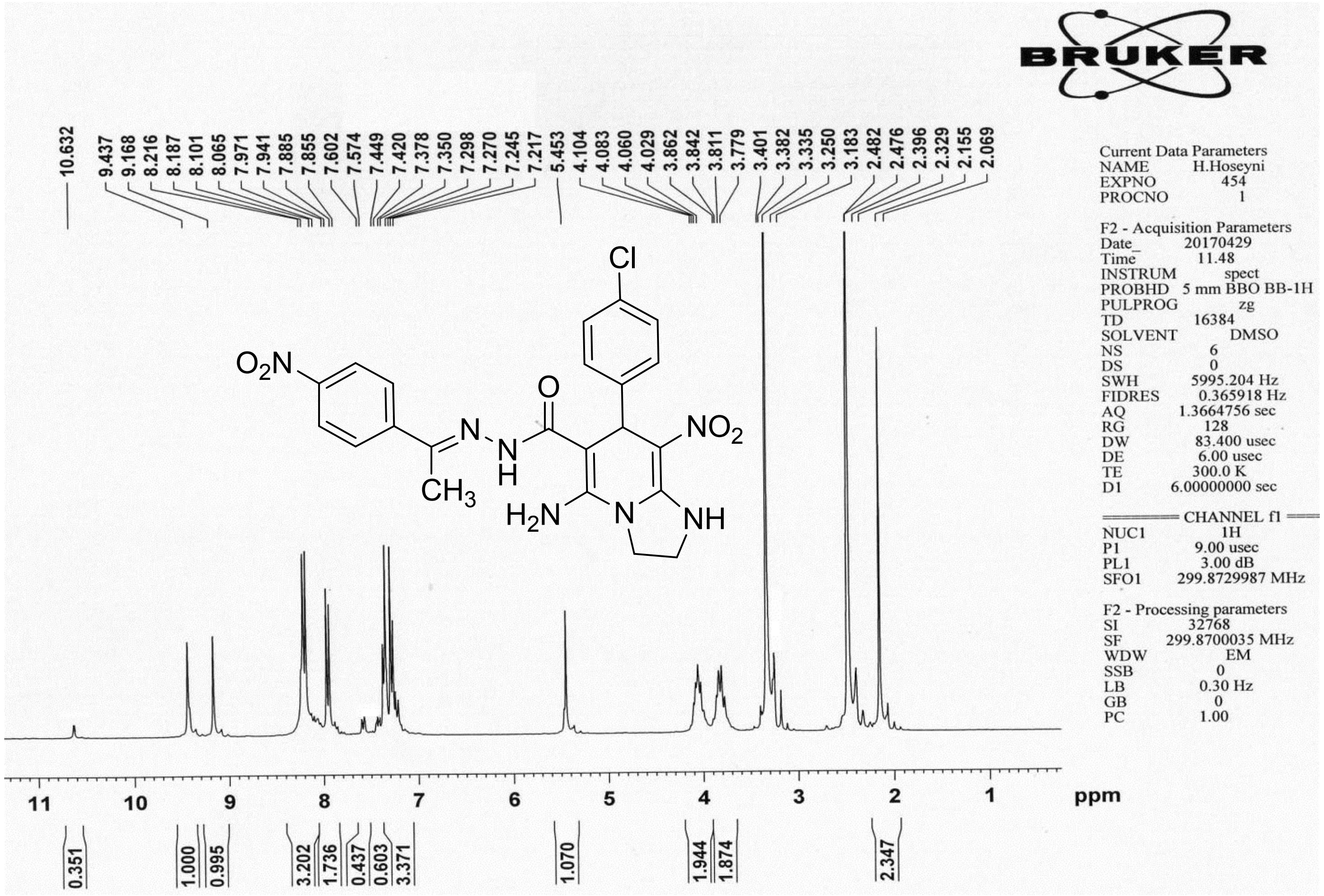

^{1}H NMR of 6c

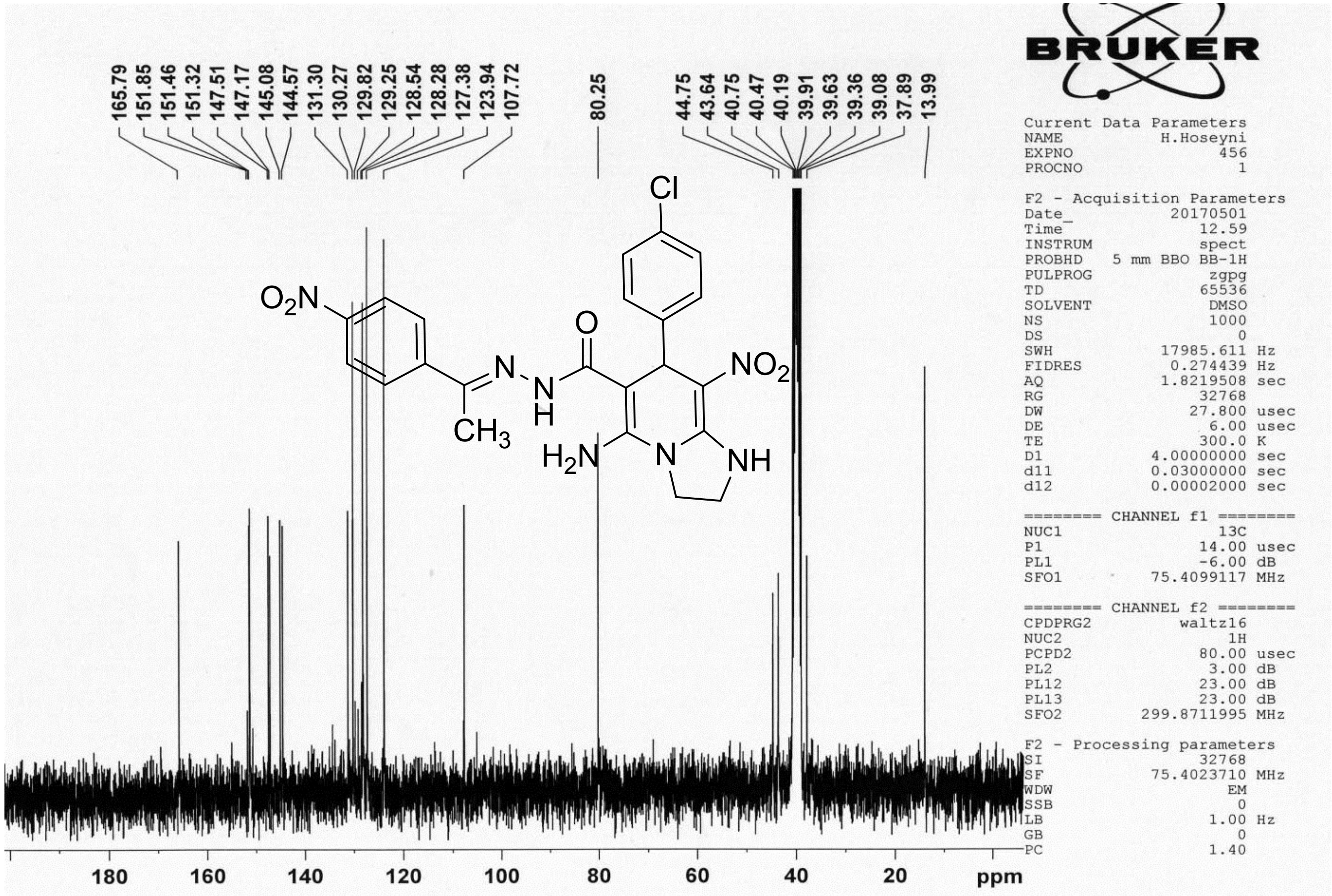
165.79
151.85
151.46
151.32
147.51
147.17
145.08
144.57
131.30
130.27
129.82
129.25
128.54
128.28
127.38
123.94
107.72
80.25
44.75
43.64
40.75
40.47
40.19
39.91
39.63
39.36
39.08
37.89
13.99
BRUKER
Current Data Parameters
NAME H.Hoseyni
EXPNO 456
PROCNO 1
F2 - Acquisition Parameters
Date_ 20170501
Time 12.59
INSTRUM spect
PROBHD 5 mm BBO BB-1H
PULPROG zgpg
TD 65536
SOLVENT DMSO
NS 1000
DS 0
SWH 17985.611 Hz
FIDRES 0.274439 Hz
AQ 1.8219508 sec
RG 32768
DW 27.800 usec
DE 6.00 usec
TE 300.0 K
D1 4.00000000 sec
d11 0.03000000 sec
d12 0.00002000 sec
======== CHANNEL f1 ========
NUC1 13C
P1 14.00 usec
PL1 -6.00 dB
SFO1 75.4099117 MHz
======== CHANNEL f2 ========
CPDPRG2 waltz16
NUC2 1H
PCPD2 80.00 usec
PL2 3.00 dB
PL12 23.00 dB
PL13 23.00 dB
SFO2 299.8711995 MHz
F2 - Processing parameters
SI 32768
SF 75.4023710 MHz
WDW EM
SSB 0
LB 1.00 Hz
GB 0
PC 1.40
180 160 140 120 100 80 60 40 20 ppm

^{13}C NMR of 6c

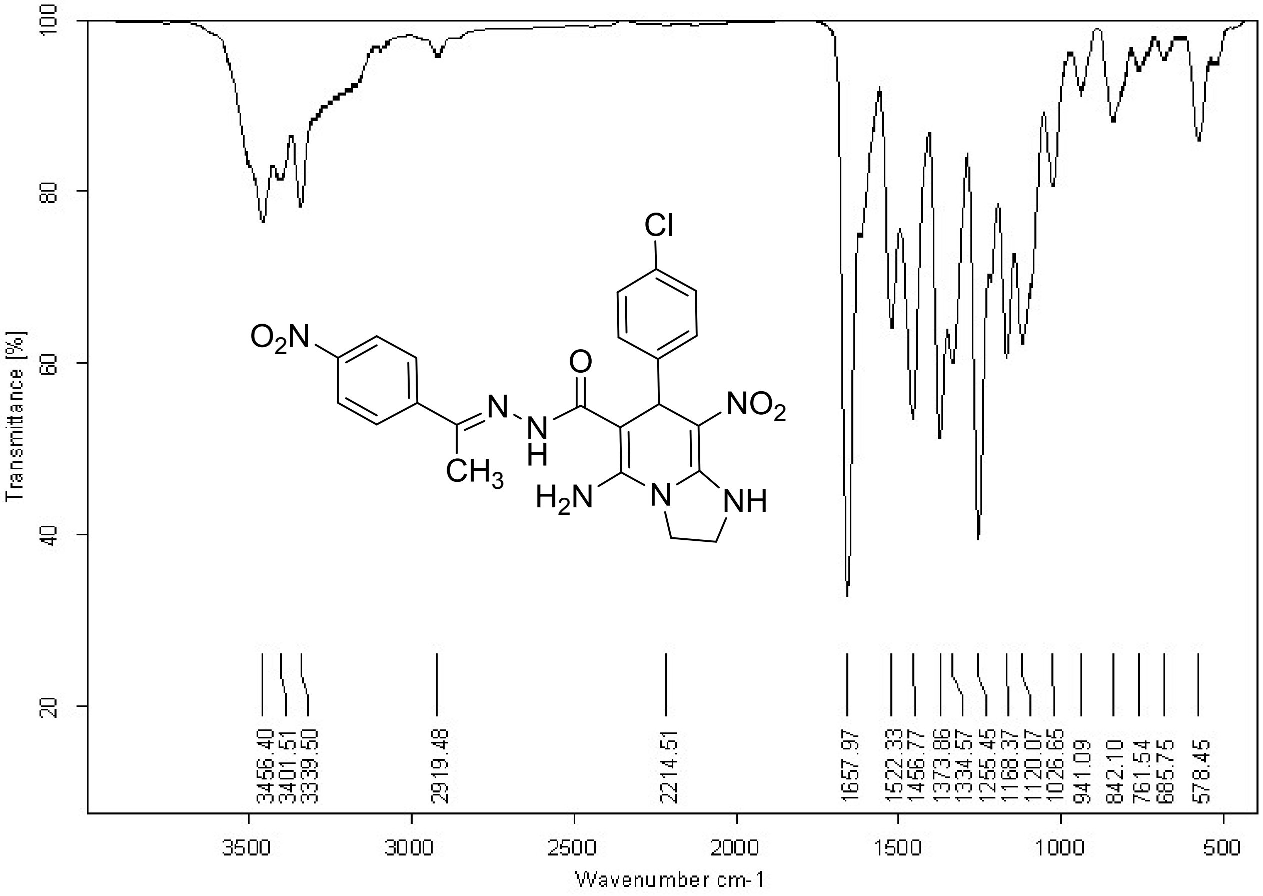
Transmittance [%]
100
80
60
40
20
Wavenumber cm-1
3500
3000
2500
2000
1500
1000
500
3456.40
3401.51
3339.50
2919.48
2214.51
1657.97
1522.33
1456.77
1373.86
1334.57
1255.45
1168.37
1120.07
1026.65
941.09
842.10
761.54
685.75
578.45
Cl
O₂N
O
N
N
H
NO₂
CH₃
H₂N
N
NH

IR of 6c

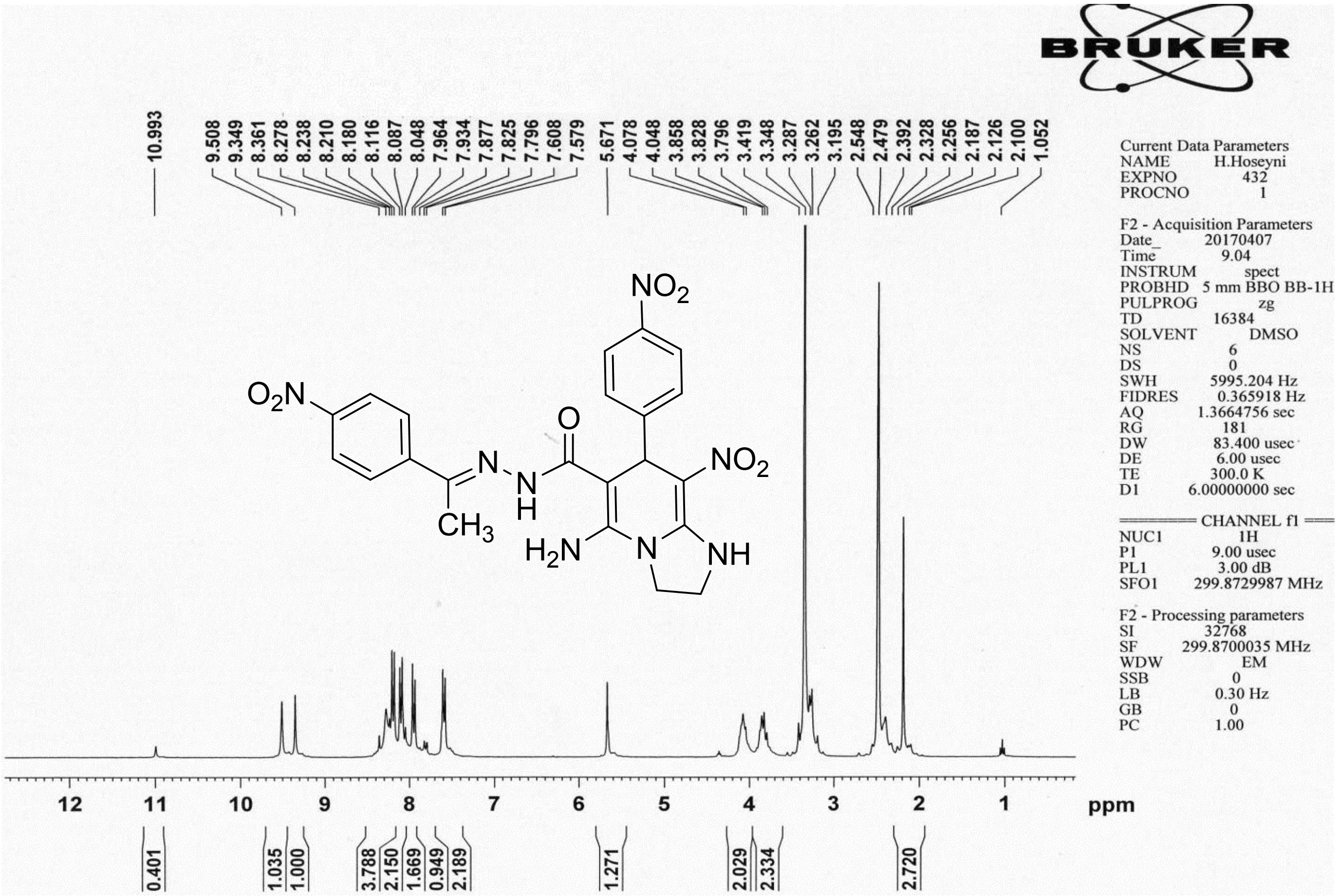
BRUKER
10.993
9.508
9.349
8.361
8.278
8.238
8.210
8.180
8.116
8.087
8.048
7.964
7.934
7.877
7.825
7.796
7.608
7.579
5.671
4.078
4.048
3.858
3.828
3.796
3.419
3.348
3.287
3.262
3.195
2.548
2.479
2.392
2.328
2.256
2.187
2.126
2.100
1.052
Current Data Parameters
NAME H.Hoseyni
EXPNO 432
PROCNO 1
F2 - Acquisition Parameters
Date_ 20170407
Time 9.04
INSTRUM spect
PROBHD 5 mm BBO BB-1H
PULPROG zg
TD 16384
SOLVENT DMSO
NS 6
DS 0
SWH 5995.204 Hz
FIDRES 0.365918 Hz
AQ 1.3664756 sec
RG 181
DW 83.400 usec
DE 6.00 usec
TE 300.0 K
D1 6.00000000 sec
CHANNEL f1
NUC1 1H
P1 9.00 usec
PL1 3.00 dB
SFO1 299.8729987 MHz
F2 - Processing parameters
SI 32768
SF 299.8700035 MHz
WDW EM
SSB 0
LB 0.30 Hz
GB 0
PC 1.00
ppm
0.401
1.035
1.000
3.788
2.150
1.669
0.949
2.189
1.271
2.029
2.334
2.720

^{1}H NMR of 6d

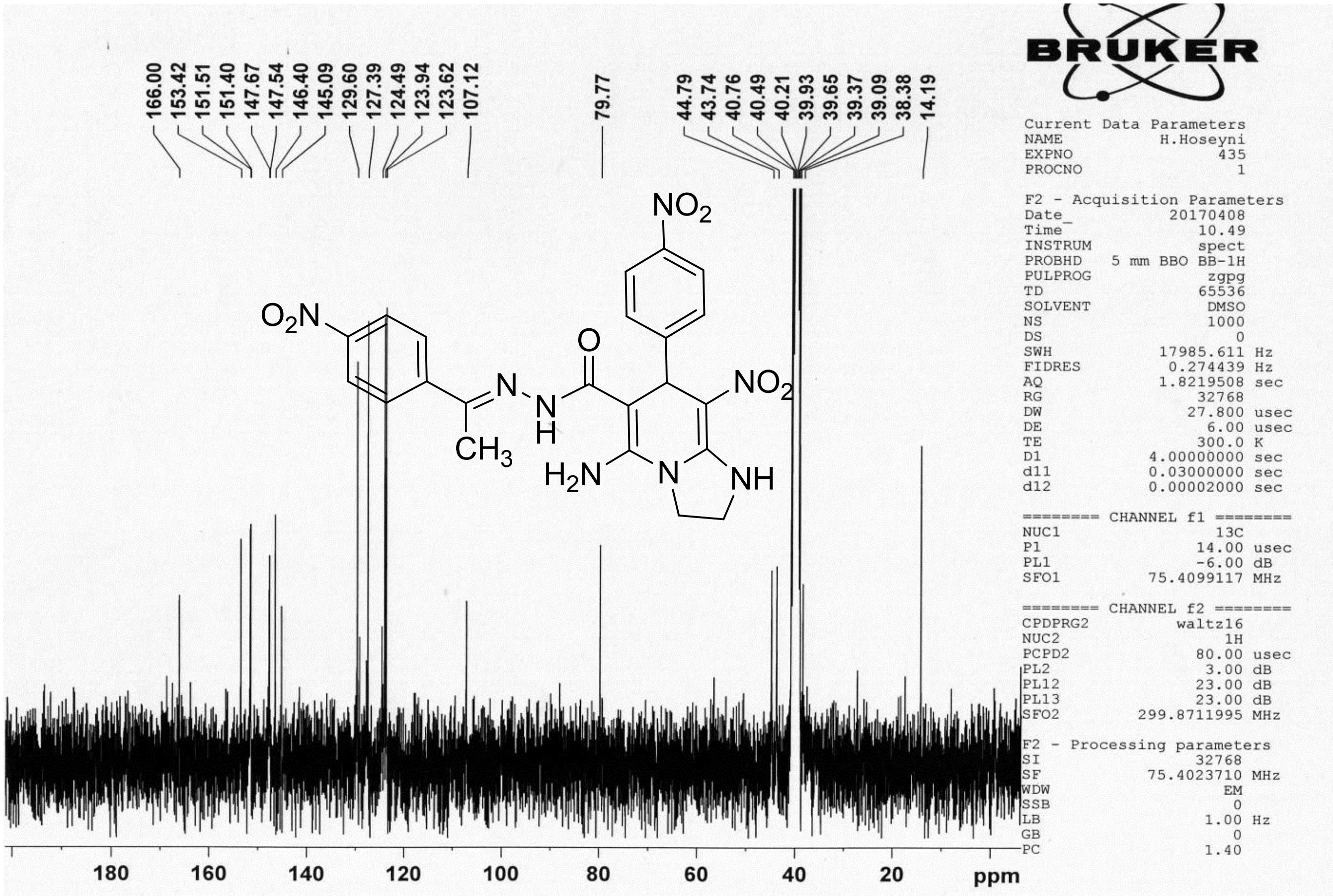

166.00
153.42
151.51
151.40
147.67
147.54
146.40
145.09
129.60
127.39
124.49
123.94
123.62
107.12
79.77
44.79
43.74
40.76
40.49
40.21
39.93
39.65
39.37
39.09
38.38
14.19
BRUKER
Current Data Parameters
NAME H.Hoseyni
EXPNO 435
PROCNO 1
F2 - Acquisition Parameters
Date_ 20170408
Time 10.49
INSTRUM spect
PROBHD 5 mm BBO BB-1H
PULPROG zgpg
TD 65536
SOLVENT DMSO
NS 1000
DS 0
SWH 17985.611 Hz
FIDRES 0.274439 Hz
AQ 1.8219508 sec
RG 32768
DW 27.800 usec
DE 6.00 usec
TE 300.0 K
D1 4.00000000 sec
d11 0.03000000 sec
d12 0.00002000 sec
======== CHANNEL f1 ========
NUC1 13C
P1 14.00 usec
PL1 -6.00 dB
SFO1 75.4099117 MHz
======== CHANNEL f2 ========
CPDPRG2 waltz16
NUC2 1H
PCPD2 80.00 usec
PL2 3.00 dB
PL12 23.00 dB
PL13 23.00 dB
SFO2 299.8711995 MHz
F2 - Processing parameters
SI 32768
SF 75.4023710 MHz
WDW EM
SSB 0
LB 1.00 Hz
GB 0
PC 1.40
180
160
140
120
100
80
60
40
20
ppm

^{13}C NMR of 6d

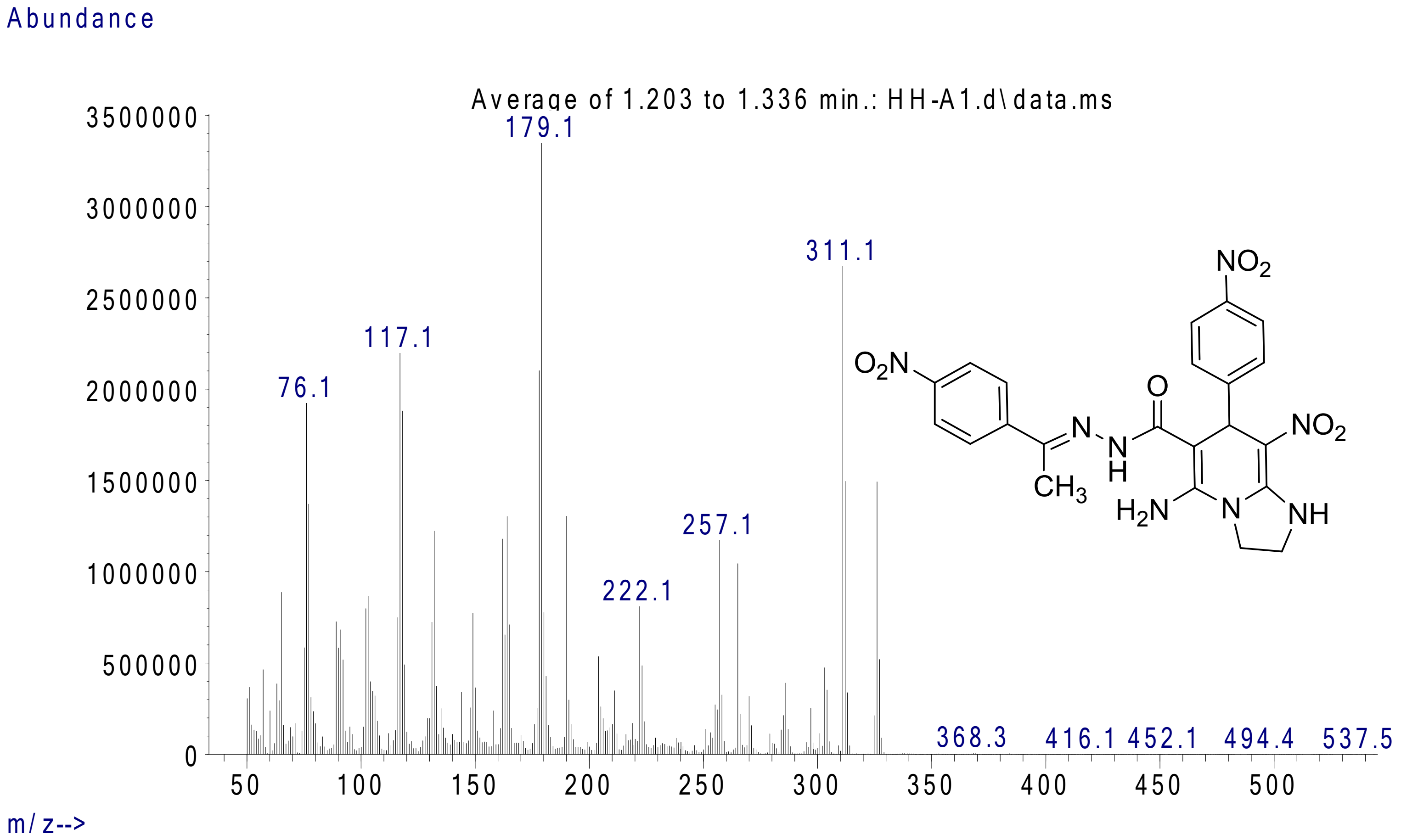
Abundance
Average of 1.203 to 1.336 min.: HH-A1.d\data.ms
3500000
3000000
2500000
2000000
1500000
1000000
500000
0
179.1
311.1
117.1
76.1
257.1
222.1
368.3
416.1
452.1
494.4
537.5
50
100
150
200
250
300
350
400
450
500
m/z-->
NO2
O2N
O
N
N
H
NO2
CH3
H2N
N
NH

MS of 6d

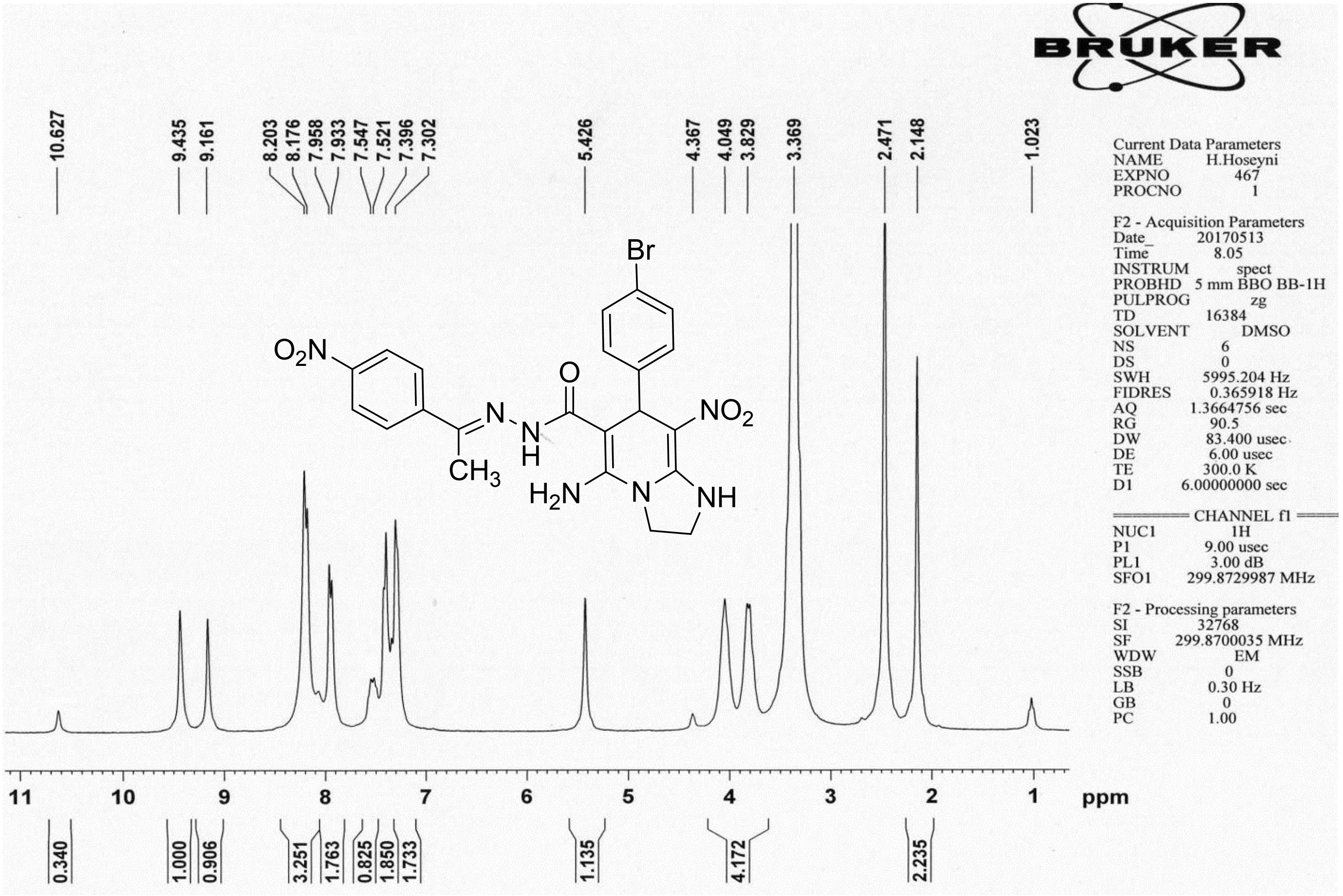
BRUKER
10.627
9.435
9.161
8.203
8.176
7.958
7.933
7.547
7.521
7.396
7.302
5.426
4.367
4.049
3.829
3.369
2.471
2.148
1.023
Br
O_2N
O
N
N
H
NO_2
CH_3
H_2N
N
NH
Current Data Parameters
NAME H.Hoseyni
EXPNO 467
PROCNO 1
F2 - Acquisition Parameters
Date_ 20170513
Time 8.05
INSTRUM spect
PROBHD 5 mm BBO BB-1H
PULPROG zg
TD 16384
SOLVENT DMSO
NS 6
DS 0
SWH 5995.204 Hz
FIDRES 0.365918 Hz
AQ 1.3664756 sec
RG 90.5
DW 83.400 usec
DE 6.00 usec
TE 300.0 K
D1 6.00000000 sec
CHANNEL f1
NUC1 1H
P1 9.00 usec
PL1 3.00 dB
SFO1 299.8729987 MHz
F2 - Processing parameters
SI 32768
SF 299.8700035 MHz
WDW EM
SSB 0
LB 0.30 Hz
GB 0
PC 1.00
11 10 9 8 7 6 5 4 3 2 1 ppm
0.340
1.000
0.906
3.251
1.763
0.825
1.850
1.733
1.135
4.172
2.235

^{1}H NMR of 6e

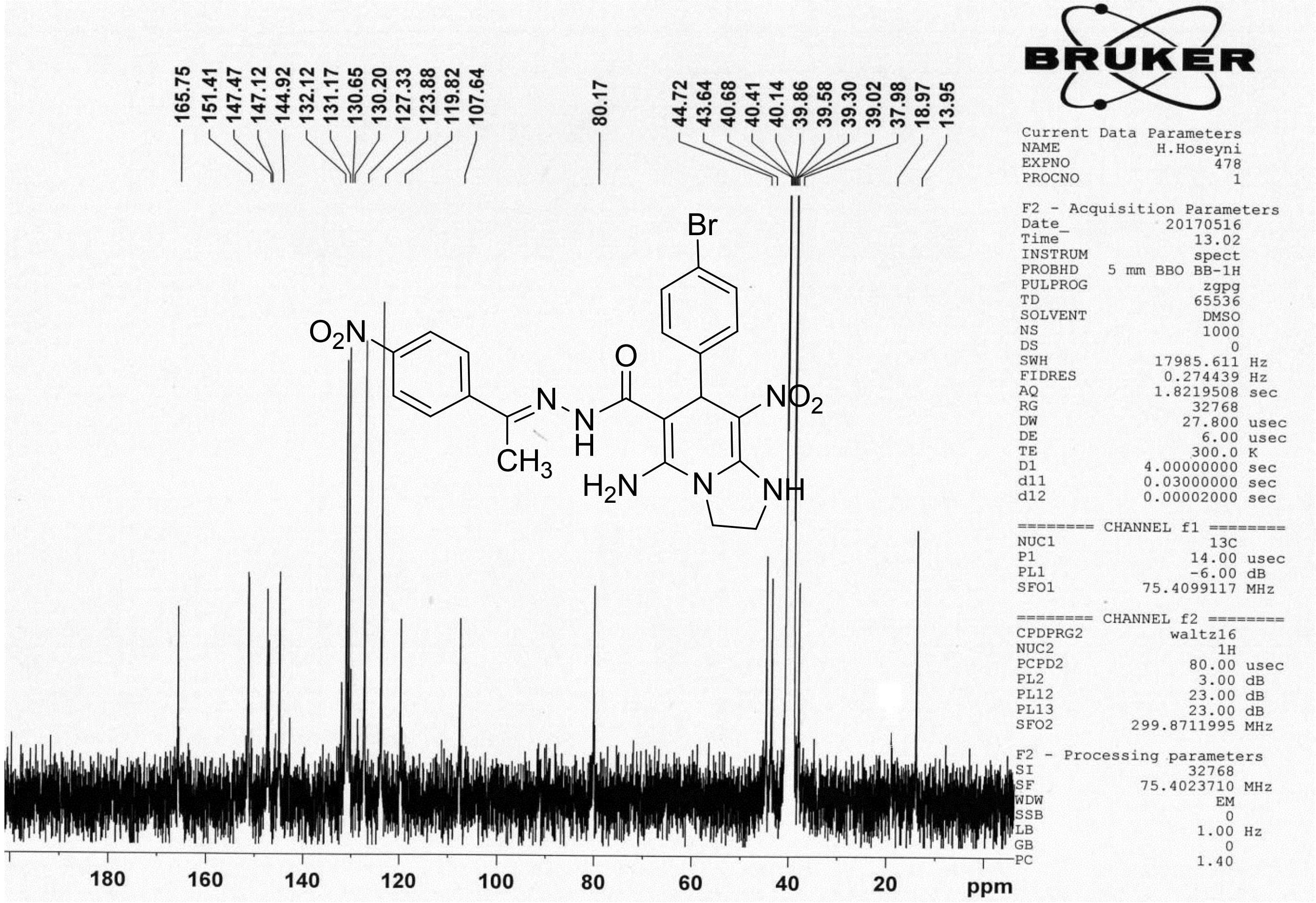
165.75
151.41
147.47
147.12
144.92
132.12
131.17
130.65
130.20
127.33
123.88
119.82
107.64
80.17
44.72
43.64
40.68
40.41
40.14
39.86
39.58
39.30
39.02
37.98
18.97
13.95
BRUKER
Current Data Parameters
NAME H.Hoseyni
EXPNO 478
PROCNO 1
F2 - Acquisition Parameters
Date_ 20170516
Time 13.02
INSTRUM spect
PROBHD 5 mm BBO BB-1H
PULPROG zgpg
TD 65536
SOLVENT DMSO
NS 1000
DS 0
SWH 17985.611 Hz
FIDRES 0.274439 Hz
AQ 1.8219508 sec
RG 32768
DW 27.800 usec
DE 6.00 usec
TE 300.0 K
D1 4.00000000 sec
d11 0.03000000 sec
d12 0.00002000 sec
======== CHANNEL f1 ========
NUC1 13C
P1 14.00 usec
PL1 -6.00 dB
SFO1 75.4099117 MHz
======== CHANNEL f2 ========
CPDPRG2 waltz16
NUC2 1H
PCPD2 80.00 usec
PL2 3.00 dB
PL12 23.00 dB
PL13 23.00 dB
SFO2 299.8711995 MHz
F2 - Processing parameters
SI 32768
SF 75.4023710 MHz
WDW EM
SSB 0
LB 1.00 Hz
GB 0
PC 1.40
180
160
140
120
100
80
60
40
20
ppm

^{13}C NMR of 6e

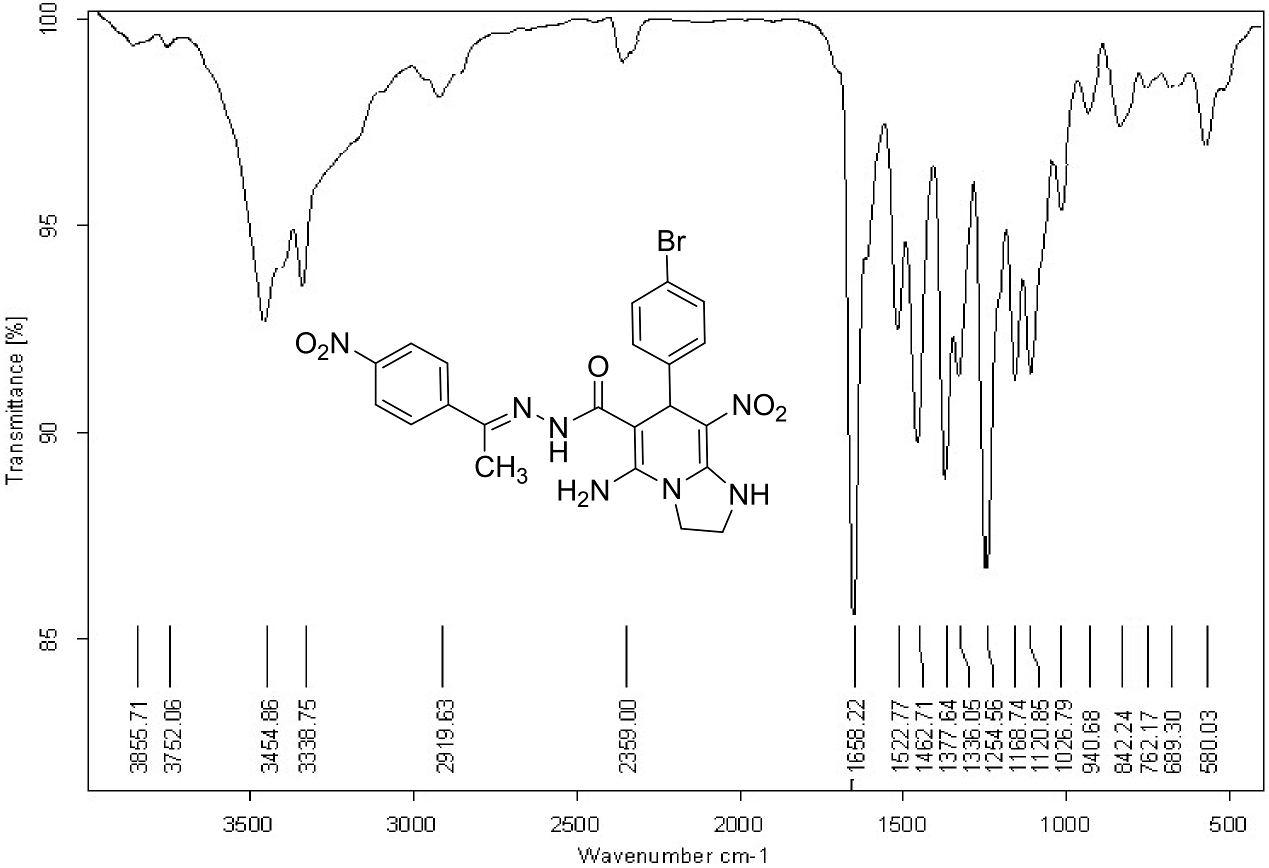
Transmittance [%]
100
95
90
85
Wavenumber cm-1
3500
3000
2500
2000
1500
1000
500
3855.71
3752.06
3454.86
3338.75
2919.63
2359.00
1658.22
1522.77
1462.71
1377.64
1336.05
1254.56
1168.74
1120.85
1026.79
940.68
842.24
762.17
689.30
580.03
Br
O
O_2N
N
N
H
CH_3
NO_2
H_2N
N
NH

IR of 6e

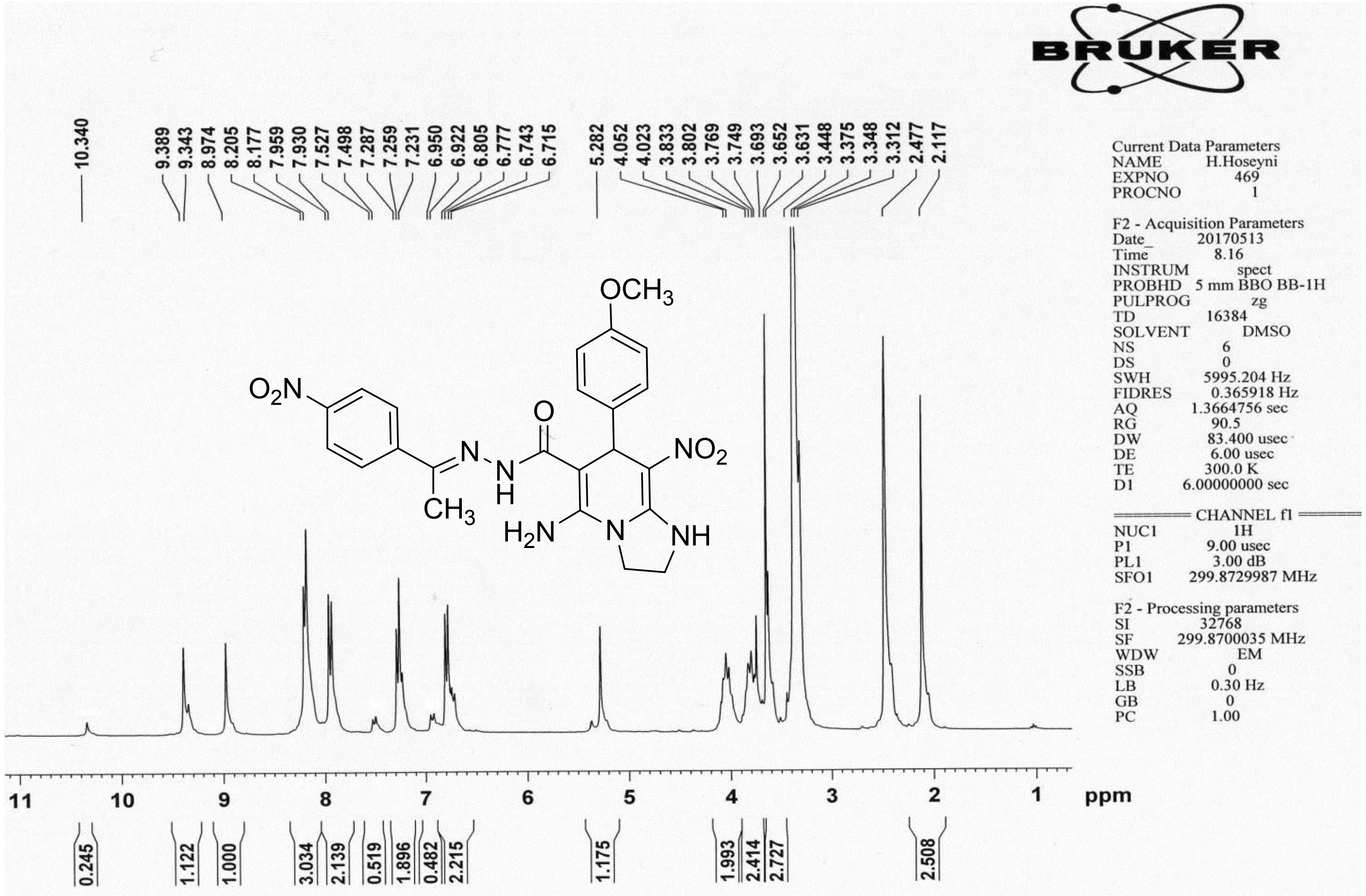
BRUKER
10.340
9.389
9.343
8.974
8.205
8.177
7.959
7.930
7.527
7.498
7.287
7.259
7.231
6.950
6.922
6.805
6.777
6.743
6.715
5.282
4.052
4.023
3.833
3.802
3.769
3.749
3.693
3.652
3.631
3.448
3.375
3.348
3.312
2.477
2.117
Current Data Parameters
NAME H.Hoseyni
EXPNO 469
PROCNO 1
F2 - Acquisition Parameters
Date_ 20170513
Time 8.16
INSTRUM spect
PROBHD 5 mm BBO BB-1H
PULPROG zg
TD 16384
SOLVENT DMSO
NS 6
DS 0
SWH 5995.204 Hz
FIDRES 0.365918 Hz
AQ 1.3664756 sec
RG 90.5
DW 83.400 usec
DE 6.00 usec
TE 300.0 K
D1 6.00000000 sec
CHANNEL f1
NUC1 1H
P1 9.00 usec
PL1 3.00 dB
SFO1 299.8729987 MHz
F2 - Processing parameters
SI 32768
SF 299.8700035 MHz
WDW EM
SSB 0
LB 0.30 Hz
GB 0
PC 1.00
OCH3
O2N
O
N
N
H
NO2
CH3
H2N
N
NH
11
10
9
8
7
6
5
4
3
2
1
ppm
0.245
1.122
1.000
3.034
2.139
0.519
1.896
0.482
2.215
1.175
1.993
2.414
2.727
2.508

^{1}H NMR of 6f

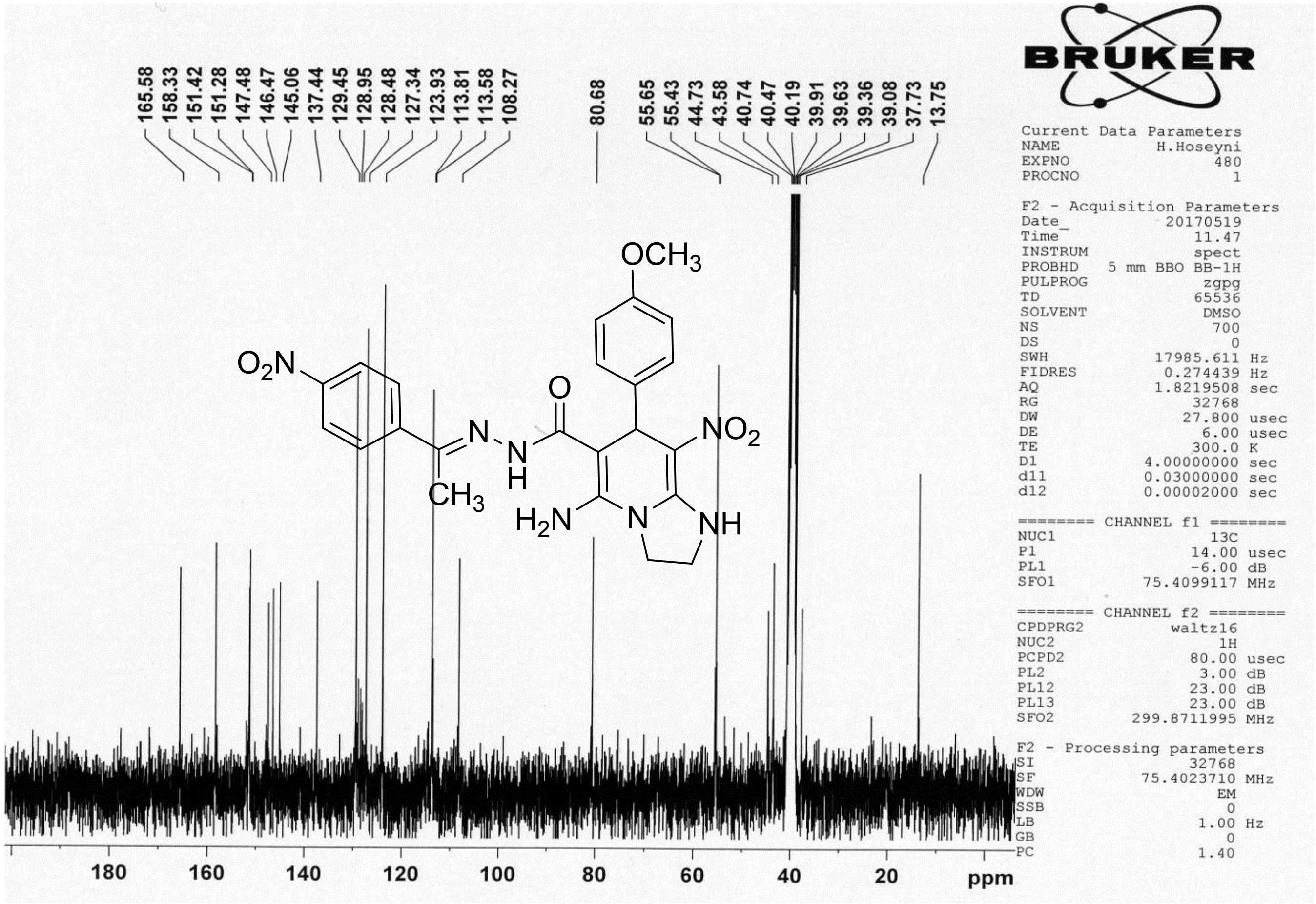

^{13}C NMR of 6f

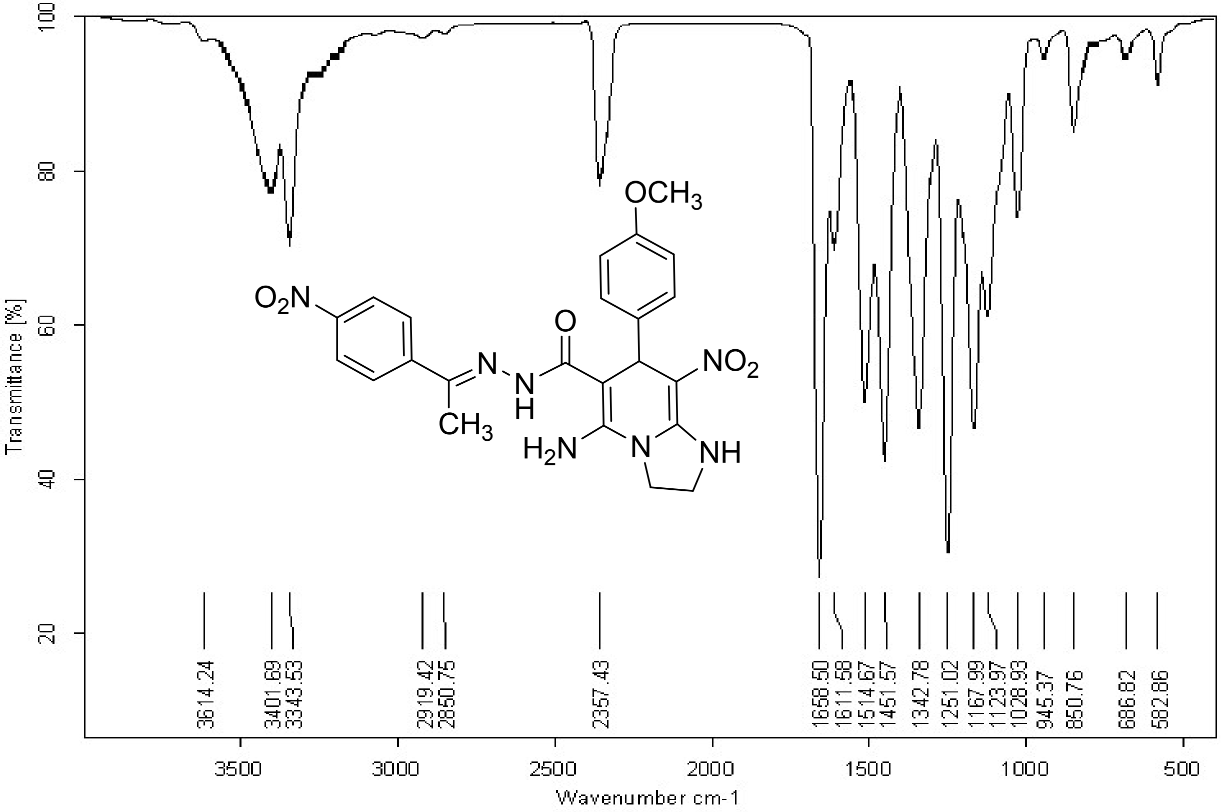
100
80
60
40
20
Transmittance [%]
3614.24
3401.69
3343.53
2919.42
2850.75
2357.43
1658.50
1611.58
1514.67
1451.57
1342.78
1251.02
1167.99
1123.97
1028.93
945.37
850.76
686.82
582.86
3500
3000
2500
2000
1500
1000
500
Wavenumber cm-1
OCH3
O2N
O
N
N
H
NO2
CH3
H2N
N
NH

IR of 6f

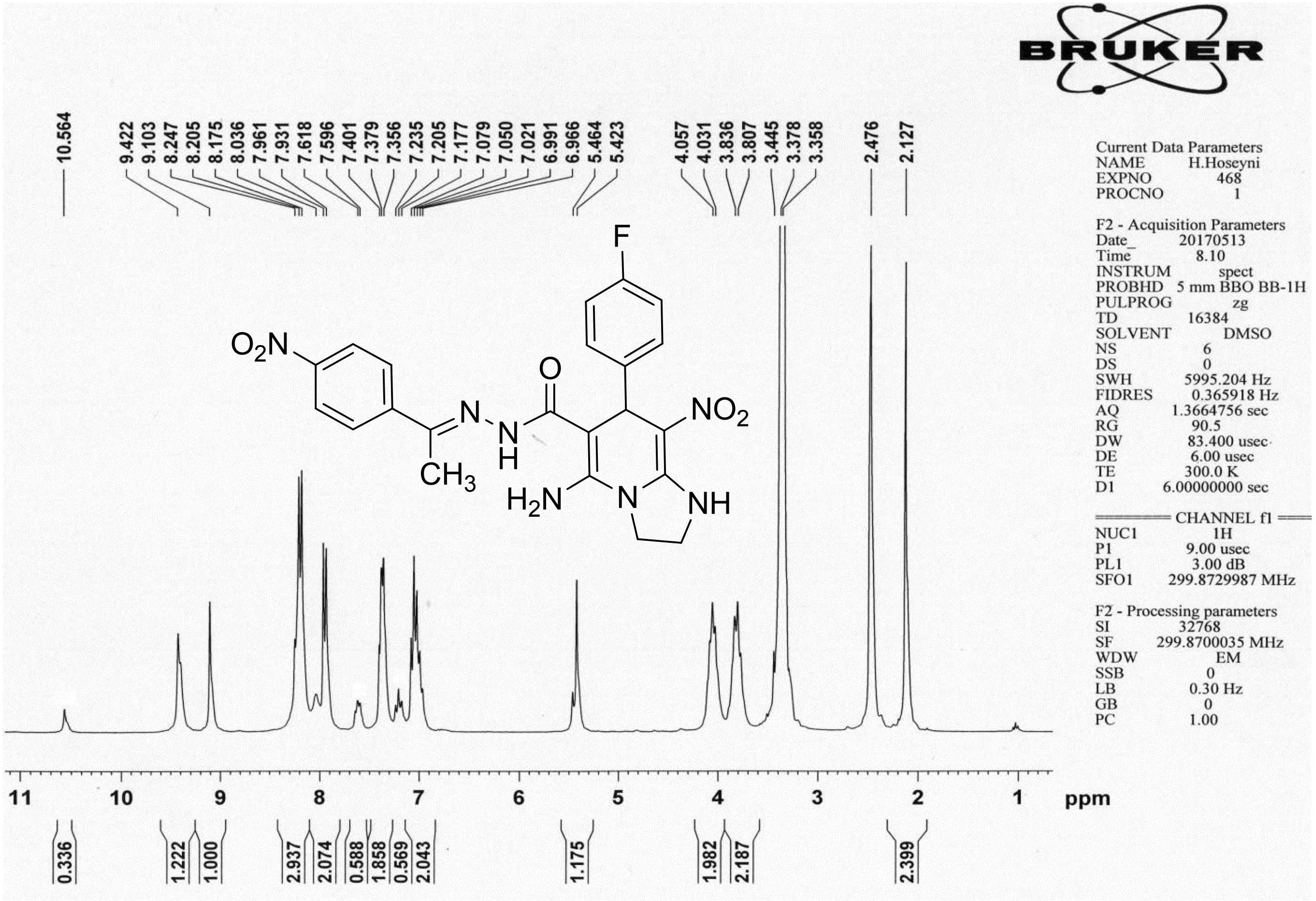
BRUKER
10.564
9.422
9.103
8.247
8.205
8.175
8.036
7.961
7.931
7.618
7.596
7.401
7.379
7.356
7.235
7.205
7.177
7.079
7.050
7.021
6.991
6.966
5.464
5.423
4.057
4.031
3.836
3.807
3.445
3.378
3.358
2.476
2.127
Current Data Parameters
NAME H.Hoseyni
EXPNO 468
PROCNO 1
F2 - Acquisition Parameters
Date_ 20170513
Time 8.10
INSTRUM spect
PROBHD 5 mm BBO BB-1H
PULPROG zg
TD 16384
SOLVENT DMSO
NS 6
DS 0
SWH 5995.204 Hz
FIDRES 0.365918 Hz
AQ 1.3664756 sec
RG 90.5
DW 83.400 usec
DE 6.00 usec
TE 300.0 K
D1 6.00000000 sec
CHANNEL f1
NUC1 1H
P1 9.00 usec
PL1 3.00 dB
SFO1 299.8729987 MHz
F2 - Processing parameters
SI 32768
SF 299.8700035 MHz
WDW EM
SSB 0
LB 0.30 Hz
GB 0
PC 1.00
F
O
NO2
O2N
N
N
H
CH3
H2N
N
NH
11 10 9 8 7 6 5 4 3 2 1 ppm
0.336
1.222
1.000
2.937
2.074
0.588
1.858
0.569
2.043
1.175
1.982
2.187
2.399

^{1}H NMR of 6g

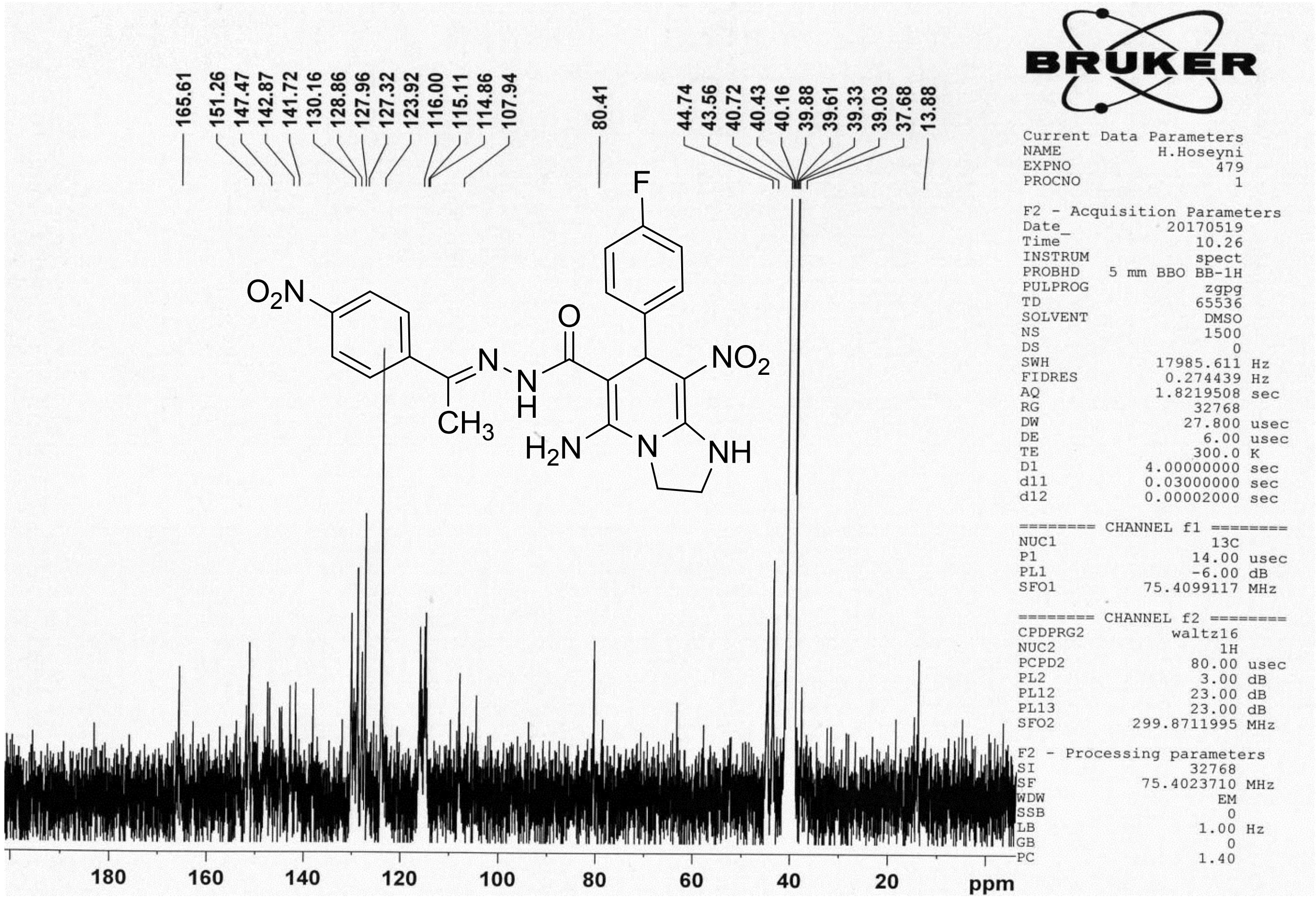

^{13}C NMR of 6g

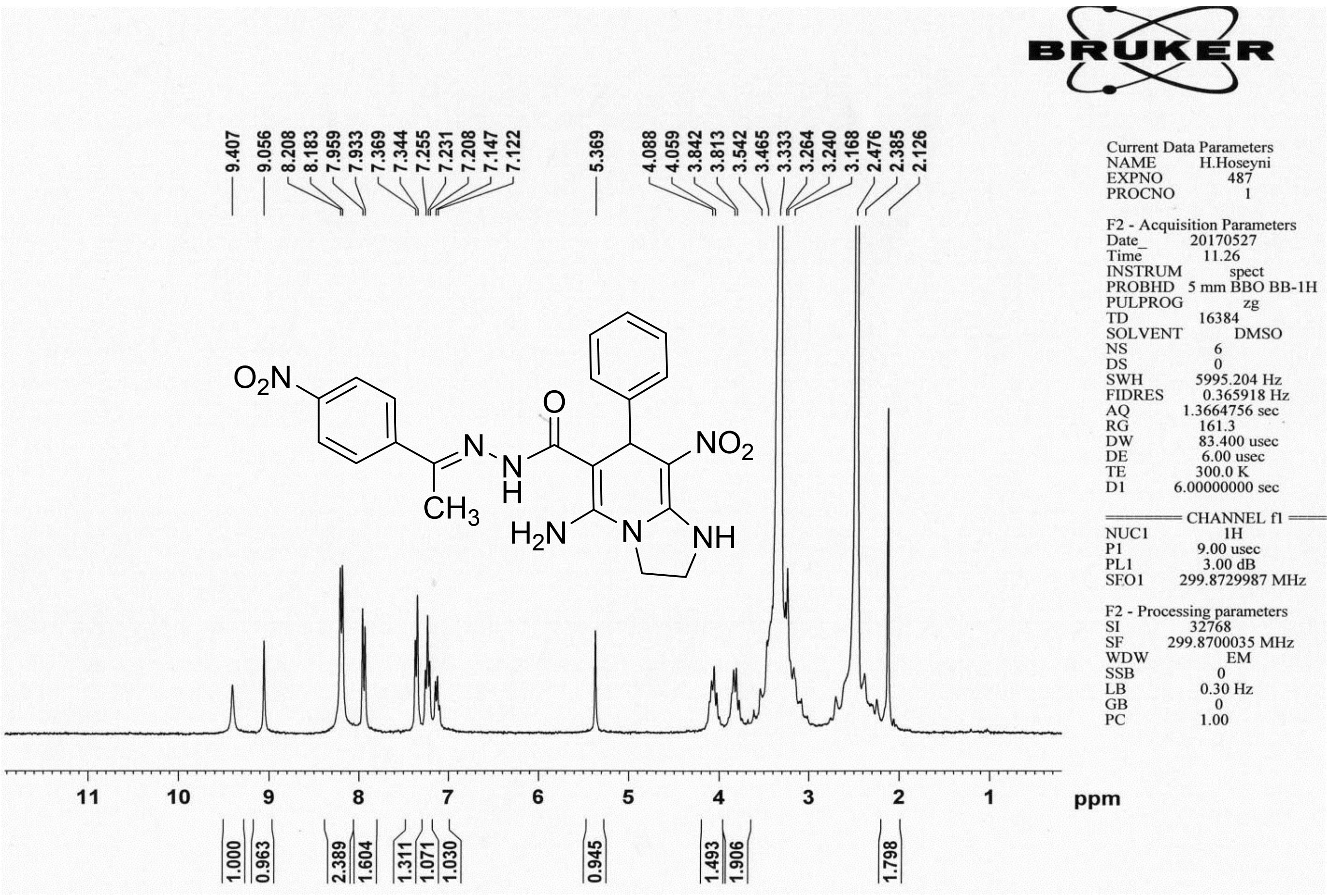

BRUKER
9.407
9.056
8.208
8.183
7.959
7.933
7.369
7.344
7.255
7.231
7.208
7.147
7.122
5.369
4.088
4.059
3.842
3.813
3.542
3.465
3.333
3.264
3.240
3.168
2.476
2.385
2.126
Current Data Parameters
NAME H.Hoseyni
EXPNO 487
PROCNO 1
F2 - Acquisition Parameters
Date_ 20170527
Time 11.26
INSTRUM spect
PROBHD 5 mm BBO BB-1H
PULPROG zg
TD 16384
SOLVENT DMSO
NS 6
DS 0
SWH 5995.204 Hz
FIDRES 0.365918 Hz
AQ 1.3664756 sec
RG 161.3
DW 83.400 usec
DE 6.00 usec
TE 300.0 K
D1 6.00000000 sec
CHANNEL f1
NUC1 1H
P1 9.00 usec
PL1 3.00 dB
SFO1 299.8729987 MHz
F2 - Processing parameters
SI 32768
SF 299.8700035 MHz
WDW EM
SSB 0
LB 0.30 Hz
GB 0
PC 1.00
O2N
O
N
N
H
CH3
NO2
H2N
N
NH
11
10
9
8
7
6
5
4
3
2
1
ppm
1.000
0.963
2.389
1.604
1.311
1.071
1.030
0.945
1.493
1.906
1.798

^{1}H NMR of 6h

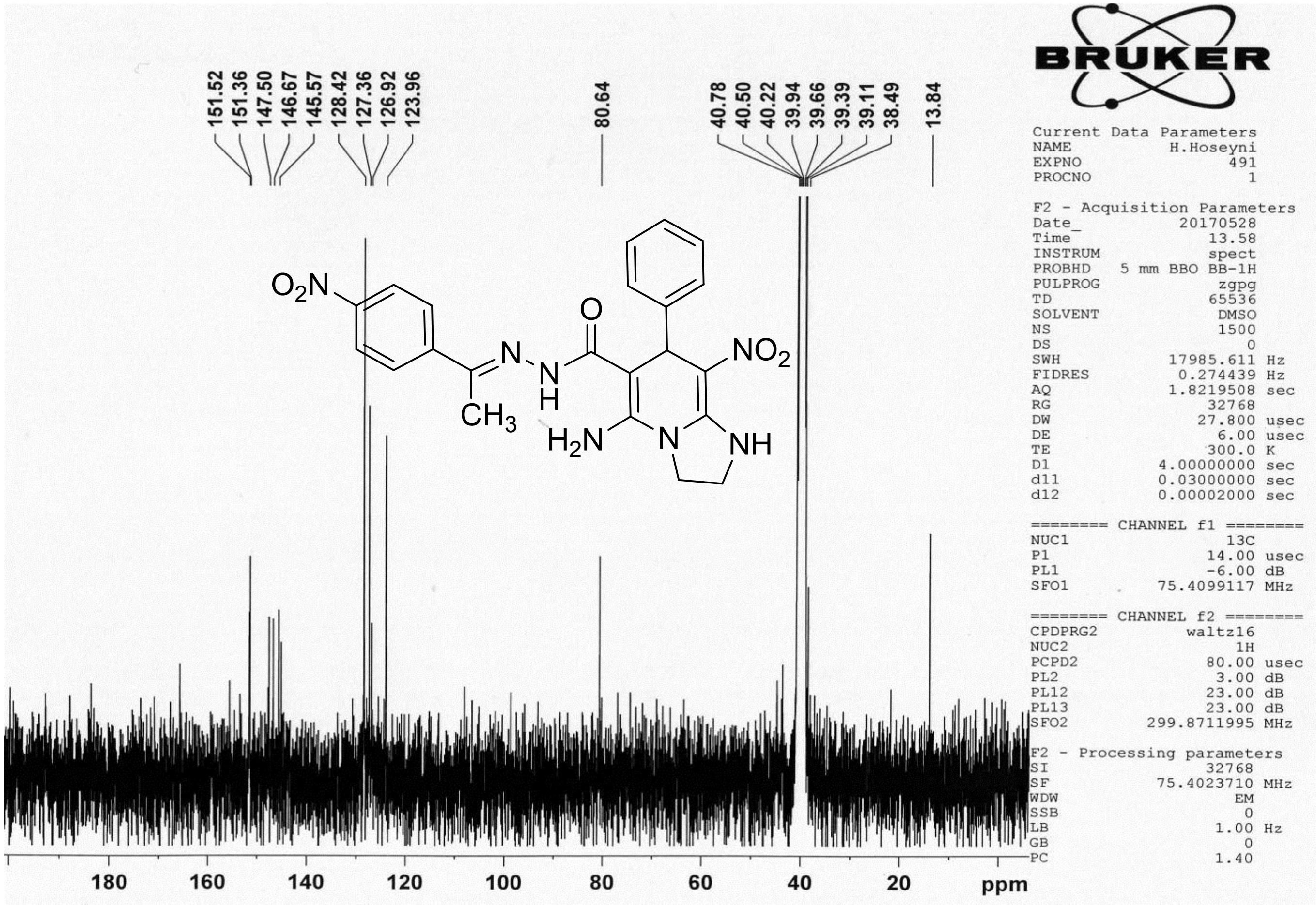

151.52
151.36
147.50
146.67
145.57
128.42
127.36
126.92
123.96
80.64
40.78
40.50
40.22
39.94
39.66
39.39
39.11
38.49
13.84
BRUKER
O2N
N
N
H
O
CH3
NO2
H2N
N
NH
Current Data Parameters
NAME H.Hoseyni
EXPNO 491
PROCNO 1
F2 - Acquisition Parameters
Date_ 20170528
Time 13.58
INSTRUM spect
PROBHD 5 mm BBO BB-1H
PULPROG zgpg
TD 65536
SOLVENT DMSO
NS 1500
DS 0
SWH 17985.611 Hz
FIDRES 0.274439 Hz
AQ 1.8219508 sec
RG 32768
DW 27.800 usec
DE 6.00 usec
TE 300.0 K
D1 4.00000000 sec
d11 0.03000000 sec
d12 0.00002000 sec
======== CHANNEL f1 ========
NUC1 13C
P1 14.00 usec
PL1 -6.00 dB
SFO1 75.4099117 MHz
======== CHANNEL f2 ========
CPDPRG2 waltz16
NUC2 1H
PCPD2 80.00 usec
PL2 3.00 dB
PL12 23.00 dB
PL13 23.00 dB
SFO2 299.8711995 MHz
F2 - Processing parameters
SI 32768
SF 75.4023710 MHz
WDW EM
SSB 0
LB 1.00 Hz
GB 0
PC 1.40
180
160
140
120
100
80
60
40
20
ppm

^{13}C NMR of 6h

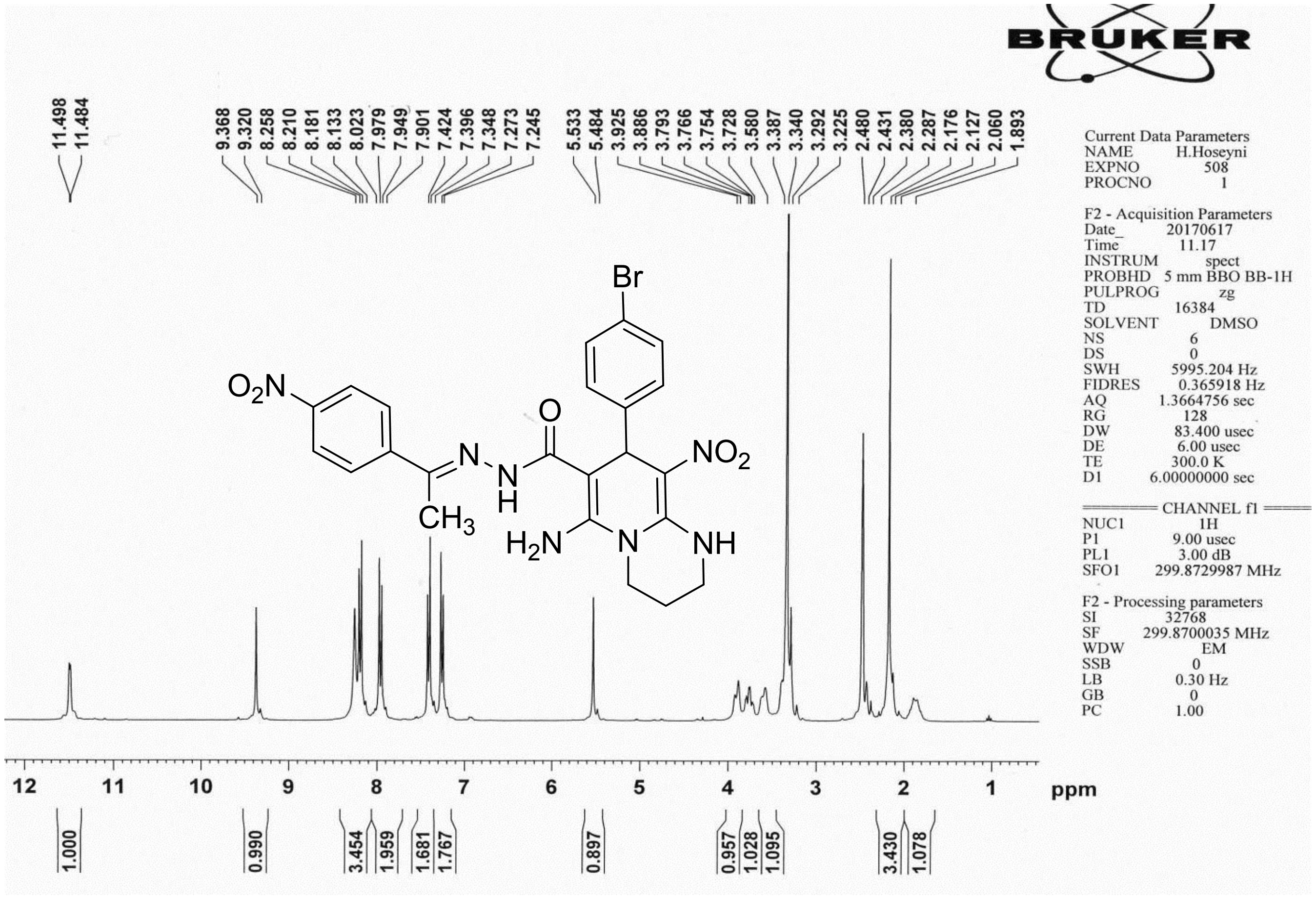
BRUKER
Current Data Parameters
NAME H.Hoseyni
EXPNO 508
PROCNO 1
F2 - Acquisition Parameters
Date_ 20170617
Time 11.17
INSTRUM spect
PROBHD 5 mm BBO BB-1H
PULPROG zg
TD 16384
SOLVENT DMSO
NS 6
DS 0
SWH 5995.204 Hz
FIDRES 0.365918 Hz
AQ 1.3664756 sec
RG 128
DW 83.400 usec
DE 6.00 usec
TE 300.0 K
D1 6.00000000 sec
CHANNEL f1
NUC1 1H
P1 9.00 usec
PL1 3.00 dB
SFO1 299.8729987 MHz
F2 - Processing parameters
SI 32768
SF 299.8700035 MHz
WDW EM
SSB 0
LB 0.30 Hz
GB 0
PC 1.00
ppm

^{1}H NMR of 6i

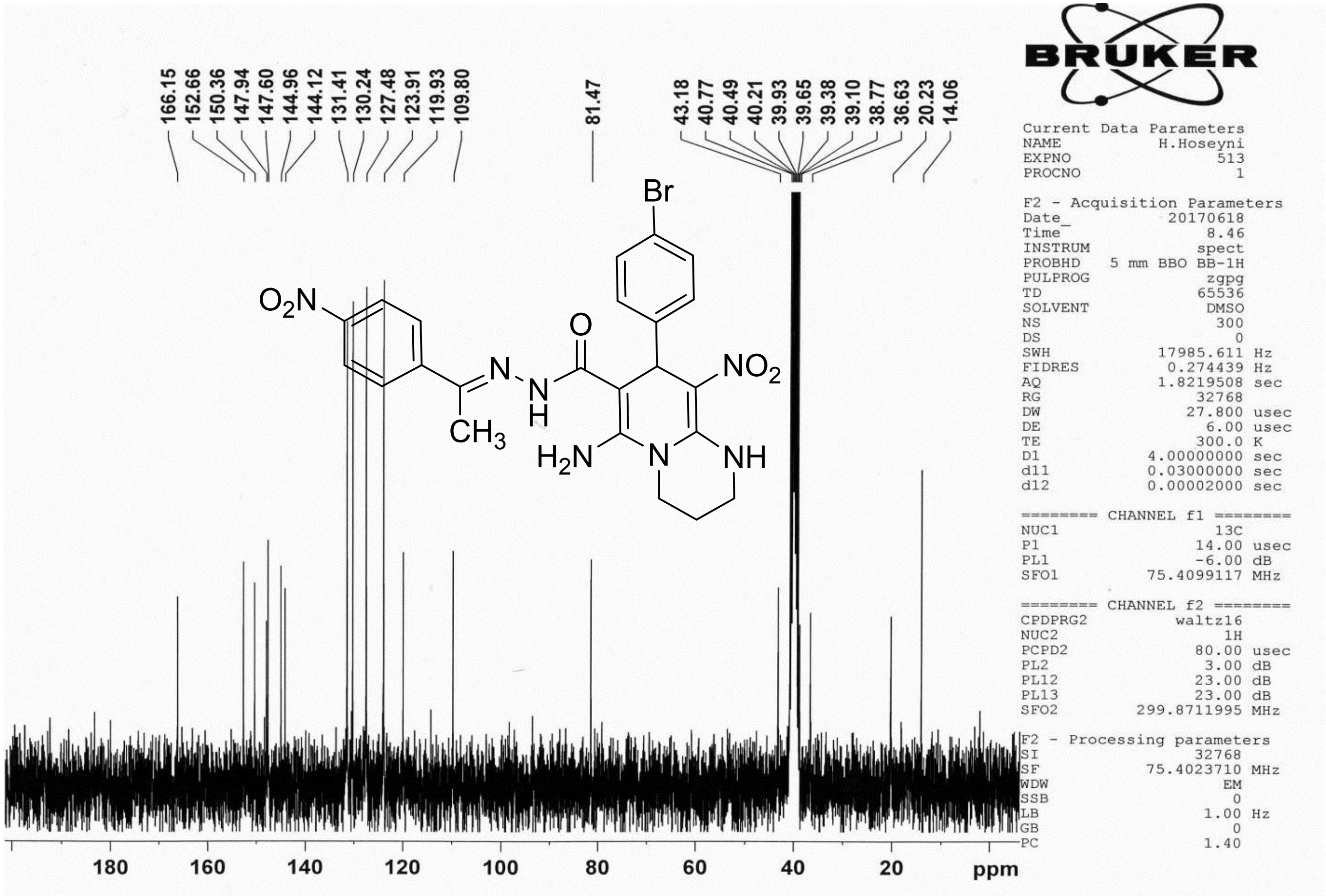
166.15
152.66
150.36
147.94
147.60
144.96
144.12
131.41
130.24
127.48
123.91
119.93
109.80
81.47
43.18
40.77
40.49
40.21
39.93
39.65
39.38
39.10
38.77
36.63
20.23
14.06
BRUKER
Current Data Parameters
NAME H.Hoseyni
EXPNO 513
PROCNO 1
F2 - Acquisition Parameters
Date_ 20170618
Time 8.46
INSTRUM spect
PROBHD 5 mm BBO BB-1H
PULPROG zgpg
TD 65536
SOLVENT DMSO
NS 300
DS 0
SWH 17985.611 Hz
FIDRES 0.274439 Hz
AQ 1.8219508 sec
RG 32768
DW 27.800 usec
DE 6.00 usec
TE 300.0 K
D1 4.00000000 sec
d11 0.03000000 sec
d12 0.00002000 sec
======== CHANNEL f1 ========
NUC1 13C
P1 14.00 usec
PL1 -6.00 dB
SFO1 75.4099117 MHz
======== CHANNEL f2 ========
CPDPRG2 waltz16
NUC2 1H
PCPD2 80.00 usec
PL2 3.00 dB
PL12 23.00 dB
PL13 23.00 dB
SFO2 299.8711995 MHz
F2 - Processing parameters
SI 32768
SF 75.4023710 MHz
WDW EM
SSB 0
LB 1.00 Hz
GB 0
PC 1.40
180 160 140 120 100 80 60 40 20 ppm

^{13}C NMR of 6i

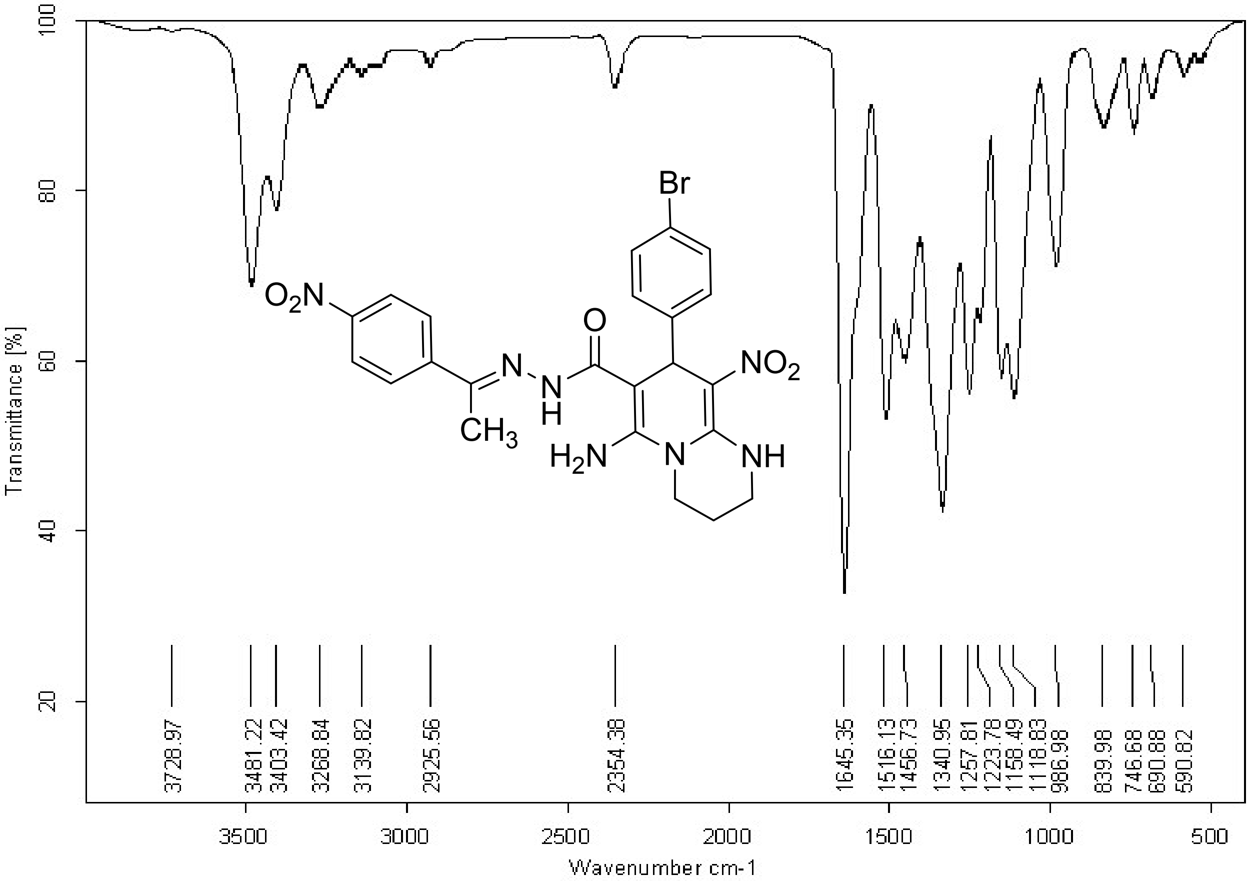
100
80
60
40
20
Transmittance [%]
3728.97
3481.22
3403.42
3268.84
3139.82
2925.56
2354.38
1645.35
1516.13
1456.73
1340.95
1257.81
1223.78
1158.49
1118.83
986.98
839.98
746.68
690.88
590.82
3500
3000
2500
2000
1500
1000
500
Wavenumber cm-1
Br
O_2N
O
N
N
H
NO_2
CH_3
H_2N
N
NH

IR of 6i

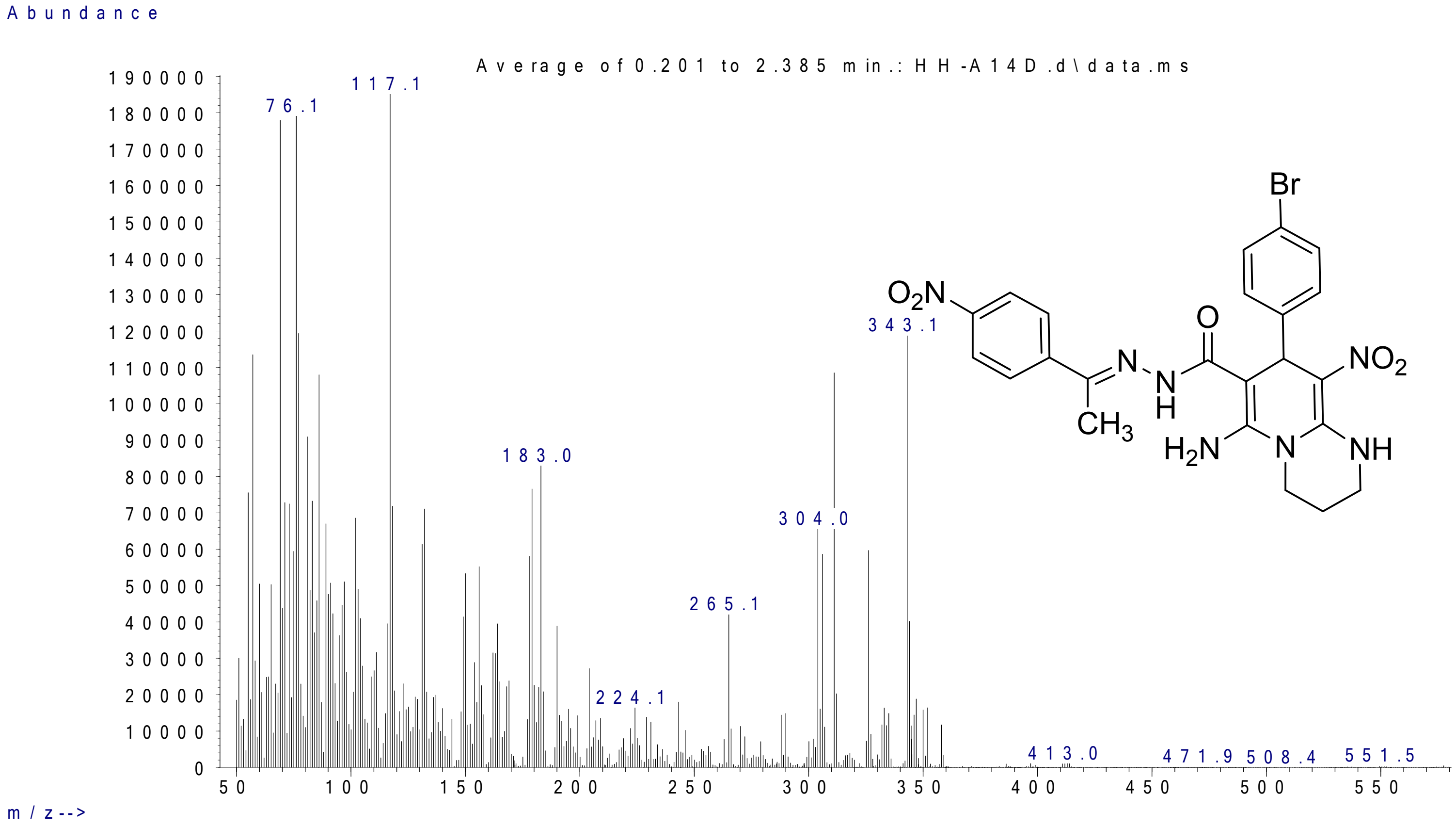
Abundance
Average of 0.201 to 2.385 min.: HH-A14D.d\data.ms
190000
180000
170000
160000
150000
140000
130000
120000
110000
100000
90000
80000
70000
60000
50000
40000
30000
20000
10000
0
76.1
117.1
183.0
224.1
265.1
304.0
343.1
413.0
471.9
508.4
551.5
50 100 150 200 250 300 350 400 450 500 550
m/z-->
Br
O2N
O
NO2
N
N
H
CH3
H2N
N
NH

MS of 6i

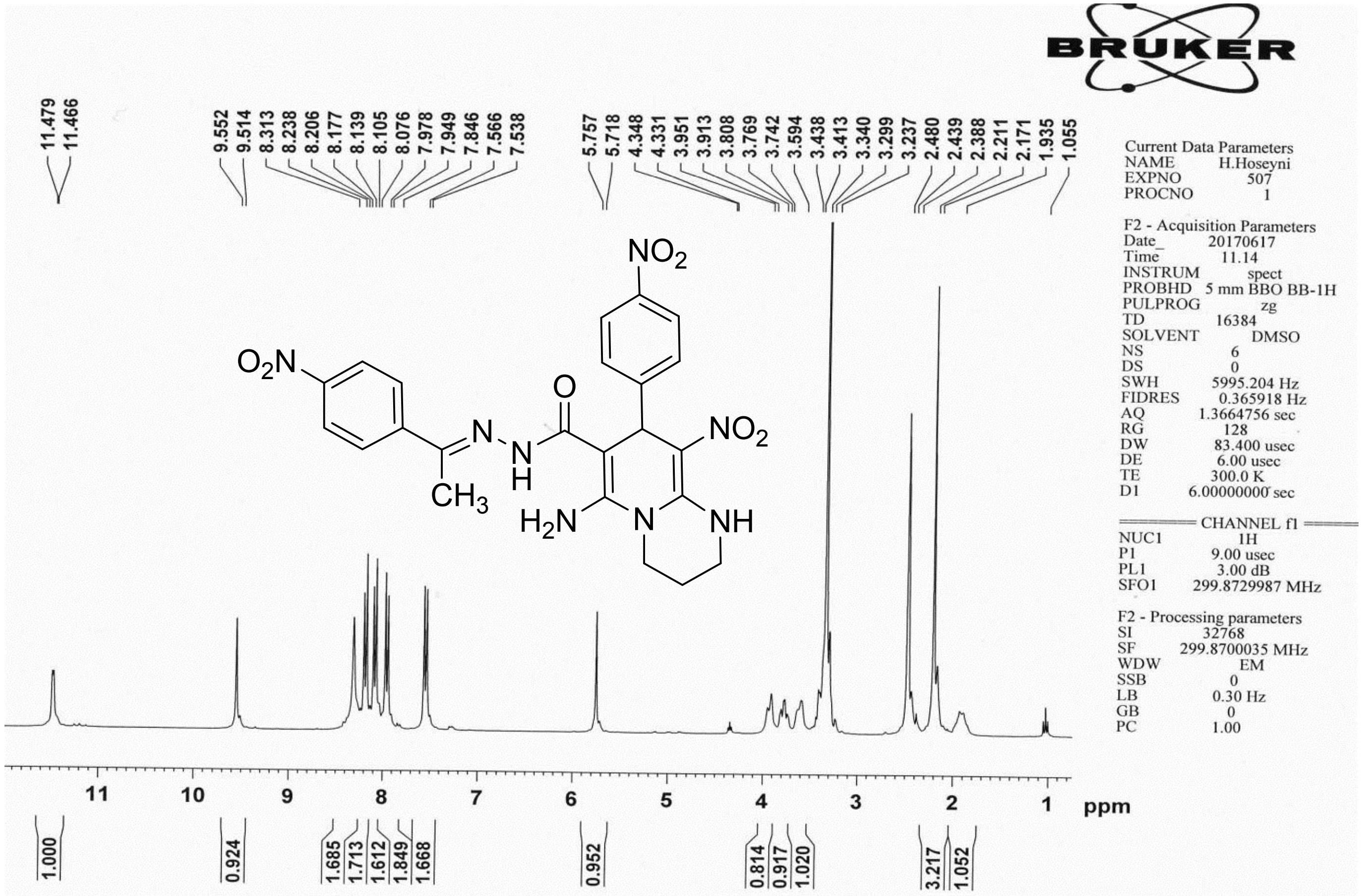
BRUKER
11.479
11.466
9.552
9.514
8.313
8.238
8.206
8.177
8.139
8.105
8.076
7.978
7.949
7.846
7.566
7.538
5.757
5.718
4.348
4.331
3.951
3.913
3.808
3.769
3.742
3.594
3.438
3.413
3.340
3.299
3.237
2.480
2.439
2.388
2.211
2.171
1.935
1.055
Current Data Parameters
NAME H.Hoseyni
EXPNO 507
PROCNO 1
F2 - Acquisition Parameters
Date_ 20170617
Time 11.14
INSTRUM spect
PROBHD 5 mm BBO BB-1H
PULPROG zg
TD 16384
SOLVENT DMSO
NS 6
DS 0
SWH 5995.204 Hz
FIDRES 0.365918 Hz
AQ 1.3664756 sec
RG 128
DW 83.400 usec
DE 6.00 usec
TE 300.0 K
D1 6.00000000 sec
CHANNEL f1
NUC1 1H
P1 9.00 usec
PL1 3.00 dB
SFO1 299.8729987 MHz
F2 - Processing parameters
SI 32768
SF 299.8700035 MHz
WDW EM
SSB 0
LB 0.30 Hz
GB 0
PC 1.00
NO2
O2N
O
N
N
H
NO2
CH3
H2N
N
NH
11
10
9
8
7
6
5
4
3
2
1
ppm
1.000
0.924
1.685
1.713
1.612
1.849
1.668
0.952
0.814
0.917
1.020
3.217
1.052

^{1}H NMR of 6j

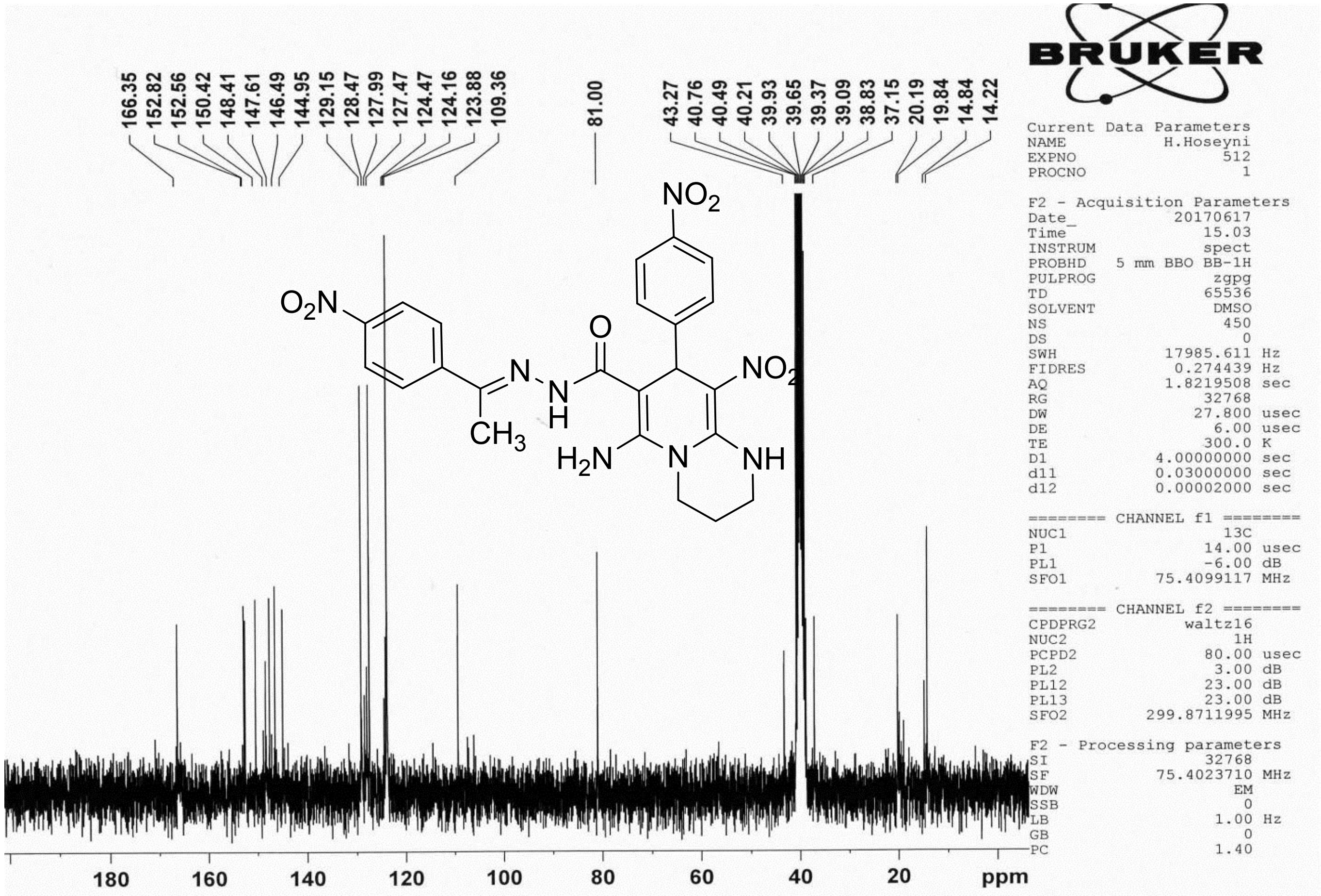

166.35 152.82 152.56 150.42 148.41 147.61 146.49 144.95 129.15 128.47 127.99 127.47 124.47 124.16 123.88 109.36
81.00
43.27 40.76 40.49 40.21 39.93 39.65 39.37 39.09 38.83 37.15 20.19 19.84 14.84 14.22
BRUKER
Current Data Parameters
NAME H.Hoseyni
EXPNO 512
PROCNO 1
F2 - Acquisition Parameters
Date_ 20170617
Time 15.03
INSTRUM spect
PROBHD 5 mm BBO BB-1H
PULPROG zgpg
TD 65536
SOLVENT DMSO
NS 450
DS 0
SWH 17985.611 Hz
FIDRES 0.274439 Hz
AQ 1.8219508 sec
RG 32768
DW 27.800 usec
DE 6.00 usec
TE 300.0 K
D1 4.00000000 sec
d11 0.03000000 sec
d12 0.00002000 sec
======== CHANNEL f1 ========
NUC1 13C
P1 14.00 usec
PL1 -6.00 dB
SFO1 75.4099117 MHz
======== CHANNEL f2 ========
CPDPRG2 waltz16
NUC2 1H
PCPD2 80.00 usec
PL2 3.00 dB
PL12 23.00 dB
PL13 23.00 dB
SFO2 299.8711995 MHz
F2 - Processing parameters
SI 32768
SF 75.4023710 MHz
WDW EM
SSB 0
LB 1.00 Hz
GB 0
PC 1.40
180 160 140 120 100 80 60 40 20 ppm

^{13}C NMR of 6j

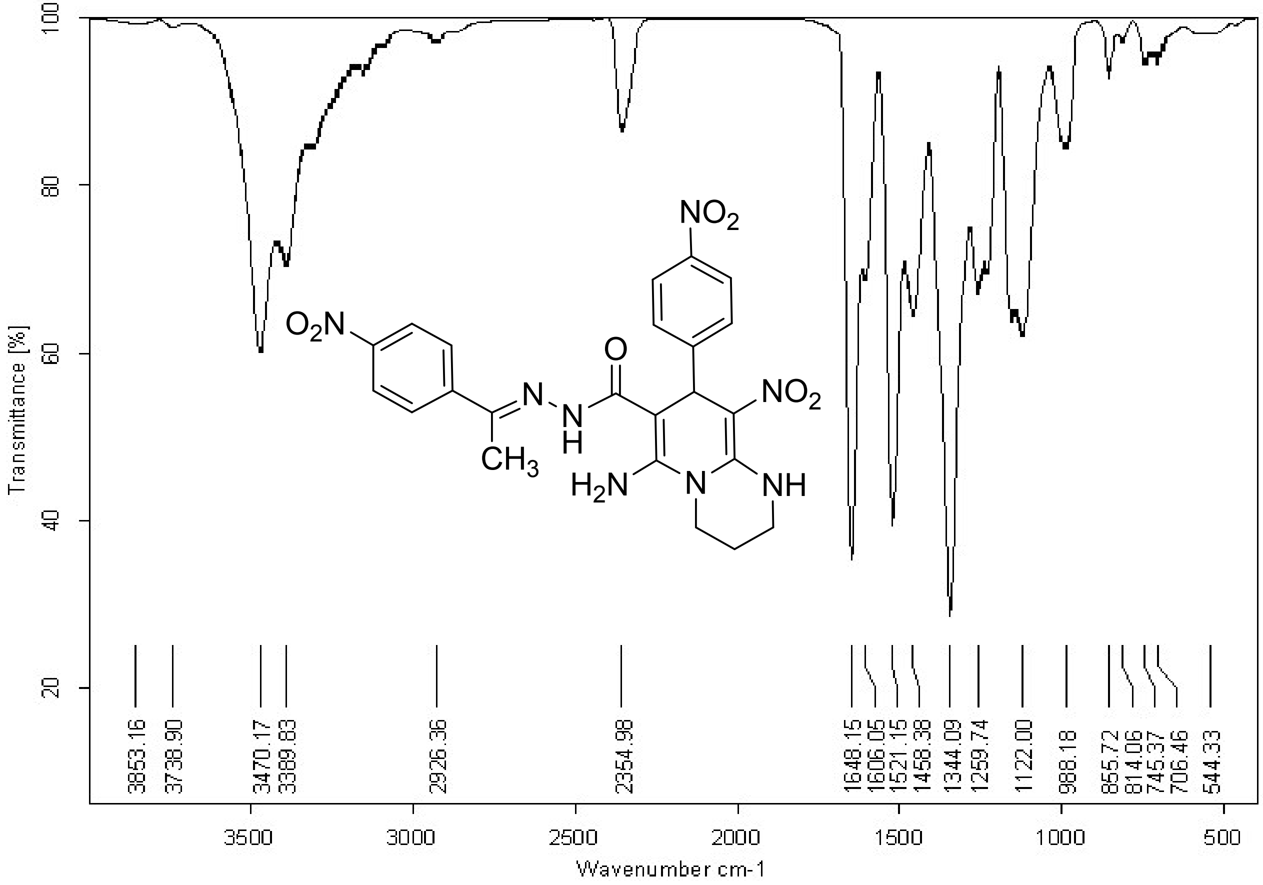

Transmittance [%]
100
80
60
40
20
Wavenumber cm-1
3500
3000
2500
2000
1500
1000
500
3853.16
3738.90
3470.17
3389.83
2926.36
2354.98
1648.15
1606.05
1521.15
1458.38
1344.09
1259.74
1122.00
988.18
855.72
814.06
745.37
706.46
544.33
O2N
NO2
NO2
O
N
N
H
CH3
H2N
N
NH

IR of 6j

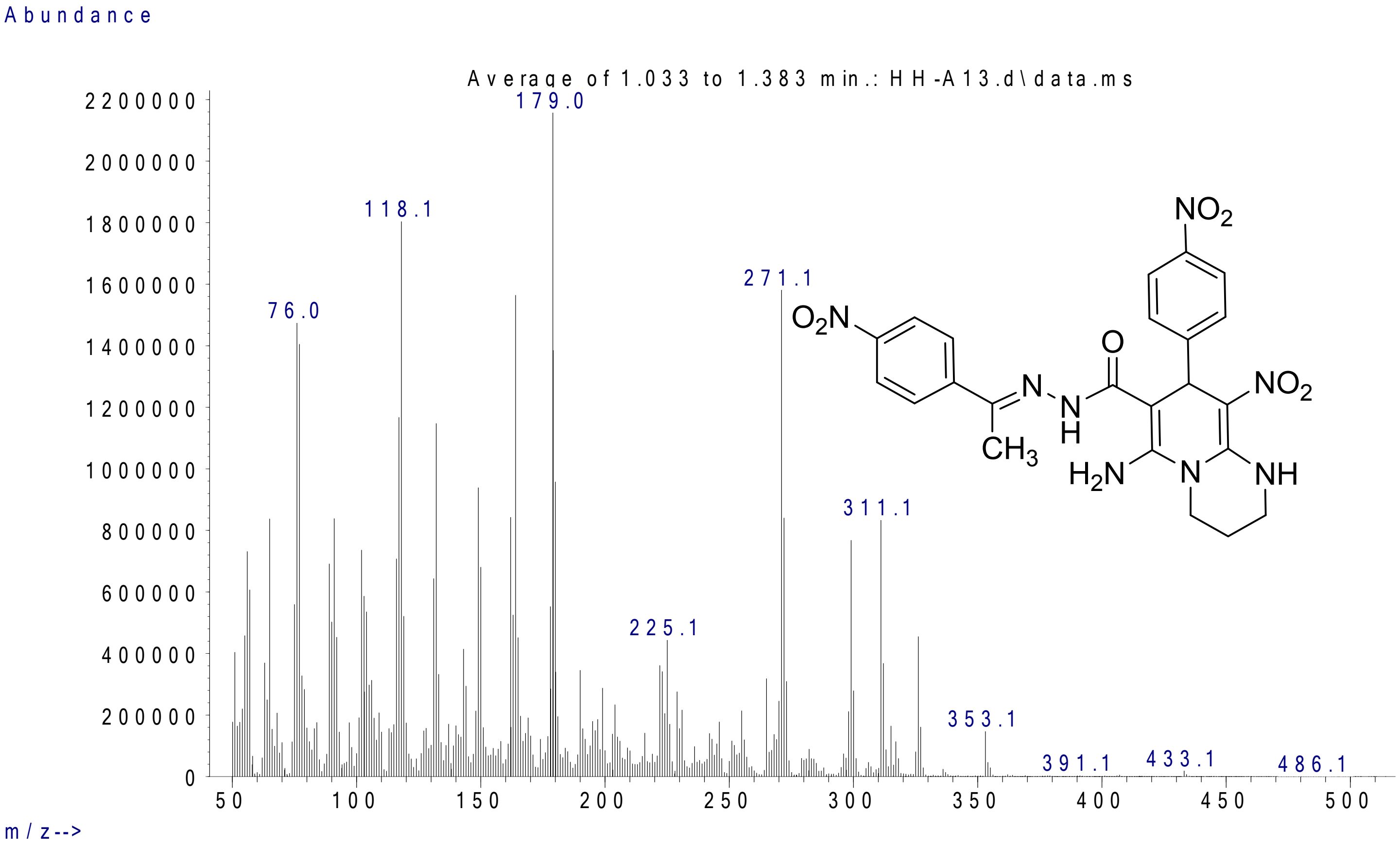
Abundance
Average of 1.033 to 1.383 min.: HH-A13.d\data.ms
2200000
2000000
1800000
1600000
1400000
1200000
1000000
800000
600000
400000
200000
0
179.0
118.1
76.0
271.1
311.1
225.1
353.1
391.1
433.1
486.1
50
100
150
200
250
300
350
400
450
500
m/z-->
NO2
O2N
O
N
N
H
NO2
CH3
H2N
N
NH

MS of 6j

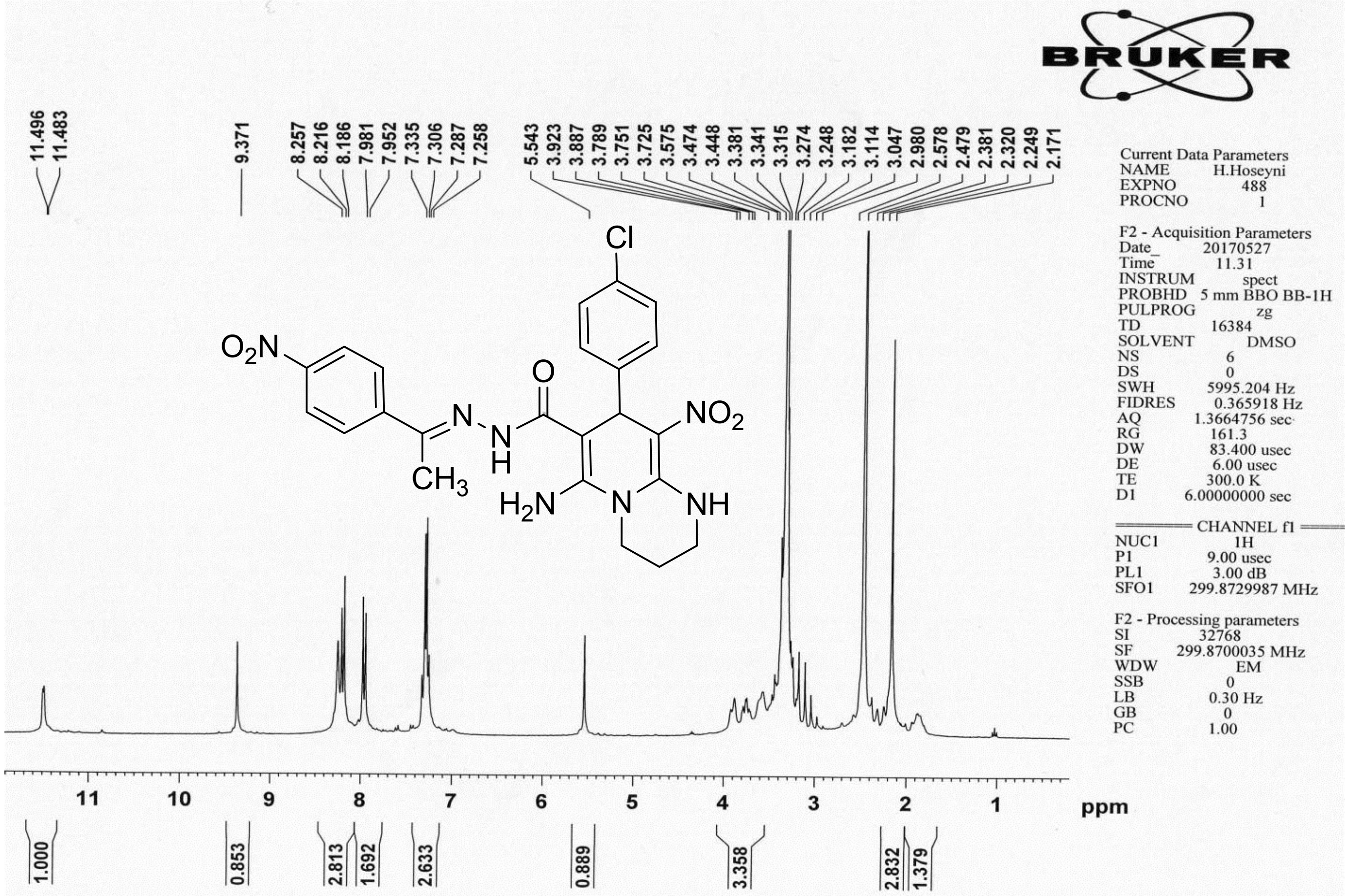

BRUKER
11.496
11.483
9.371
8.257
8.216
8.186
7.981
7.952
7.335
7.306
7.287
7.258
5.543
3.923
3.887
3.789
3.751
3.725
3.575
3.474
3.448
3.381
3.341
3.315
3.274
3.248
3.182
3.114
3.047
2.980
2.578
2.479
2.381
2.320
2.249
2.171
Current Data Parameters
NAME H.Hoseyni
EXPNO 488
PROCNO 1
F2 - Acquisition Parameters
Date_ 20170527
Time 11.31
INSTRUM spect
PROBHD 5 mm BBO BB-1H
PULPROG zg
TD 16384
SOLVENT DMSO
NS 6
DS 0
SWH 5995.204 Hz
FIDRES 0.365918 Hz
AQ 1.3664756 sec
RG 161.3
DW 83.400 usec
DE 6.00 usec
TE 300.0 K
D1 6.00000000 sec
CHANNEL f1
NUC1 1H
P1 9.00 usec
PL1 3.00 dB
SFO1 299.8729987 MHz
F2 - Processing parameters
SI 32768
SF 299.8700035 MHz
WDW EM
SSB 0
LB 0.30 Hz
GB 0
PC 1.00
Cl
O2N
O
N
N
H
NO2
CH3
H2N
N
NH
11
10
9
8
7
6
5
4
3
2
1
ppm
1.000
0.853
2.813
1.692
2.633
0.889
3.358
2.832
1.379

^{1}H NMR of 6k

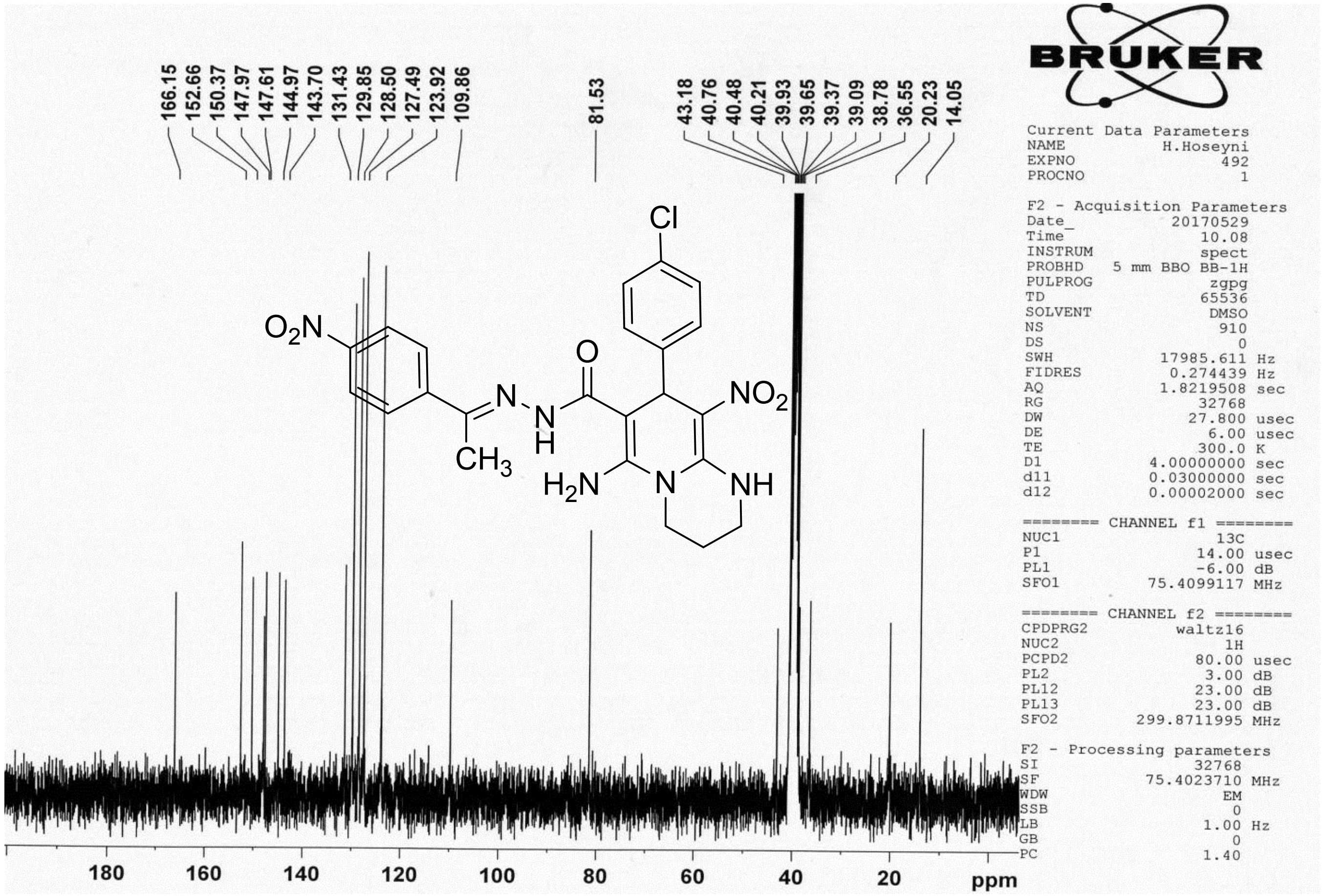
166.15
152.66
150.37
147.97
147.61
144.97
143.70
131.43
129.85
128.50
127.49
123.92
109.86
81.53
43.18
40.76
40.48
40.21
39.93
39.65
39.37
39.09
38.78
36.55
20.23
14.05
BRUKER
Current Data Parameters
NAME H.Hoseyni
EXPNO 492
PROCNO 1
F2 - Acquisition Parameters
Date_ 20170529
Time 10.08
INSTRUM spect
PROBHD 5 mm BBO BB-1H
PULPROG zgpg
TD 65536
SOLVENT DMSO
NS 910
DS 0
SWH 17985.611 Hz
FIDRES 0.274439 Hz
AQ 1.8219508 sec
RG 32768
DW 27.800 usec
DE 6.00 usec
TE 300.0 K
D1 4.00000000 sec
d11 0.03000000 sec
d12 0.00002000 sec
======== CHANNEL f1 ========
NUC1 13C
P1 14.00 usec
PL1 -6.00 dB
SFO1 75.4099117 MHz
======== CHANNEL f2 ========
CPDPRG2 waltz16
NUC2 1H
PCPD2 80.00 usec
PL2 3.00 dB
PL12 23.00 dB
PL13 23.00 dB
SFO2 299.8711995 MHz
F2 - Processing parameters
SI 32768
SF 75.4023710 MHz
WDW EM
SSB 0
LB 1.00 Hz
GB 0
PC 1.40
180
160
140
120
100
80
60
40
20
ppm

^{13}C NMR of 6k

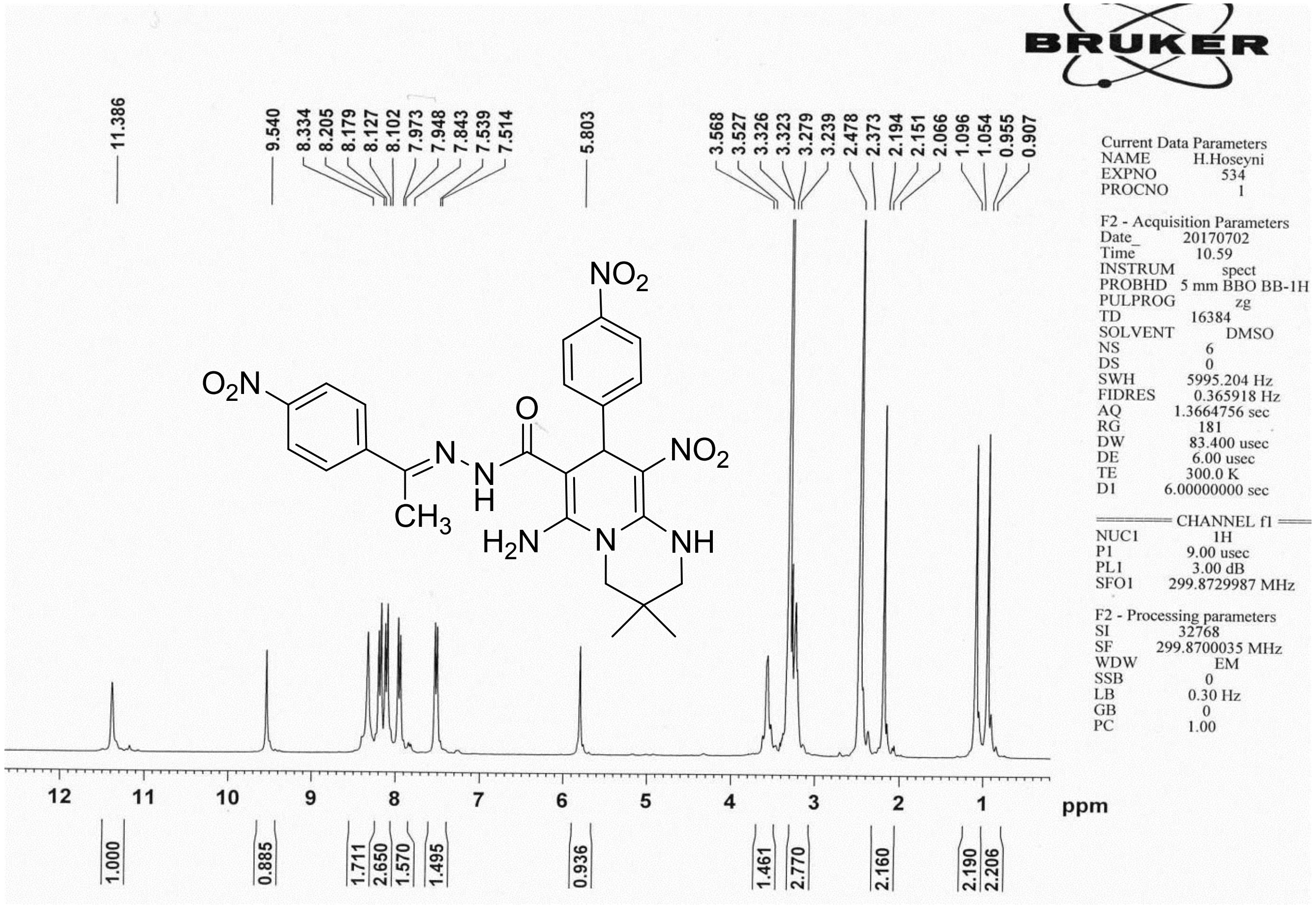
BRUKER
Current Data Parameters
NAME H.Hoseyni
EXPNO 534
PROCNO 1
F2 - Acquisition Parameters
Date_ 20170702
Time 10.59
INSTRUM spect
PROBHD 5 mm BBO BB-1H
PULPROG zg
TD 16384
SOLVENT DMSO
NS 6
DS 0
SWH 5995.204 Hz
FIDRES 0.365918 Hz
AQ 1.3664756 sec
RG 181
DW 83.400 usec
DE 6.00 usec
TE 300.0 K
D1 6.00000000 sec
CHANNEL f1
NUC1 1H
P1 9.00 usec
PL1 3.00 dB
SFO1 299.8729987 MHz
F2 - Processing parameters
SI 32768
SF 299.8700035 MHz
WDW EM
SSB 0
LB 0.30 Hz
GB 0
PC 1.00
ppm

^{1}H NMR of 6l

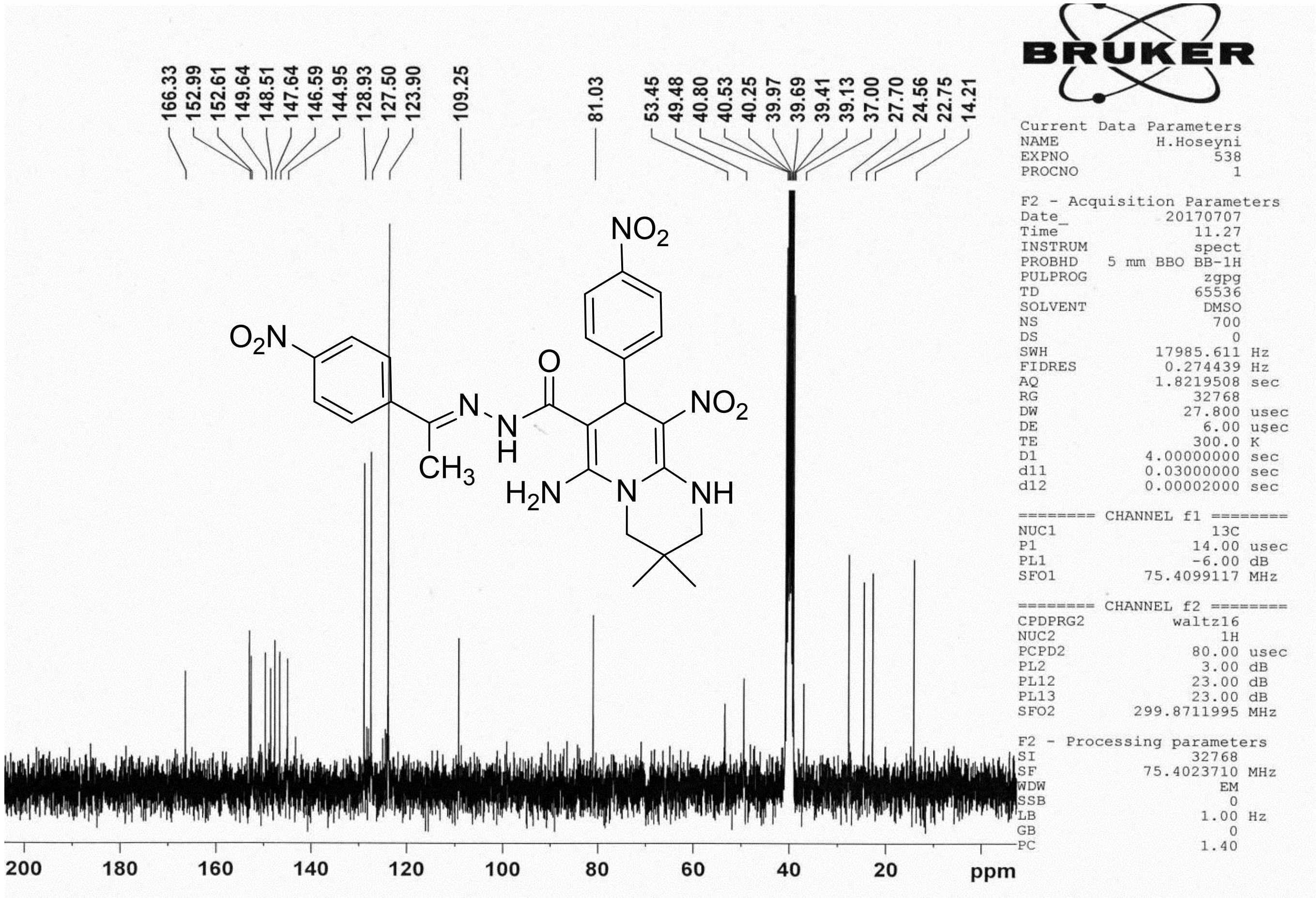
166.33
152.99
152.61
149.64
148.51
147.64
146.59
144.95
128.93
127.50
123.90
109.25
81.03
53.45
49.48
40.80
40.53
40.25
39.97
39.69
39.41
39.13
37.00
27.70
24.56
22.75
14.21
BRUKER
Current Data Parameters
NAME H.Hoseyni
EXPNO 538
PROCNO 1
F2 - Acquisition Parameters
Date_ 20170707
Time 11.27
INSTRUM spect
PROBHD 5 mm BBO BB-1H
PULPROG zgpg
TD 65536
SOLVENT DMSO
NS 700
DS 0
SWH 17985.611 Hz
FIDRES 0.274439 Hz
AQ 1.8219508 sec
RG 32768
DW 27.800 usec
DE 6.00 usec
TE 300.0 K
D1 4.00000000 sec
d11 0.03000000 sec
d12 0.00002000 sec
======== CHANNEL f1 ========
NUC1 13C
P1 14.00 usec
PL1 -6.00 dB
SFO1 75.4099117 MHz
======== CHANNEL f2 ========
CPDPRG2 waltz16
NUC2 1H
PCPD2 80.00 usec
PL2 3.00 dB
PL12 23.00 dB
PL13 23.00 dB
SFO2 299.8711995 MHz
F2 - Processing parameters
SI 32768
SF 75.4023710 MHz
WDW EM
SSB 0
LB 1.00 Hz
GB 0
PC 1.40
NO2
O2N
O
N
N
H
NO2
CH3
H2N
N
NH
200
180
160
140
120
100
80
60
40
20
ppm

^{13}C NMR of 6l

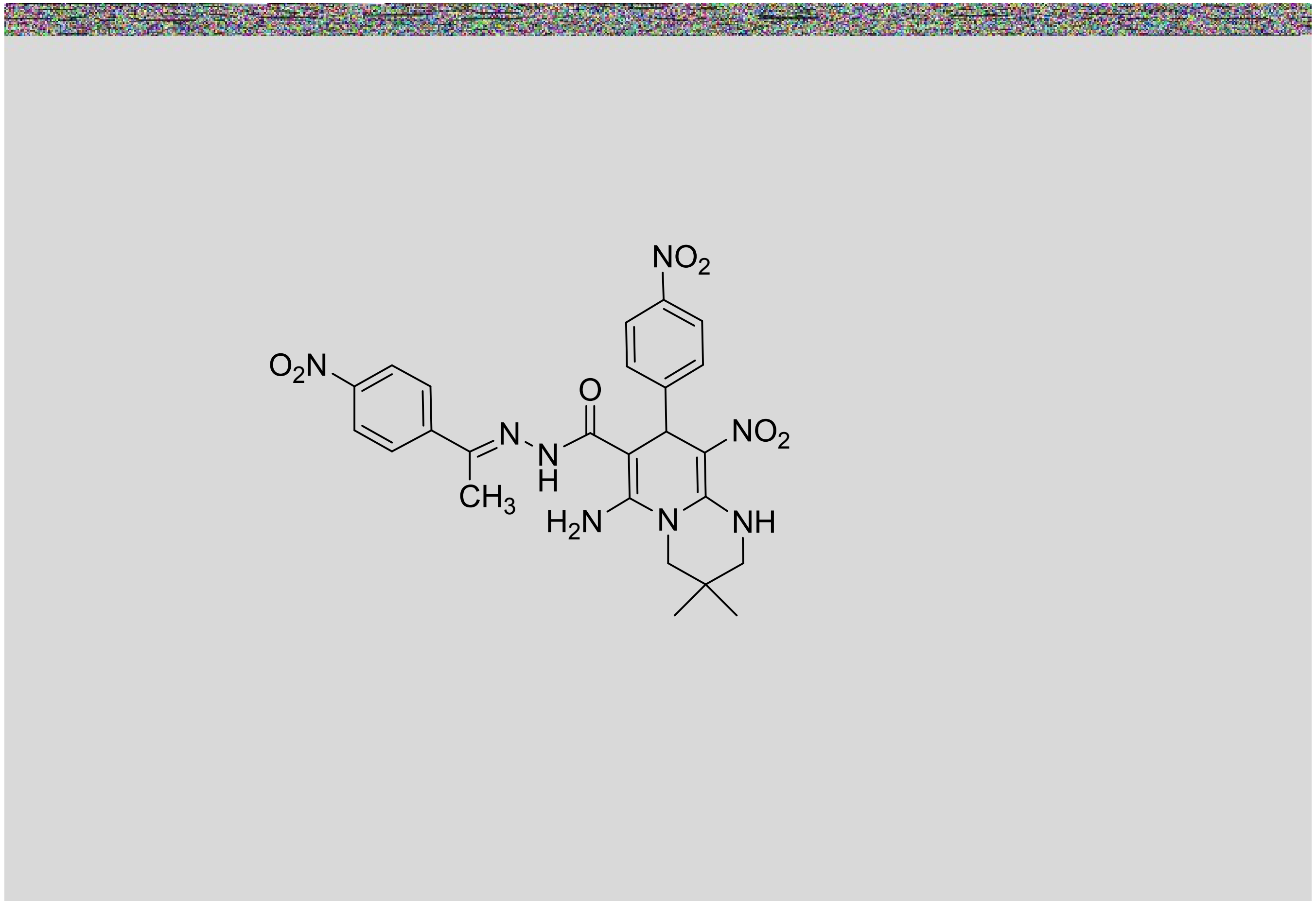

IR of 6l

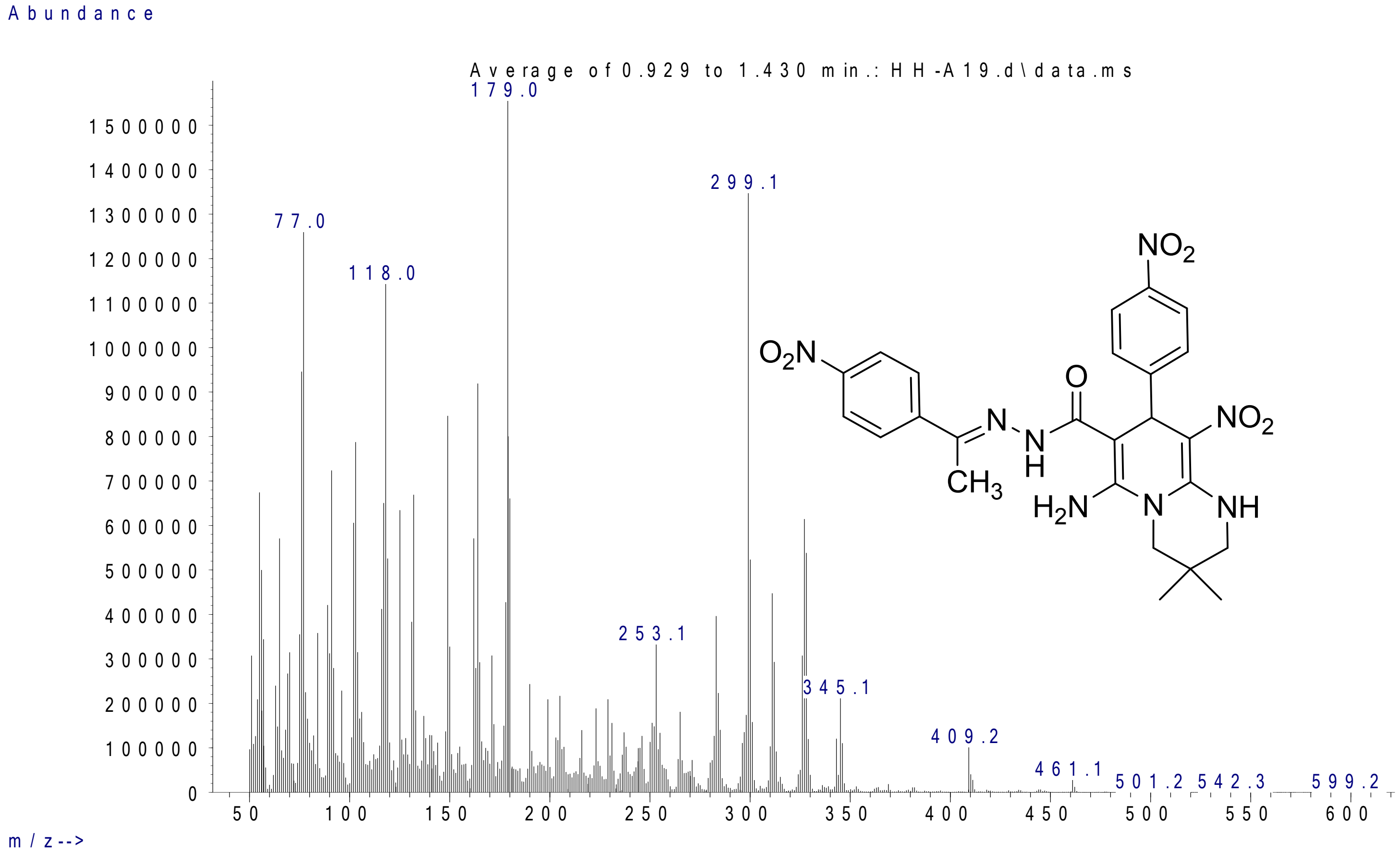
Abundance
Average of 0.929 to 1.430 min.: HH-A19.d\data.ms
179.0
299.1
77.0
118.0
253.1
345.1
409.2
461.1
501.2
542.3
599.2
m/z-->
NO_2
O_2N
O
N
N
H
NO_2
CH_3
H_2N
N
NH

MS of 6l

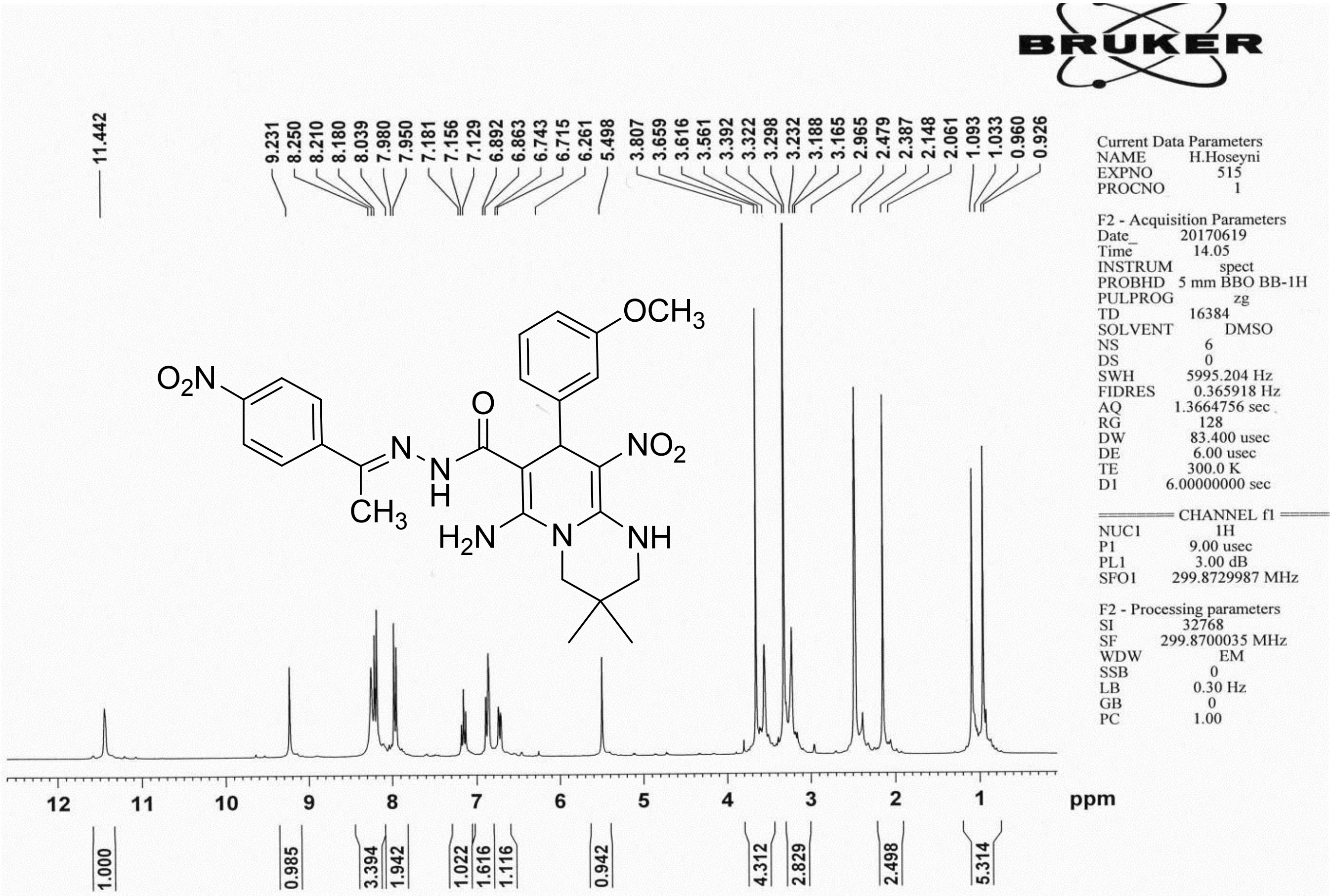
BRUKER
11.442
9.231
8.250
8.210
8.180
8.039
7.980
7.950
7.181
7.156
7.129
6.892
6.863
6.743
6.715
6.261
5.498
3.807
3.659
3.616
3.561
3.392
3.322
3.298
3.232
3.188
3.165
2.965
2.479
2.387
2.148
2.061
1.093
1.033
0.960
0.926
Current Data Parameters
NAME H.Hoseyni
EXPNO 515
PROCNO 1
F2 - Acquisition Parameters
Date_ 20170619
Time 14.05
INSTRUM spect
PROBHD 5 mm BBO BB-1H
PULPROG zg
TD 16384
SOLVENT DMSO
NS 6
DS 0
SWH 5995.204 Hz
FIDRES 0.365918 Hz
AQ 1.3664756 sec
RG 128
DW 83.400 usec
DE 6.00 usec
TE 300.0 K
D1 6.00000000 sec
CHANNEL f1
NUC1 1H
P1 9.00 usec
PL1 3.00 dB
SFO1 299.8729987 MHz
F2 - Processing parameters
SI 32768
SF 299.8700035 MHz
WDW EM
SSB 0
LB 0.30 Hz
GB 0
PC 1.00
OCH3
O2N
O
N
N
H
NO2
CH3
H2N
N
NH
ppm
12
11
10
9
8
7
6
5
4
3
2
1
1.000
0.985
3.394
1.942
1.022
1.616
1.116
0.942
4.312
2.829
2.498
5.314

^{1}H NMR of 6m

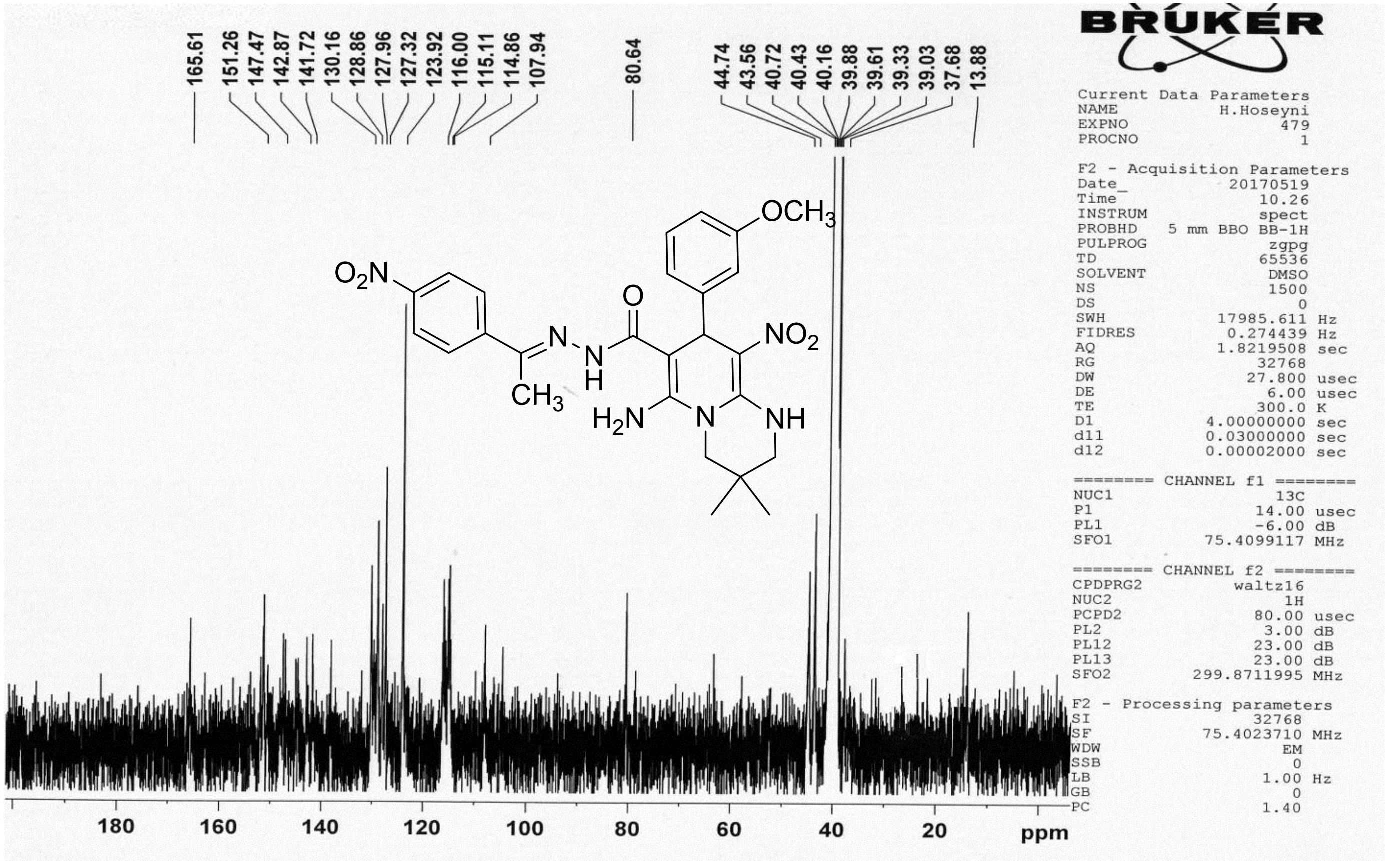
165.61
151.26
147.47
142.87
141.72
130.16
128.86
127.96
127.32
123.92
116.00
115.11
114.86
107.94
80.64
44.74
43.56
40.72
40.43
40.16
39.88
39.61
39.33
39.03
37.68
13.88
BRUKER
Current Data Parameters
NAME H.Hoseyni
EXPNO 479
PROCNO 1
F2 - Acquisition Parameters
Date_ 20170519
Time 10.26
INSTRUM spect
PROBHD 5 mm BBO BB-1H
PULPROG zgpg
TD 65536
SOLVENT DMSO
NS 1500
DS 0
SWH 17985.611 Hz
FIDRES 0.274439 Hz
AQ 1.8219508 sec
RG 32768
DW 27.800 usec
DE 6.00 usec
TE 300.0 K
D1 4.00000000 sec
d11 0.03000000 sec
d12 0.00002000 sec
======== CHANNEL f1 ========
NUC1 13C
P1 14.00 usec
PL1 -6.00 dB
SFO1 75.4099117 MHz
======== CHANNEL f2 ========
CPDPRG2 waltz16
NUC2 1H
PCPD2 80.00 usec
PL2 3.00 dB
PL12 23.00 dB
PL13 23.00 dB
SFO2 299.8711995 MHz
F2 - Processing parameters
SI 32768
SF 75.4023710 MHz
WDW EM
SSB 0
LB 1.00 Hz
GB 0
PC 1.40
180
160
140
120
100
80
60
40
20
ppm

^{13}C NMR of 6m

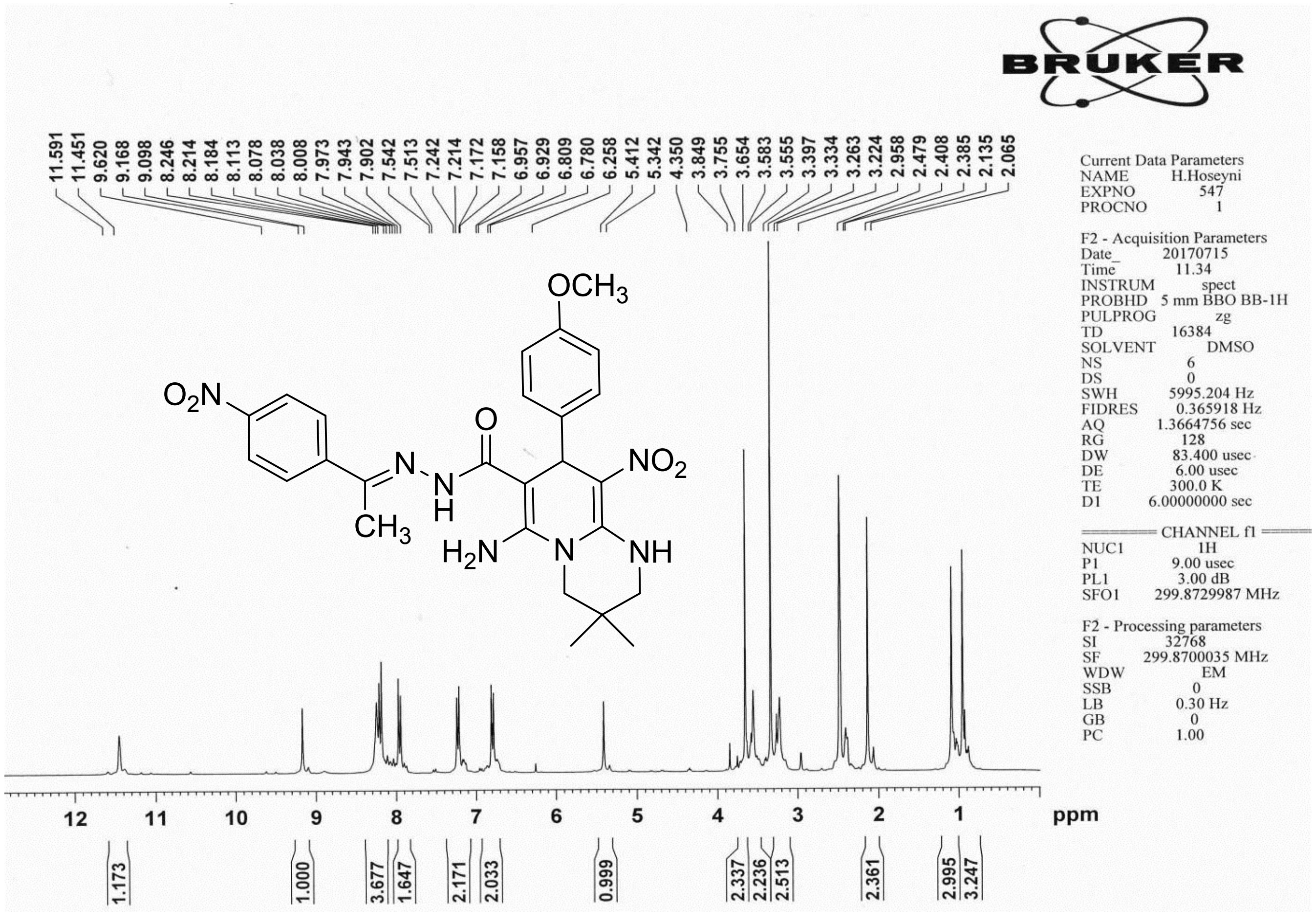
11.591 11.451 9.620 9.168 9.098 8.246 8.214 8.184 8.113 8.078 8.038 8.008 7.973 7.943 7.902 7.542 7.513 7.242 7.214 7.172 7.158 6.957 6.929 6.809 6.780 6.258 5.412 5.342 4.350 3.849 3.755 3.654 3.583 3.555 3.397 3.334 3.263 3.224 2.958 2.479 2.408 2.385 2.135 2.065
OCH3
O2N
O
N
N
H
NO2
CH3
H2N
N
NH
Current Data Parameters
NAME H.Hoseyni
EXPNO 547
PROCNO 1
F2 - Acquisition Parameters
Date_ 20170715
Time 11.34
INSTRUM spect
PROBHD 5 mm BBO BB-1H
PULPROG zg
TD 16384
SOLVENT DMSO
NS 6
DS 0
SWH 5995.204 Hz
FIDRES 0.365918 Hz
AQ 1.3664756 sec
RG 128
DW 83.400 usec
DE 6.00 usec
TE 300.0 K
D1 6.00000000 sec
CHANNEL f1
NUC1 1H
P1 9.00 usec
PL1 3.00 dB
SFO1 299.8729987 MHz
F2 - Processing parameters
SI 32768
SF 299.8700035 MHz
WDW EM
SSB 0
LB 0.30 Hz
GB 0
PC 1.00
12 11 10 9 8 7 6 5 4 3 2 1 ppm
1.173 1.000 3.677 1.647 2.171 2.033 0.999 2.337 2.236 2.513 2.361 2.995 3.247

^{1}H NMR of 6n

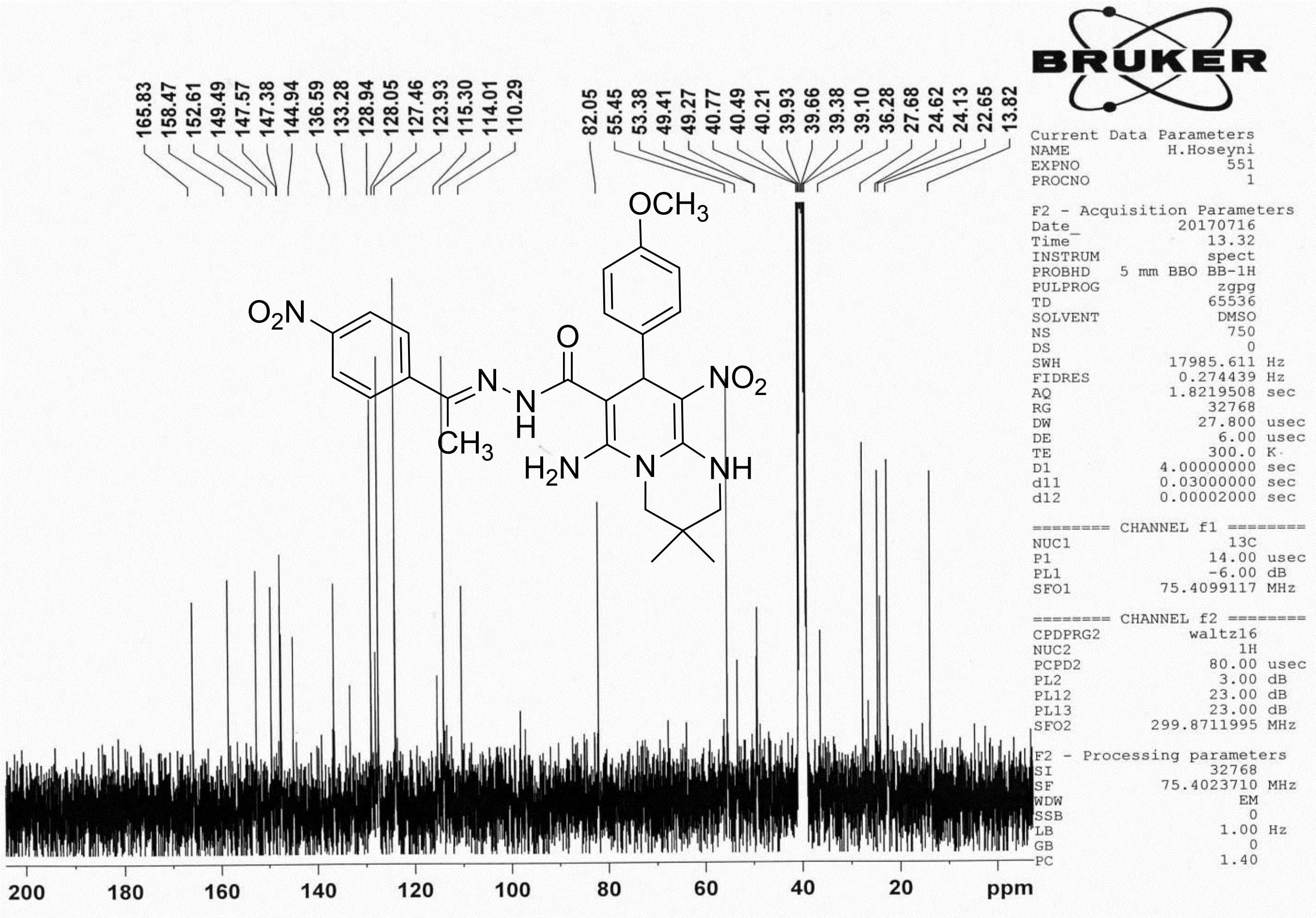
165.83
158.47
152.61
149.49
147.57
147.38
144.94
136.59
133.28
128.94
128.05
127.46
123.93
115.30
114.01
110.29
82.05
55.45
53.38
49.41
49.27
40.77
40.49
40.21
39.93
39.66
39.38
39.10
36.28
27.68
24.62
24.13
22.65
13.82
BRUKER
OCH3
O2N
O
N
N
H
NO2
CH3
H2N
N
NH
Current Data Parameters
NAME H.Hoseyni
EXPNO 551
PROCNO 1
F2 - Acquisition Parameters
Date_ 20170716
Time 13.32
INSTRUM spect
PROBHD 5 mm BBO BB-1H
PULPROG zgpg
TD 65536
SOLVENT DMSO
NS 750
DS 0
SWH 17985.611 Hz
FIDRES 0.274439 Hz
AQ 1.8219508 sec
RG 32768
DW 27.800 usec
DE 6.00 usec
TE 300.0 K
D1 4.00000000 sec
d11 0.03000000 sec
d12 0.00002000 sec
======== CHANNEL f1 ========
NUC1 13C
P1 14.00 usec
PL1 -6.00 dB
SFO1 75.4099117 MHz
======== CHANNEL f2 ========
CPDPRG2 waltz16
NUC2 1H
PCPD2 80.00 usec
PL2 3.00 dB
PL12 23.00 dB
PL13 23.00 dB
SFO2 299.8711995 MHz
F2 - Processing parameters
SI 32768
SF 75.4023710 MHz
WDW EM
SSB 0
LB 1.00 Hz
GB 0
PC 1.40
200 180 160 140 120 100 80 60 40 20 ppm

^{13}C NMR of 6n

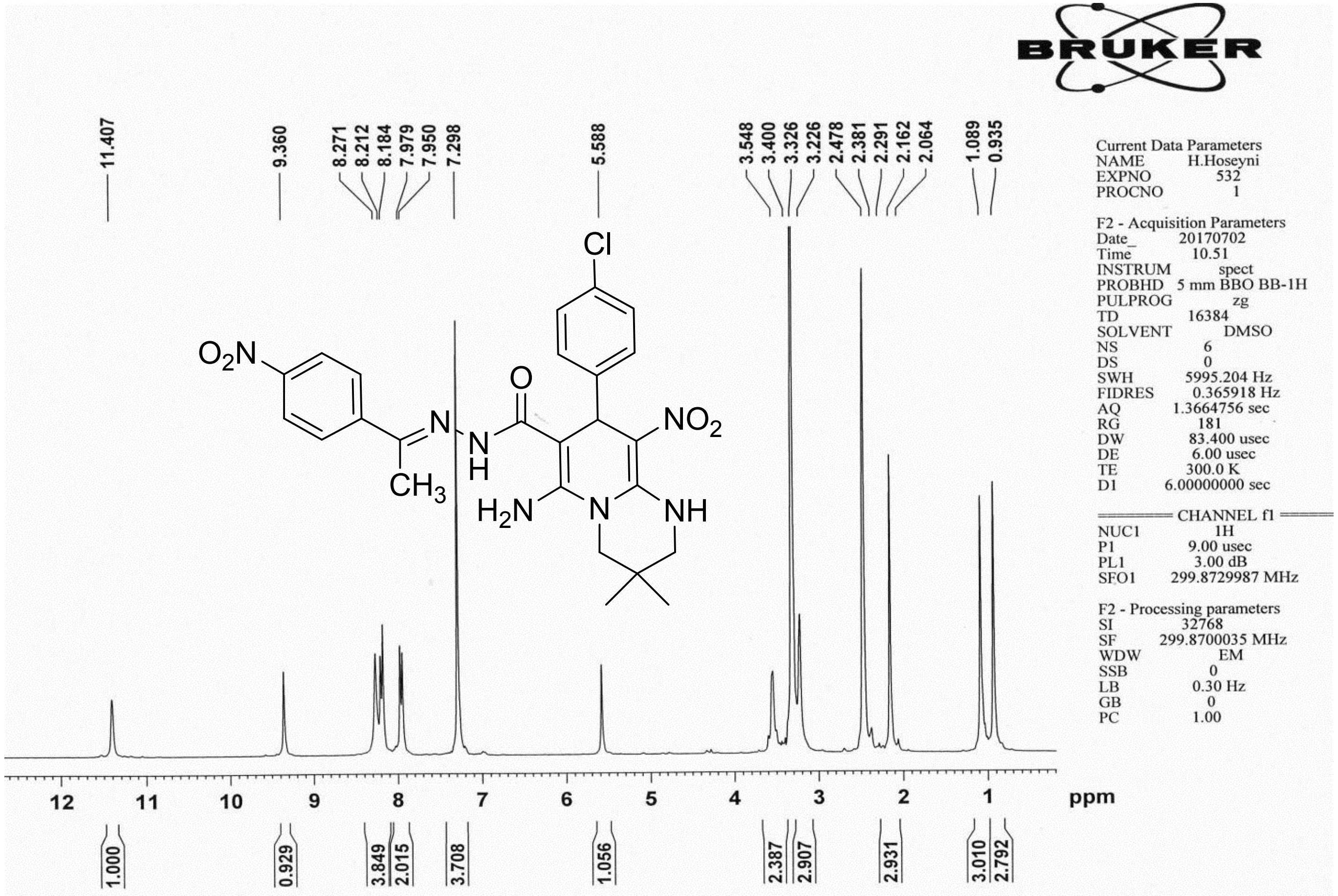
BRUKER
11.407
9.360
8.271
8.212
8.184
7.979
7.950
7.298
5.588
3.548
3.400
3.326
3.226
2.478
2.381
2.291
2.162
2.064
1.089
0.935
Current Data Parameters
NAME H.Hoseyni
EXPNO 532
PROCNO 1
F2 - Acquisition Parameters
Date_ 20170702
Time 10.51
INSTRUM spect
PROBHD 5 mm BBO BB-1H
PULPROG zg
TD 16384
SOLVENT DMSO
NS 6
DS 0
SWH 5995.204 Hz
FIDRES 0.365918 Hz
AQ 1.3664756 sec
RG 181
DW 83.400 usec
DE 6.00 usec
TE 300.0 K
D1 6.00000000 sec
CHANNEL f1
NUC1 1H
P1 9.00 usec
PL1 3.00 dB
SFO1 299.8729987 MHz
F2 - Processing parameters
SI 32768
SF 299.8700035 MHz
WDW EM
SSB 0
LB 0.30 Hz
GB 0
PC 1.00
Cl
O
O2N
N
N
H
NO2
CH3
H2N
N
NH
12
11
10
9
8
7
6
5
4
3
2
1
ppm
1.000
0.929
3.849
2.015
3.708
1.056
2.387
2.907
2.931
3.010
2.792

^{1}H NMR of 6o

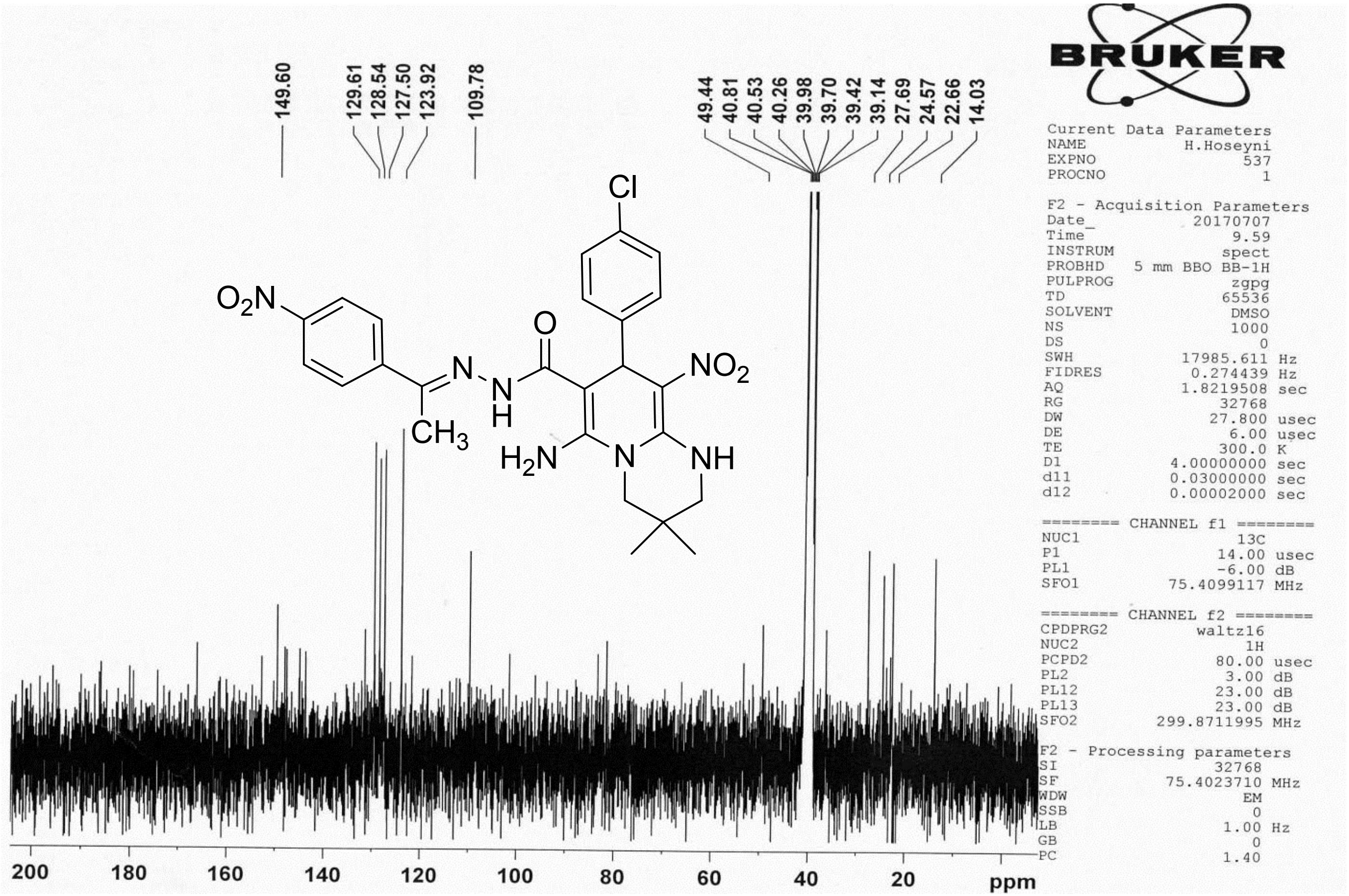
149.60
129.61
128.54
127.50
123.92
109.78
49.44
40.81
40.53
40.26
39.98
39.70
39.42
39.14
27.69
24.57
22.66
14.03
BRUKER
Current Data Parameters
NAME H.Hoseyni
EXPNO 537
PROCNO 1
F2 - Acquisition Parameters
Date_ 20170707
Time 9.59
INSTRUM spect
PROBHD 5 mm BBO BB-1H
PULPROG zgpg
TD 65536
SOLVENT DMSO
NS 1000
DS 0
SWH 17985.611 Hz
FIDRES 0.274439 Hz
AQ 1.8219508 sec
RG 32768
DW 27.800 usec
DE 6.00 usec
TE 300.0 K
D1 4.00000000 sec
d11 0.03000000 sec
d12 0.00002000 sec
======== CHANNEL f1 ========
NUC1 13C
P1 14.00 usec
PL1 -6.00 dB
SFO1 75.4099117 MHz
======== CHANNEL f2 ========
CPDPRG2 waltz16
NUC2 1H
PCPD2 80.00 usec
PL2 3.00 dB
PL12 23.00 dB
PL13 23.00 dB
SFO2 299.8711995 MHz
F2 - Processing parameters
SI 32768
SF 75.4023710 MHz
WDW EM
SSB 0
LB 1.00 Hz
GB 0
PC 1.40
200 180 160 140 120 100 80 60 40 20 ppm

^{13}C NMR of 6o

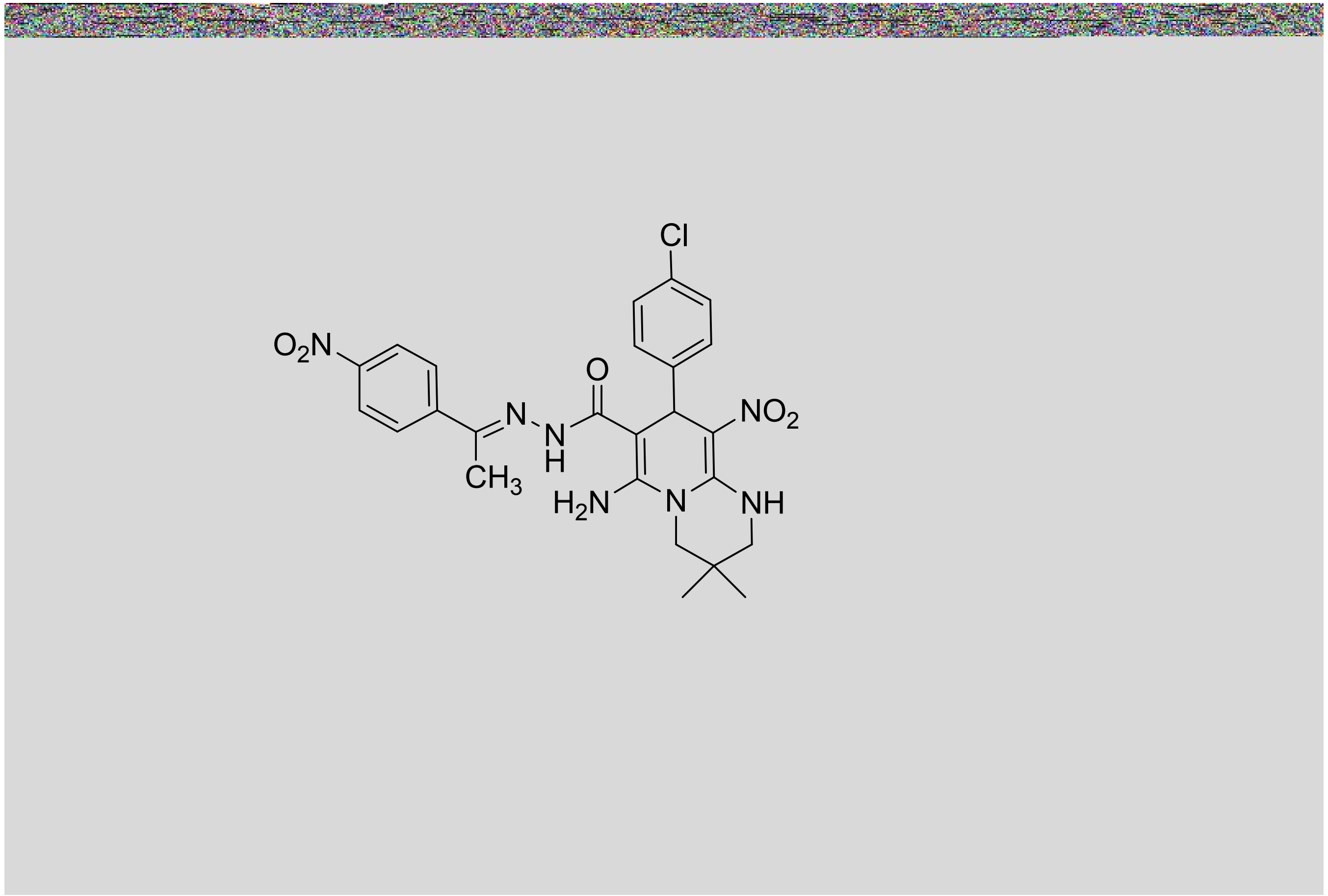

IR of 6o

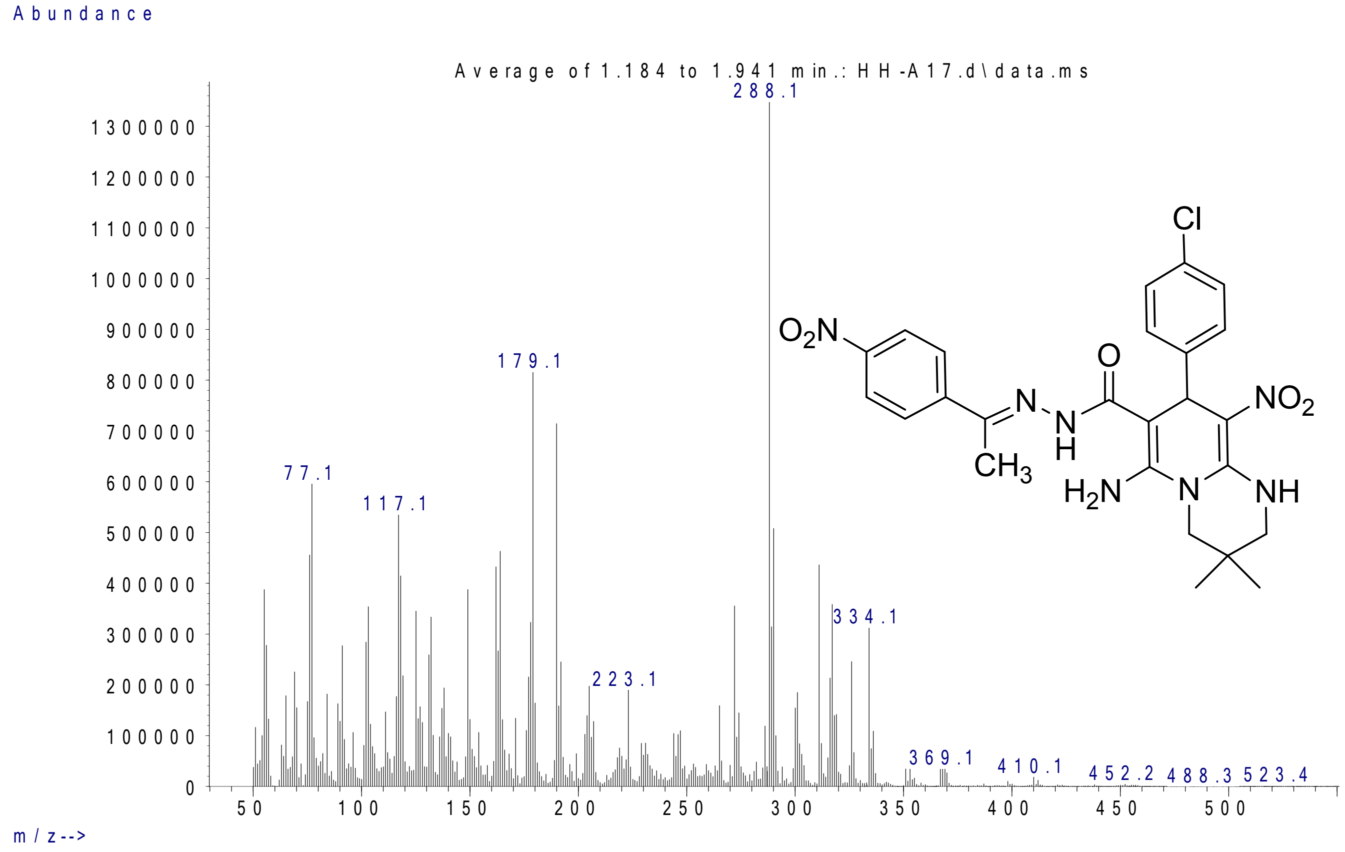
Abundance
Average of 1.184 to 1.941 min.: HH-A17.d\data.ms
288.1
179.1
77.1
117.1
223.1
334.1
369.1
410.1
452.2
488.3
523.4
1300000
1200000
1100000
1000000
900000
800000
700000
600000
500000
400000
300000
200000
100000
0
50
100
150
200
250
300
350
400
450
500
m/z-->
Cl
O2N
O
NO2
N
N
H
CH3
H2N
N
NH

MS of 6o

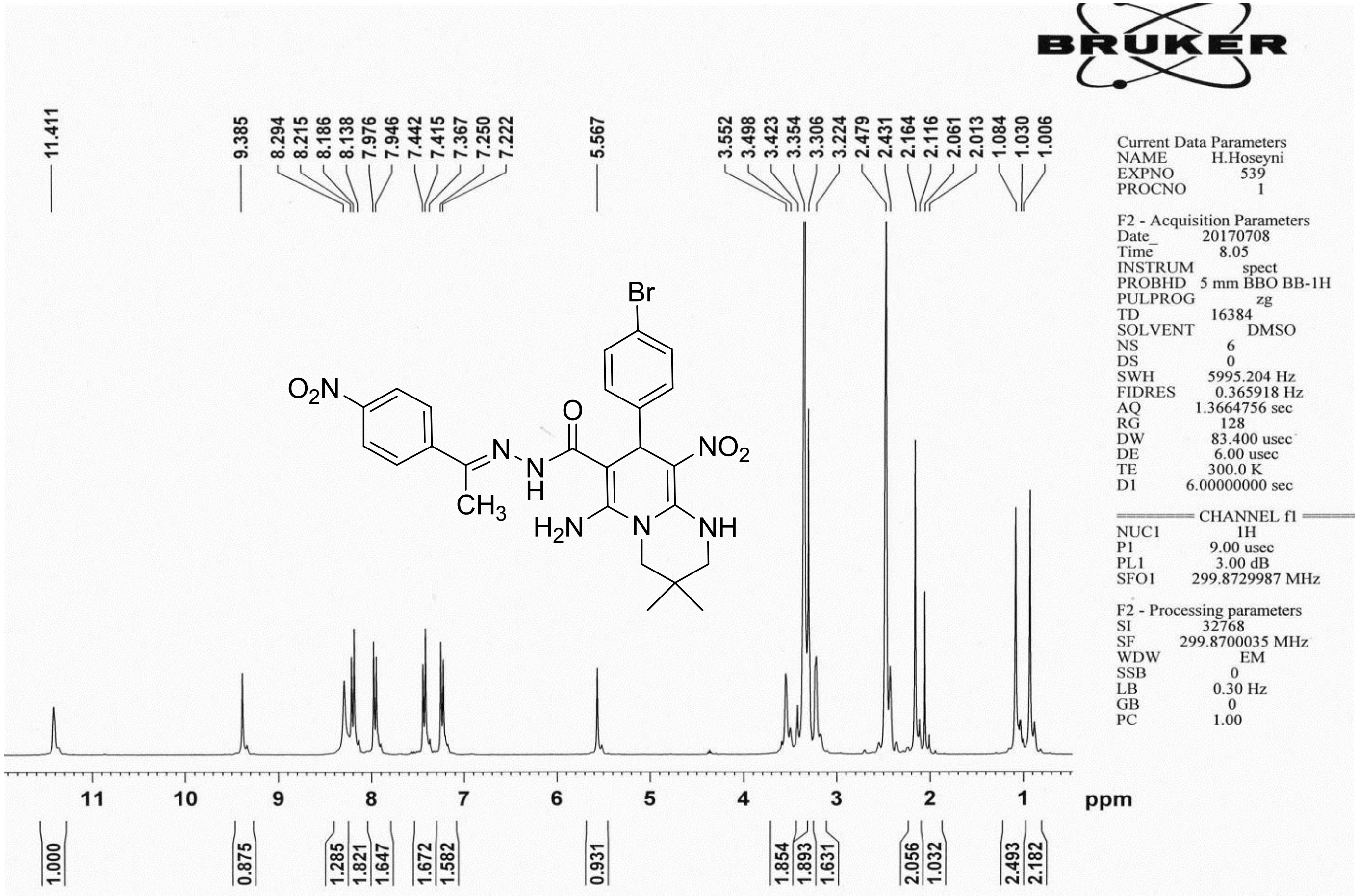
BRUKER
11.411
9.385
8.294
8.215
8.186
8.138
7.976
7.946
7.442
7.415
7.367
7.250
7.222
5.567
3.552
3.498
3.423
3.354
3.306
3.224
2.479
2.431
2.164
2.116
2.061
2.013
1.084
1.030
1.006
Current Data Parameters
NAME H.Hoseyni
EXPNO 539
PROCNO 1
F2 - Acquisition Parameters
Date_ 20170708
Time 8.05
INSTRUM spect
PROBHD 5 mm BBO BB-1H
PULPROG zg
TD 16384
SOLVENT DMSO
NS 6
DS 0
SWH 5995.204 Hz
FIDRES 0.365918 Hz
AQ 1.3664756 sec
RG 128
DW 83.400 usec
DE 6.00 usec
TE 300.0 K
D1 6.00000000 sec
CHANNEL f1
NUC1 1H
P1 9.00 usec
PL1 3.00 dB
SFO1 299.8729987 MHz
F2 - Processing parameters
SI 32768
SF 299.8700035 MHz
WDW EM
SSB 0
LB 0.30 Hz
GB 0
PC 1.00
Br
O2N
O
N
N
H
NO2
CH3
H2N
N
NH
11
10
9
8
7
6
5
4
3
2
1
ppm
1.000
0.875
1.285
1.821
1.647
1.672
1.582
0.931
1.854
1.893
1.631
2.056
1.032
2.493
2.182

^{1}H NMR of 6p

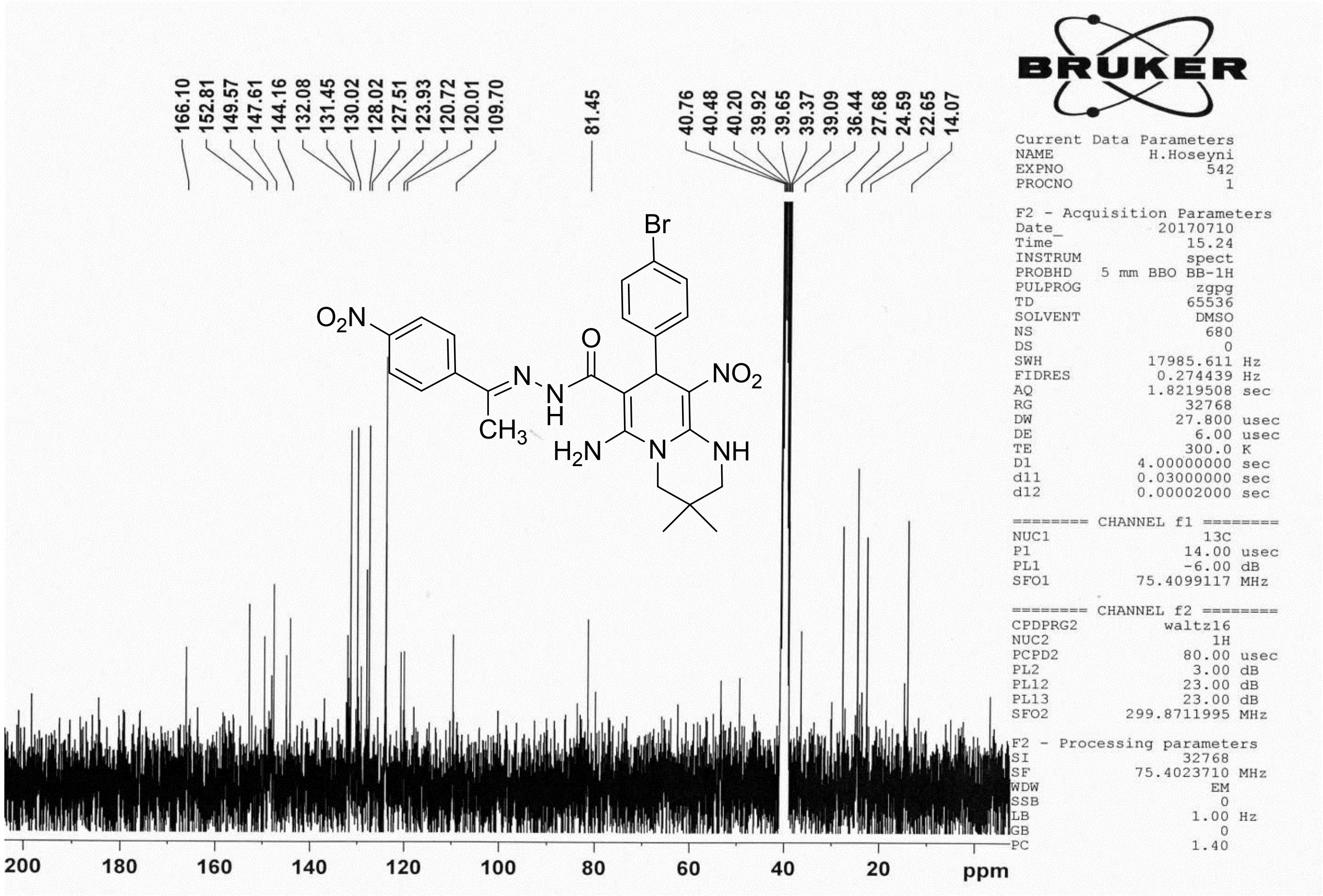
166.10
152.81
149.57
147.61
144.16
132.08
131.45
130.02
128.02
127.51
123.93
120.72
120.01
109.70
81.45
40.76
40.48
40.20
39.92
39.65
39.37
39.09
36.44
27.68
24.59
22.65
14.07
BRUKER
Current Data Parameters
NAME H.Hoseyni
EXPNO 542
PROCNO 1
F2 - Acquisition Parameters
Date_ 20170710
Time 15.24
INSTRUM spect
PROBHD 5 mm BBO BB-1H
PULPROG zgpg
TD 65536
SOLVENT DMSO
NS 680
DS 0
SWH 17985.611 Hz
FIDRES 0.274439 Hz
AQ 1.8219508 sec
RG 32768
DW 27.800 usec
DE 6.00 usec
TE 300.0 K
D1 4.00000000 sec
d11 0.03000000 sec
d12 0.00002000 sec
======== CHANNEL f1 ========
NUC1 13C
P1 14.00 usec
PL1 -6.00 dB
SFO1 75.4099117 MHz
======== CHANNEL f2 ========
CPDPRG2 waltz16
NUC2 1H
PCPD2 80.00 usec
PL2 3.00 dB
PL12 23.00 dB
PL13 23.00 dB
SFO2 299.8711995 MHz
F2 - Processing parameters
SI 32768
SF 75.4023710 MHz
WDW EM
SSB 0
LB 1.00 Hz
GB 0
PC 1.40
200 180 160 140 120 100 80 60 40 20 ppm

^{13}C NMR of 6p

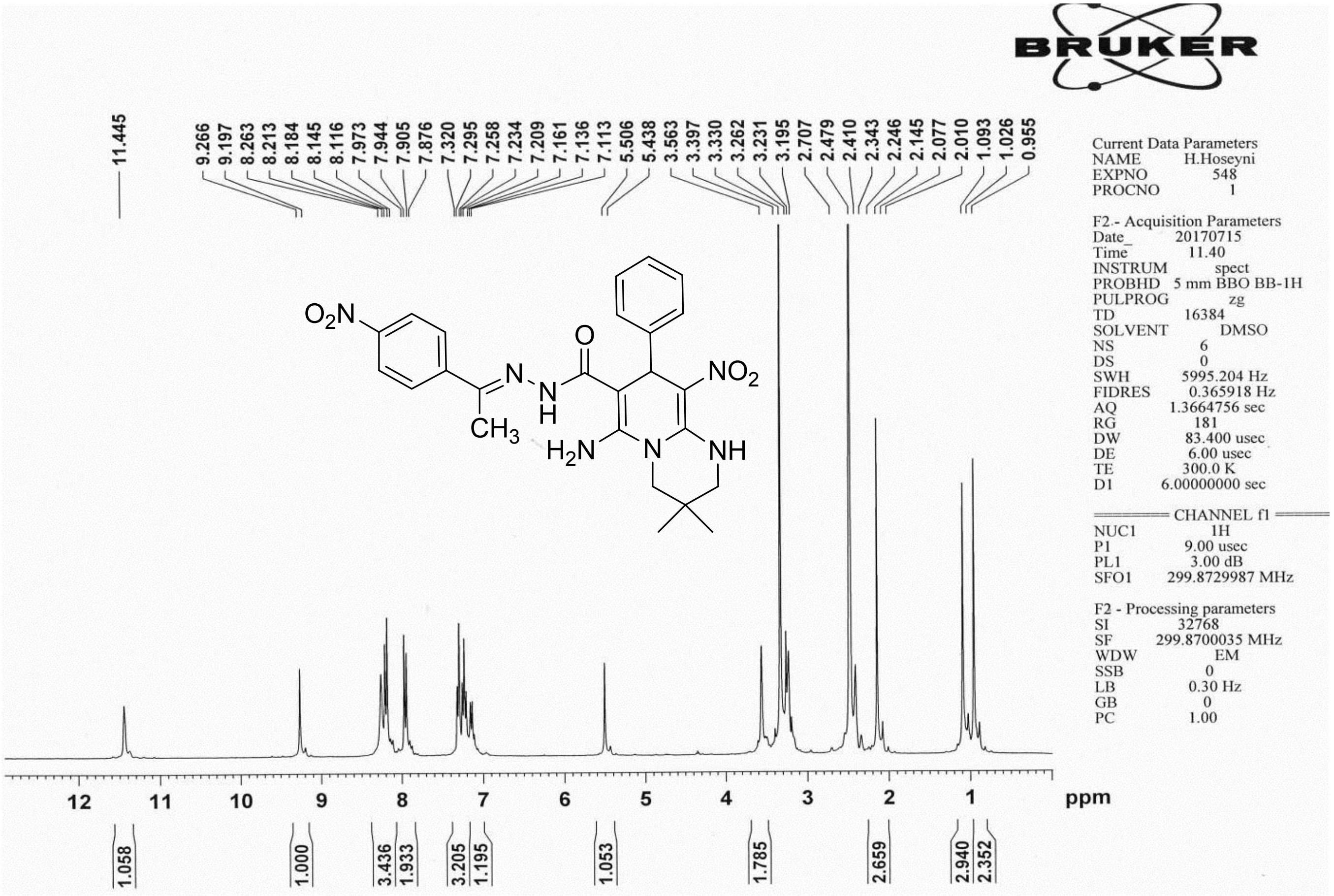
BRUKER
11.445
9.266
9.197
8.263
8.213
8.184
8.145
8.116
7.973
7.944
7.905
7.876
7.320
7.295
7.258
7.234
7.209
7.161
7.136
7.113
5.506
5.438
3.563
3.397
3.330
3.262
3.231
3.195
2.707
2.479
2.410
2.343
2.246
2.145
2.077
2.010
1.093
1.026
0.955
Current Data Parameters
NAME H.Hoseyni
EXPNO 548
PROCNO 1
F2 - Acquisition Parameters
Date_ 20170715
Time 11.40
INSTRUM spect
PROBHD 5 mm BBO BB-1H
PULPROG zg
TD 16384
SOLVENT DMSO
NS 6
DS 0
SWH 5995.204 Hz
FIDRES 0.365918 Hz
AQ 1.3664756 sec
RG 181
DW 83.400 usec
DE 6.00 usec
TE 300.0 K
D1 6.00000000 sec
CHANNEL f1
NUC1 1H
P1 9.00 usec
PL1 3.00 dB
SFO1 299.8729987 MHz
F2 - Processing parameters
SI 32768
SF 299.8700035 MHz
WDW EM
SSB 0
LB 0.30 Hz
GB 0
PC 1.00
O2N
O
NO2
N
N
H
CH3
H2N
N
NH
12
11
10
9
8
7
6
5
4
3
2
1
ppm
1.058
1.000
3.436
1.933
3.205
1.195
1.053
1.785
2.659
2.940
2.352

^{1}H NMR of 6q

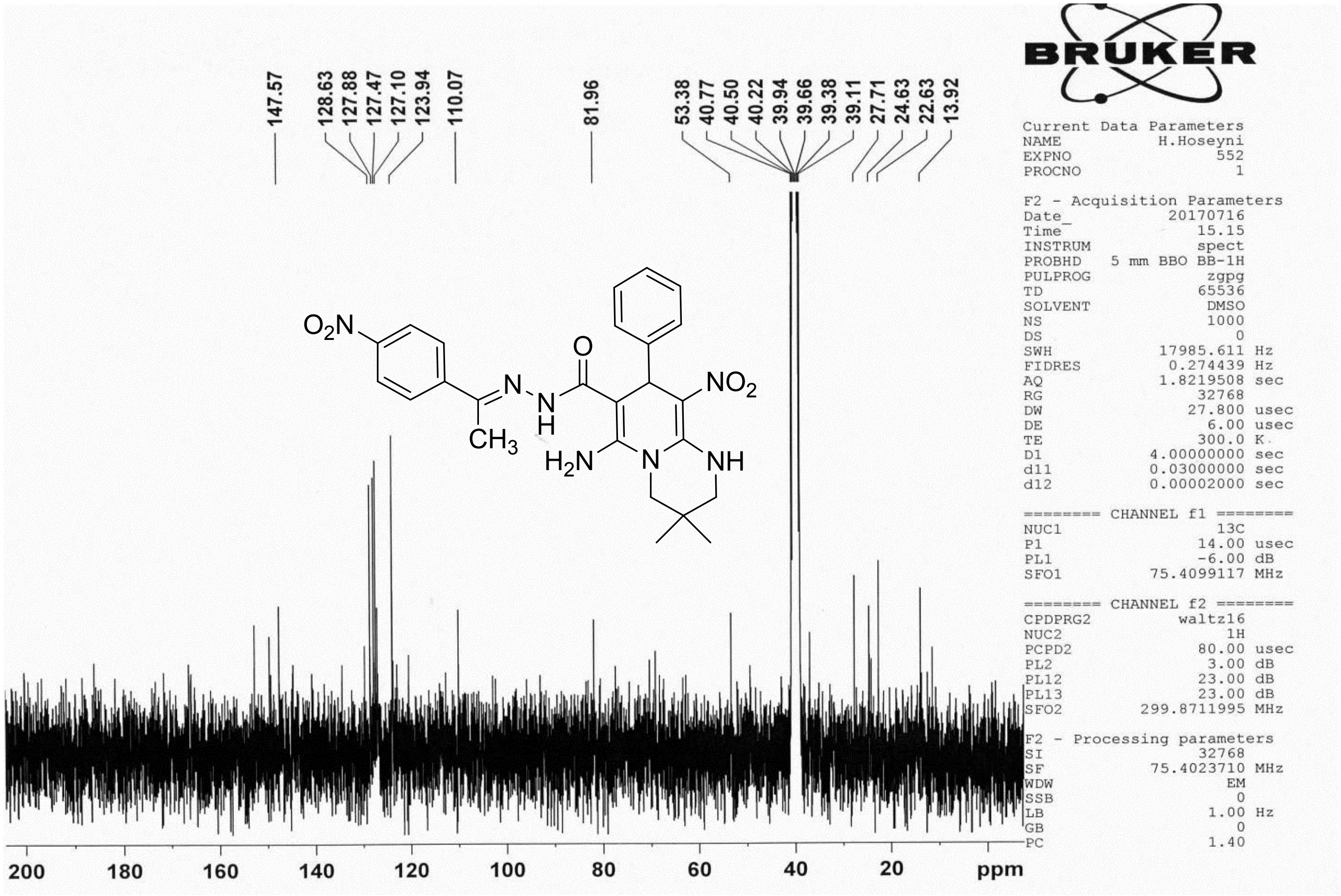
147.57
128.63
127.88
127.47
127.10
123.94
110.07
81.96
53.38
40.77
40.50
40.22
39.94
39.66
39.38
39.11
27.71
24.63
22.63
13.92
BRUKER
Current Data Parameters
NAME H.Hoseyni
EXPNO 552
PROCNO 1
F2 - Acquisition Parameters
Date_ 20170716
Time 15.15
INSTRUM spect
PROBHD 5 mm BBO BB-1H
PULPROG zgpg
TD 65536
SOLVENT DMSO
NS 1000
DS 0
SWH 17985.611 Hz
FIDRES 0.274439 Hz
AQ 1.8219508 sec
RG 32768
DW 27.800 usec
DE 6.00 usec
TE 300.0 K
D1 4.00000000 sec
d11 0.03000000 sec
d12 0.00002000 sec
======== CHANNEL f1 ========
NUC1 13C
P1 14.00 usec
PL1 -6.00 dB
SFO1 75.4099117 MHz
======== CHANNEL f2 ========
CPDPRG2 waltz16
NUC2 1H
PCPD2 80.00 usec
PL2 3.00 dB
PL12 23.00 dB
PL13 23.00 dB
SFO2 299.8711995 MHz
F2 - Processing parameters
SI 32768
SF 75.4023710 MHz
WDW EM
SSB 0
LB 1.00 Hz
GB 0
PC 1.40
200 180 160 140 120 100 80 60 40 20 ppm

^{13}C NMR of 6q

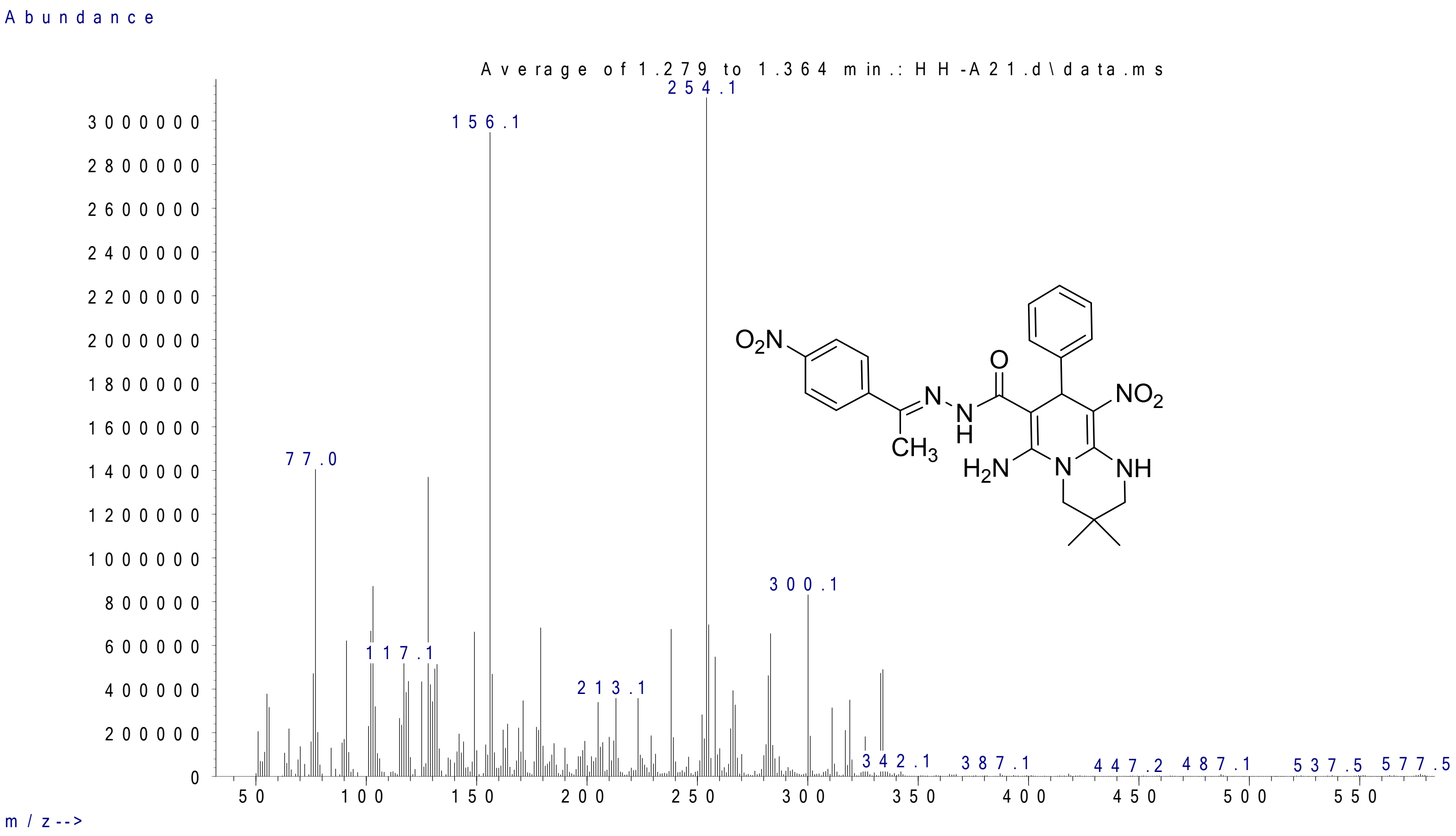
Abundance
Average of 1.279 to 1.364 min.: HH-A21.d\data.ms
254.1
156.1
77.0
117.1
213.1
300.1
342.1
387.1
447.2
487.1
537.5
577.5
3000000
2800000
2600000
2400000
2200000
2000000
1800000
1600000
1400000
1200000
1000000
800000
600000
400000
200000
0
50
100
150
200
250
300
350
400
450
500
550
m/z-->
O2N
O
NO2
N
N
H
CH3
H2N
N
NH

MS of 6q
